# Risks of Retinal Vascular Occlusions with the Systemic Use of Tyrosine Kinase Inhibitors

**DOI:** 10.1016/j.oret.2026.02.019

**Published:** 2026-03-04

**Authors:** Hejin Jeong, Sophie C. Yue, David C. Kaelber, Rishi P. Singh, Katherine E. Talcott

**Affiliations:** 1 Case Western Reserve University School of Medicine, Cleveland, Ohio.; 2 Center for Ophthalmic Bioinformatics, Cole Eye Institute, Cleveland Clinic, Cleveland, Ohio.; 3 Center for Clinical Informatics Research and Education, MetroHealth System, Cleveland, Ohio.; 4 Departments of Internal Medicine, Pediatrics, and Population and Quantitative Health Sciences, Case Western Reserve University School of Medicine, Cleveland, Ohio.; 5 Mass Eye and Ear, Mass General Brigham, Boston, Massachusetts.; 6 Department of Ophthalmology, Harvard Medical School, Boston, Massachusetts.; 7 Cleveland Clinic Cole Eye Institute, Cleveland, Ohio.; 8 Cleveland Clinic Lerner College of Medicine of Case Western Reserve University, Cleveland, Ohio.

**Keywords:** Anti-VEGF, Retinal artery occlusion, Retinal vascular occlusion, Retinal vein occlusion, Tyrosine kinase inhibitors

## Abstract

**Purpose:**

Central retinal vein occlusion (CRVO) and retinal perfusion defects have been described as potential risks associated with systemic tyrosine kinase inhibitor (TKI) therapy, but current evidence is limited to case reports. Therefore, this study aimed to better characterize any associations between systemic TKIs and the risks of retinal artery occlusion (RAO) and retinal vein occlusion (RVO).

**Design:**

Retrospective cohort study using multi-institutional electronic health records across the United States.

**Participants:**

Adults with cancer diagnoses and no prior history of retinal vascular occlusions or maculopathy, based on International Classification of Diseases (ICD) encounter diagnosis codes, were included. Patients with prescriptions for TKIs with and without anti-VEGF activities were created, and were each compared with matched control patients with non-TKI antineoplastic prescriptions.

**Methods:**

The study and control cohorts were propensity score—matched 1:1 based on demographic factors, comorbidities, other antineoplastic therapy, and cancer stages. The matched cohorts were then compared for having new-onset ICD encounter diagnosis codes of any RAO, central RAO, branch RAO, CRVO, and branch RVO (BRVO).

**Main Outcome Measures:**

Lifetime risks of RAO, central RAO, branch RAO, RVO, CRVO, and BRVO were compared via risk ratios (RRs). Rates of these outcomes within 3 years of antineoplastic initiation were also evaluated via hazard ratios (HRs). Differences in the outcome measures were considered statistically significant if the 95% confidence interval (CI) was ≤0.9 or ≥1.1.

**Results:**

While the anytime-risks of having RAOs and RVOs were comparable between the anti-VEGF TKI cohort and controls (each *n* = 6728), the anti-VEGF TKI cohort had a significant hazard of having CRVO within 3 years of TKI initiation (HR = 2.86, CI = 1.44—5.69). In contrast, while the non—anti-VEGF TKI cohort (*n* = 17 185) had a lower anytime-risk of having RAO (RR = 0.61, CI = 0.45—0.83) and BRVO (RR = 0.51, CI = 0.36—0.71) compared with the non-TKI controls (*n* = 17 185), there was no difference in the HR among outcomes between the 2 cohorts.

**Conclusions:**

Although non—anti-VEGF TKIs may have a more favorable safety profile with respect to BRVO and RAO compared with other antineoplastic therapies, patients initiating anti-VEGF TKIs may warrant closer ophthalmological monitoring for the incidence of CRVO during the first few years of treatment.

Over the last decades, tyrosine kinase inhibitors (TKIs) have revolutionized the landscape of oncological therapy and have been used to treat a wide range of cancers, including hematological malignancies, non-small cell lung cancer, melanoma, and various other solid tumors (renal, gastrointestinal, breast).^1,2^ By selectively targeting receptor tyrosine kinases, which are transmembrane proteins that regulate key cellular processes such as cell proliferation, differentiation, migration, and metabolism, TKIs offer antineoplastic benefit with relatively lower systemic toxicities.^1,2^

More recently, TKIs have also gained attention as potential treatments for retinal diseases such as diabetic macular edema and age-related macular degeneration.^3^ In this context, TKIs that inhibit VEGF receptors (VEGFRs) have been of particular interest because they can suppress aberrant angiogenesis,^1,2,4^ functioning similarly to anti-VEGF agents that are currently the standard therapy for various exudative retinal diseases.^5^

On the other hand, despite their generally superior safety profiles,^1^ several TKIs also have recognized adverse vascular effects that induce endothelial dysfunction.^2,6—8^ While systemic arterial macrovascular events, such as myocardial infarction, cerebrovascular occlusion, and pulmonary embolism, in patients with cardiovascular risk factors are most commonly reported in the literature,^2,6—8^ a growing body of case reports has also linked systemic TKI therapy to the development of central retinal vein occlusion (CRVO)^9—17^ and retinal arterial occlusion (RAO).^18,19^ Notably, all cases of CRVO were observed after the initiation of TKIs with anti-VEGF activity^9—17^ (henceforth referred to as “anti-VEGF TKIs”), which presents a clinical paradox against the established role of anti-VEGF injections in managing advanced retinal vein occlusion (RVO)^20^ and the current focus on TKIs with anti-VEGF properties for treating retinal diseases.

Because evidence of this toxicity has so far been limited to case reports, the generalizability of this potential relationship is unknown. Moreover, it remains unclear whether these observations reflect a true association with TKIs or were caused by comorbidities or patient-specific risk factors. To address these gaps, we aimed to analyze large cohorts of patients on TKIs to determine the risk of having RVOs and RAOs. In addition, we aimed to further investigate the potential role of anti-VEGF properties of systemic TKIs in retinal vascular occlusions through stratified analysis based on the anti-VEGF activity of TKIs.

## Methods

This retrospective cohort study analyzed data contained in the TriNetX US Collaborative Network, which provides deidentified electronic health records of *>*130 million patients from *>*70 US health care organizations. This study was exempt from informed consent and review by the Western Institutional Review Board, as all data are secondary to existing data, do not involve intervention or interaction with human subjects, and are deidentified per the deidentification standard defined in Section §164.514(a) of the Health Insurance Portability and Accountability Act Privacy Rule. The deidentification process is attested to through a formal determination by a qualified expert as defined in Section §164.514(b)(1) of the Health Insurance Portability and Accountability Act Privacy Rule and was refreshed in December 2020. This study was conducted in accordance with the Declaration of Helsinki and follows the Strengthening the Reporting of Observational studies in Epidemiology reporting guidelines for cohort studies.^21^

Queries used for the study were based on the International Statistical Classification of Diseases and Related Health Problems, Tenth Revision (ICD-10), Anatomical Therapeutic Chemical, RxNorm, Logical Observation Identifier Names and Codes, Current Procedural Terminology, Healthcare Common Procedure Coding System, and American Joint Committee on Cancer codes. Any diagnoses that were recorded in International Classification of Diseases 9 codes were converted to the corresponding ICD-10 codes based on the Centers for Medicare and Medicaid Services General Equivalence Mappings. Tables S1 and S2 (available at www.ophthalmologyretina.org) list the codes used in the study and medications included in each cohort and subcohort. The Appendix (available at www.ophthalmologyretina.org) summarizes the molecular targets and the indications for the included medications. While the remainder of this manuscript will describe patients and outcomes without explicitly referencing the codes to improve readability, it should be acknowledged that all queries were solely based on the presence or absence of encounter diagnosis or prescription codes.

**Figure 1.**
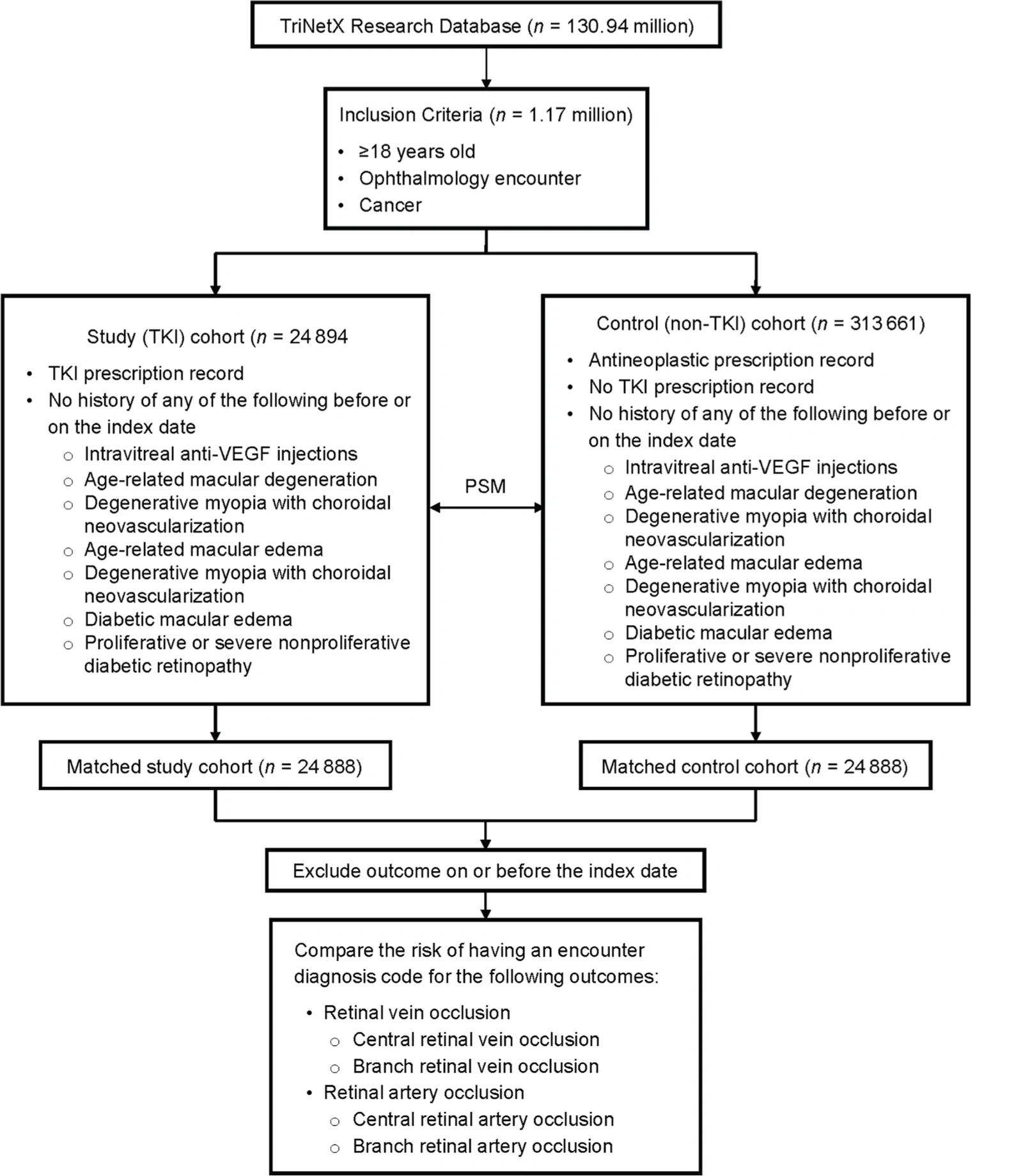
Consolidated Standards of Reporting Trials (CONSORT) diagram for the analysis comparing patients with prescription records for tyrosine kinase inhibitors (study cohort) versus those with nontyrosine kinase inhibitor antineoplastic agents (control cohort). PSM = propensity score matching; TKI = tyrosine kinase inhibitor

As illustrated in the Consolidated Standards of Reporting Trials diagram for the primary analysis (Fig 1), all analyses conducted in the study followed the general process of identifying eligible patients based on the inclusion and exclusion criteria, dividing them into study and control cohorts, matching the 2 cohorts via propensity score matching (PSM) to control for confounders, and comparing risks of having a relevant outcome. Outcomes of interest included new-onset diagnosis of any RVO, CRVO, branch RVO (BRVO), any retinal artery occulation (RAO), central RAO, branch RAO, and dental caries (negative control). This study used data recorded between January 1, 2005, and August 31, 2025. Data were accessed in September 2025.

The primary analysis included adults (≥18 years old) with cancer ICD-10 encounter diagnosis code(s) (Table S1) and compared patients with a prescription record for TKI antineoplastics (study cohort) to those with a prescription record for non-TKI antineoplastics (control cohort) (Fig 1, Table S1). We chose non-TKI antineoplastic users, as opposed to any cancer patients without a history of TKI prescription regardless of use of other antineoplastic agents, as the control cohort because the latter option would include patients who did not receive any antineoplastic therapy, which could introduce bias as these patients would more likely have lower disease severity, treatment tolerance, healthcare utilization level, surveillance intensity, comorbidities, and risk profiles compared with the study cohort. Furthermore, using non-TKI antineoplastic users allowed both the study and control cohorts to have a comparable definition of the index date, which was defined as the date of the TKI or non-TKI antineoplastic prescription. We only included patients with cancer ICD-10 encounter diagnosis codes because systemic TKIs are predominantly used for cancer (Table S1). To reduce potential misclassification bias and underestimation of outcomes due to missed diagnoses, we also required eligible patients to have evidence of receiving ophthalmic care, as indicated by the presence of ophthalmic visit or cataract/pseudophakia Current Procedural Terminology or ICD-10 codes (Table S1). We excluded patients with a baseline (i.e., on or before the index date) history of maculopathies―exudative age-related macular degeneration, diabetic macular edema, proliferative diabetic retinopathy, or degenerative myopia with choroidal neo-vascularization―or intravitreal anti-VEGF injection to minimize potential confounders (Table S1).

The study and control cohorts were matched on demographical factors, baseline body mass index, socioeconomic drivers of health, smoking status, alcohol misuse, comorbidities associated with retinal thrombosis (cardiovascular, diabetes, thrombophilic conditions, glaucoma), medications including immunosuppressants and other antineoplastics, radiation therapy, and cancer stages (Table S2). After excluding patients who had a baseline history of the outcomes, the matched cohorts were compared to evaluate the risk ratio (RR) of having a diagnosis code of the outcomes. Given that most previous case reports observed retinal vascular occlusions within 3 years of TKI initiation,^9—17^ we additionally analyzed the hazard ratios (HRs) of outcomes within 3 years of the index date. We excluded deaths occurring within this observation period.

Because most prior RVO cases after TKI initiation involved TKIs with anti-VEGF activity,^9—17^ we additionally conducted subanalyses by stratifying the TKI cohort based on the anti-VEGF property of the TKIs to assess whether this modified the associations between TKI and retinal vascular events (Table S1). Both subcohorts excluded anyone with a prescription record for both anti-VEGF and non—anti-VEGF TKIs. Both subcohorts were compared with the non-TKI controls using the same procedure used for the primary analysis. Analysis directly comparing the anti-VEGF versus non—anti-VEGF TKI subcohorts could not be performed due to limited sample size.

Additionally, given that breakpoint cluster region-Abelson murine leukemia (BCR-ABL) inhibitors (e.g., nilotinib, ponatinib, dasatinib) have been associated with systemic vascular risks in previous studies,^2,6,7,17^ we explored BCR-ABL TKIs in a secondary subanalysis to assess whether any observed significant results in the anti-VEGF subanalysis were unique to anti-VEGF activity. This subanalysis followed the same methodological approach as the anti-VEGF subanalysis.

Lastly, we performed sensitivity analyses for the primary analysis by limiting the patients to those with TKI (study) or non-TKI (control) antineoplastic prescription records for a minimum duration of 6 months, 1 year, and 2 years.

### Statistical Analysis

All statistical analyses were performed using the analytic tools built into the research platform. Propensity score matching analysis utilized a one-to-one greedy matching algorithm with a caliper of 0.1 pooled standard deviations. Propensity score matching was considered successful if the absolute standardized mean difference for each covariate was *<*0.1. We calculated RRs and HRs using logistic regression and Kaplan-Meier analysis, respectively. To account for multiple sampling, statistical significance was defined as a confidence interval (CI) of ≤0.9 and ≥1.1. We used Microsoft Excel and R (version 4.3.3)^22^ on RStudio (version 4.5.1)^23^ to visualize the data.

## Results

**Figure 2.**
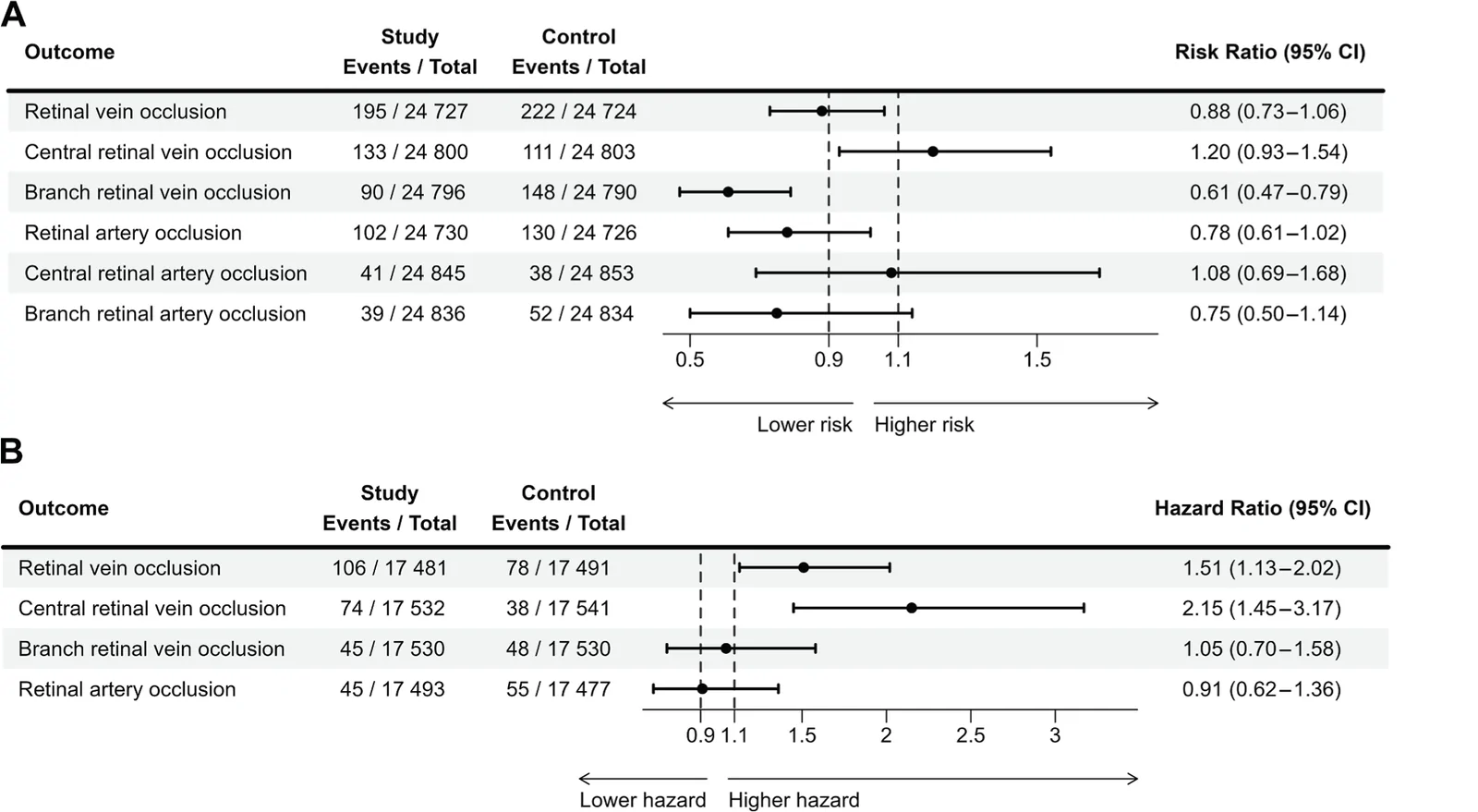
Risks (**A**) and 3-year hazards (**B**) of having retinal vascular occlusions in patients with prescription records for tyrosine kinase inhibitors (study cohort) versus those with nontyrosine kinase inhibitor antineoplastic agents (control cohort). Insufficient sample size for the 3-year hazard ratio analysis for central and branch retinal artery occlusions. CI = confidence interval.

After successful PSM (Table S3A-F, available at www.ophthalmologyretina.org), we identified 24 888 patients in the TKI cohort (mean age: 64.6 ± 14.6 years, 47.7% female) and 24 888 matched controls (mean age: 64.6 ± 14.3 years, 47.0% female) (Table S3A). Although the 2 cohorts did not differ in the risks of having central RAO, branch RAO, or CRVO any time after antineoplastic initiation, the TKI cohort had a lower risk of having BRVO (RR = 0.61, CI = 0.47–0.79) (Fig 2A). On the other hand, the HR analysis revealed a significantly higher hazard of having CRVO within 3 years in the TKI cohort relative to the non-TKI controls (HR = 2.15, CI = 1.45—3.17) (Fig 2B). These results were consistent with those of the sensitivity analyses (Figs S1—S3, available at www.ophthalmologyretina.org), except for the 6-month analysis, which did not show a significant difference in the hazards of having CRVO based on our significance threshold (HR = 1.62, CI = 1.02—2.56) (Fig S1B).

**Figure 3.**
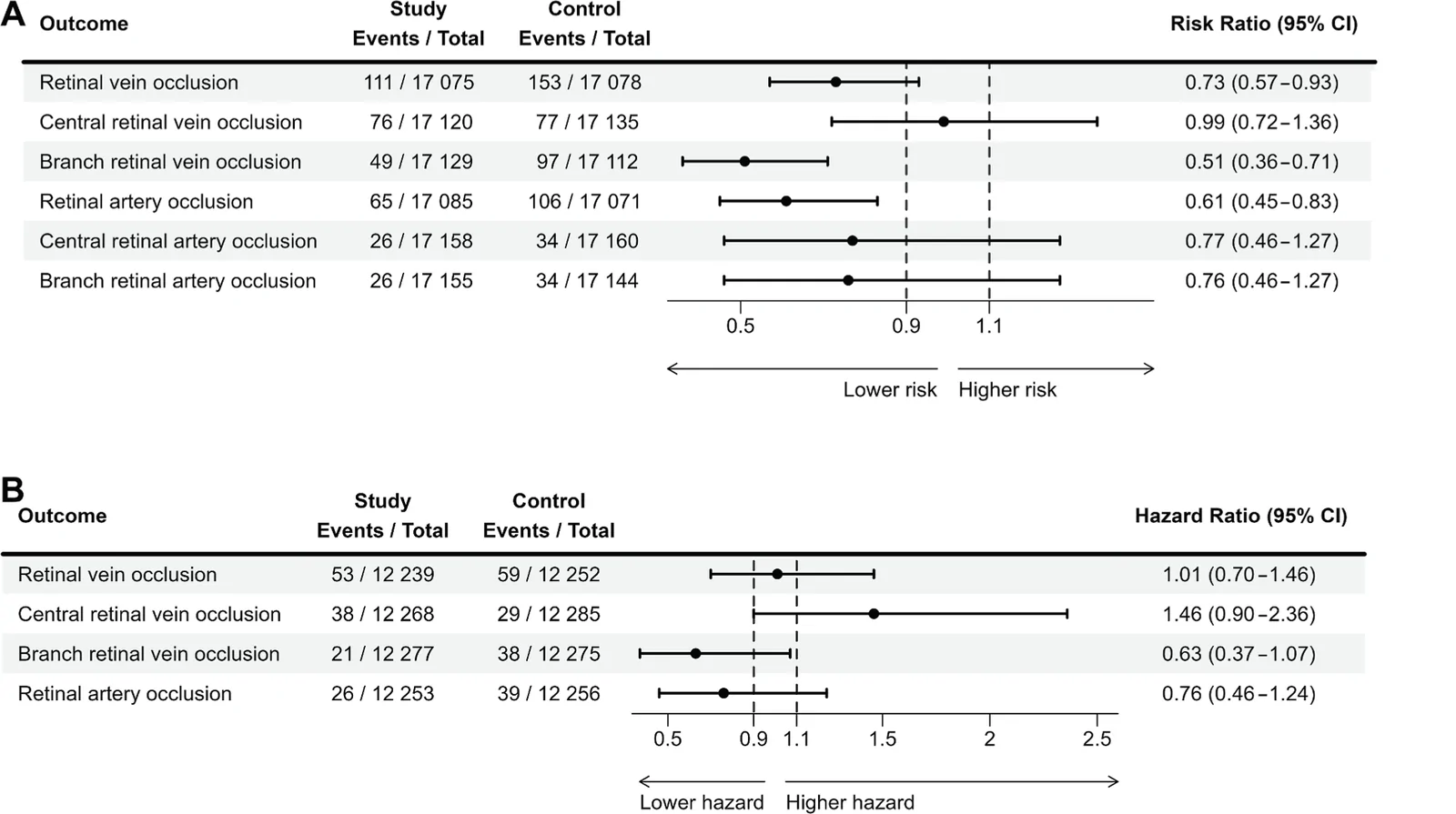
Risks (**A**) and 3-year hazards (**B**) of having retinal vascular occlusions in patients with prescription records for tyrosine kinase inhibitors without anti-VEGF activity (study cohort) versus those with nontyrosine kinase inhibitor antineoplastic agents (control cohort). Insufficient sample size for the 3-year hazard ratio analysis for central and branch retinal artery occlusions. CI = confidence interval.

**Figure 4.**
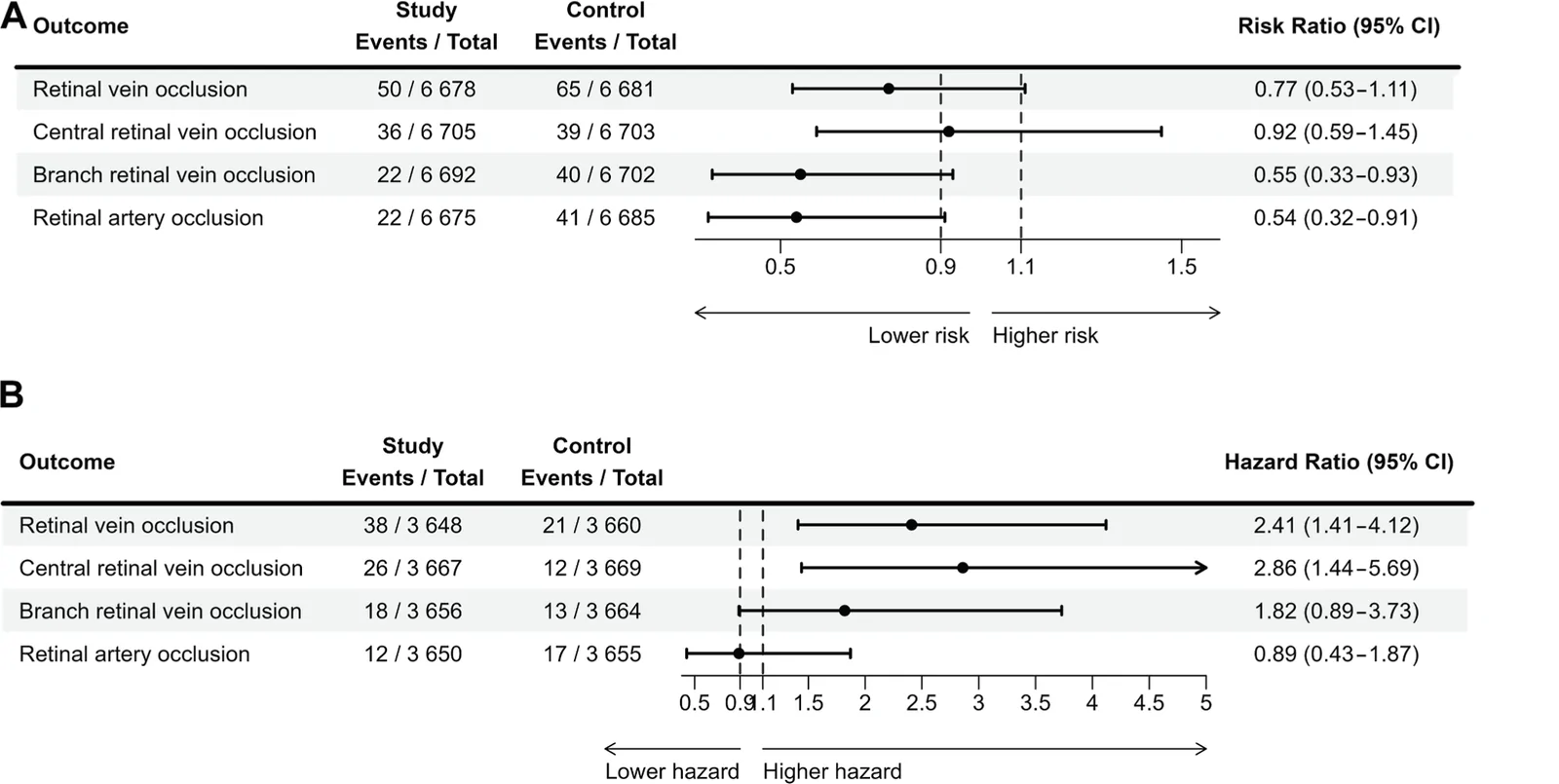
Risks (**A**) and 3-year hazards (**B**) of having retinal vascular occlusions in patients with prescription records for tyrosine kinase inhibitors with anti-VEGF activity (study cohort) versus those with nontyrosine kinase inhibitor antineoplastic agents (control cohort). Insufficient sample size for the 3-year hazard ratio analysis for central and branch retinal artery occlusions. CI = confidence interval.

In the subanalyses comparing the relative risks of having retinal vascular occlusions any time after antineoplastic initiation, we found that, while the risk of BRVO was similarly lower in the non—anti-VEGF TKI subcohort compared with the non-TKI controls (RR = 0.51, CI = 0.36—0.71) (Fig 3A), no such difference was observed when the anti-VEGF TKI subcohort was compared with the non-TKI controls (Fig 4A). Conversely, while the non—anti-VEGF TKI subcohort did not differ from the control cohort on the hazards of having any vascular occlusion outcomes within 3 years of TKI initiation (Fig 3B), the anti-VEGF TKI subcohort had a higher hazard of having CRVO (HR = 2.86, CI = 1.44–5.69) (Fig 4B), similar to the primary analysis. Finally, in addition to BRVO, the non—anti-VEGF TKI subcohort had a marginally reduced risk of having RAO (RR = 0.61, CI = 0.45—0.83), though the association became nonsignificant when the RAO subtypes (central RAO and branch RAO) were separately analyzed (Fig 3A).

**Figure 5.**
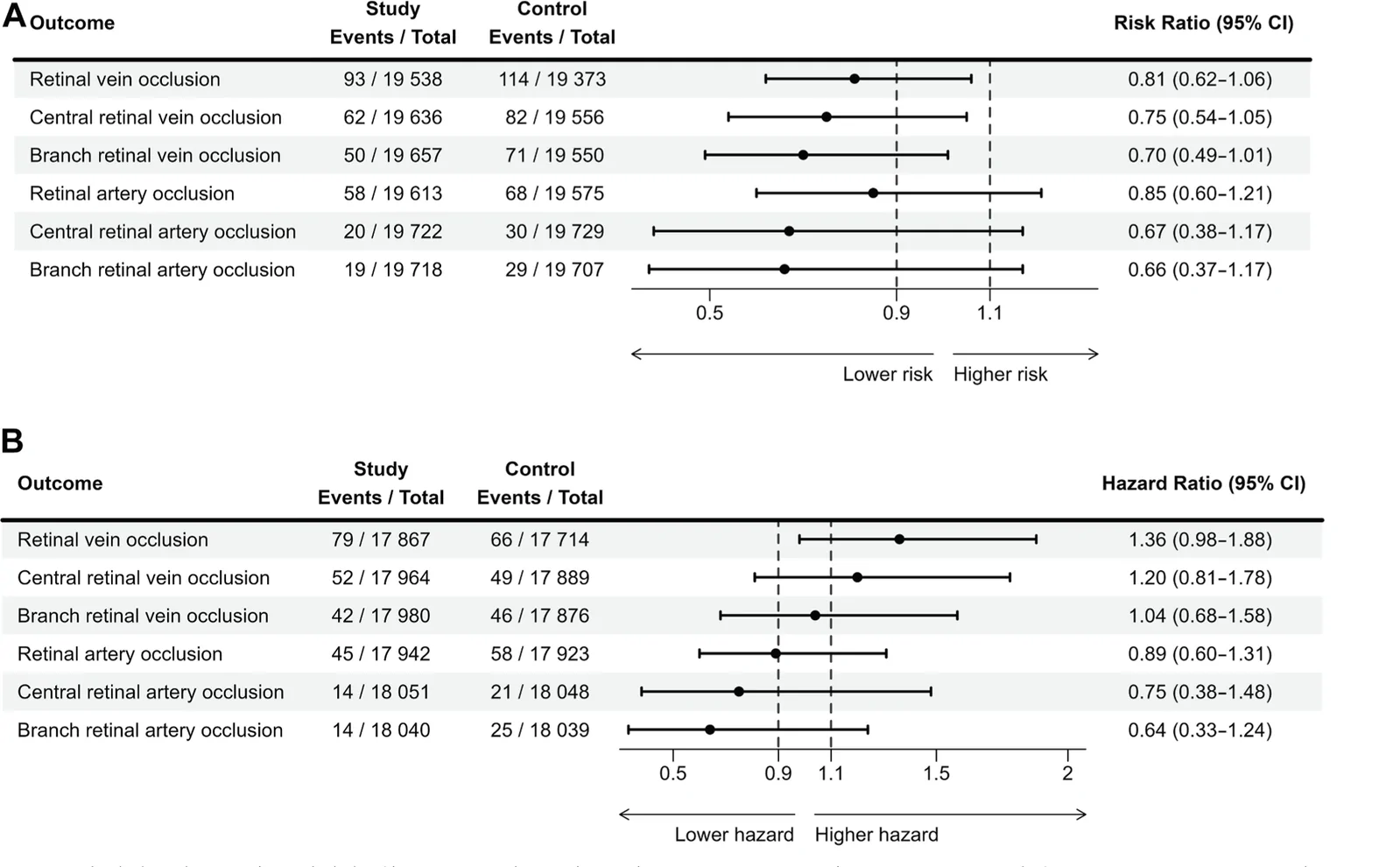
Risks (**A**) and 3-year hazards (**B**) of having retinal vascular occlusions in patients with prescription records for non—BCR-ABL tyrosine kinase inhibitors (study cohort) versus those with nontyrosine kinase inhibitor antineoplastic agents (control cohort). BCR-ABL = breakpoint cluster region-Abelson murine leukemia; CI = confidence interval.

**Figure 6.**
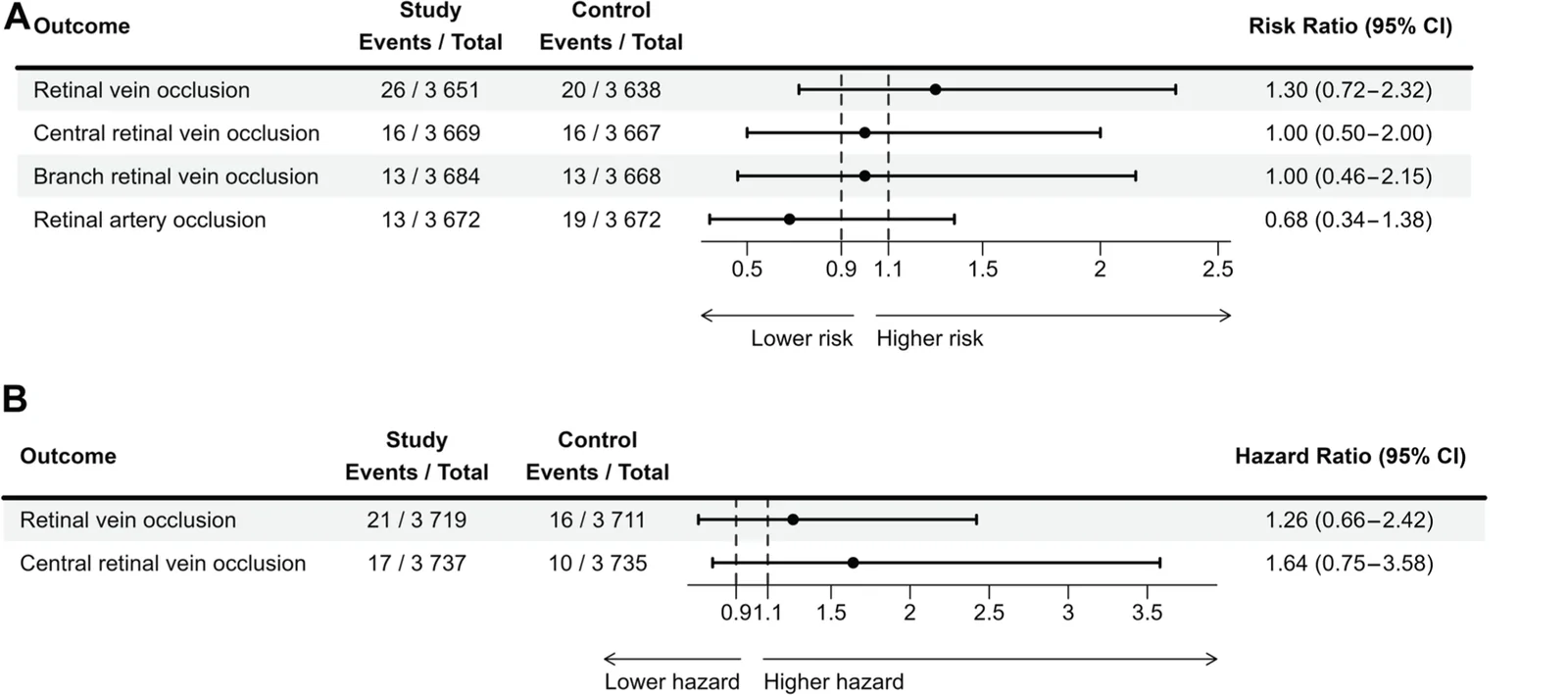
Risks (**A**) and 3-year hazards (**B**) of having retinal vascular occlusions in patients with prescription records for BCR-ABL tyrosine kinase inhibitors (study cohort) versus those with nontyrosine kinase inhibitor antineoplastic agents (control cohort). Insufficient sample size for the 3-year hazard ratio analysis for central and branch retinal artery occlusions. BCR-ABL = breakpoint cluster region-Abelson murine leukemia; CI = confidence interval.

**Figure 7.**
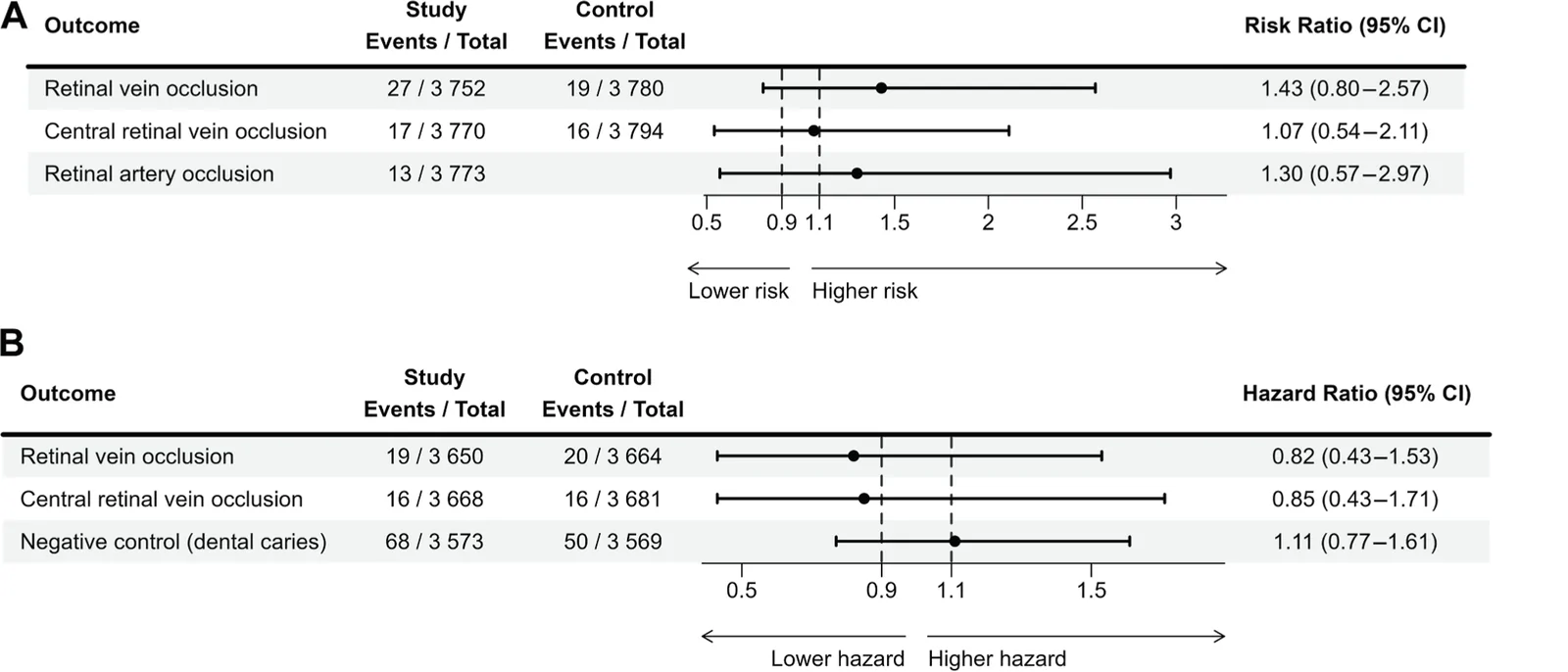
Risks (**A**) and 3-year hazards (**B**) of having retinal vascular occlusions in patients with prescription records for BCR-ABL (study cohort) versus those with non—BCR-ABL (control cohort) tyrosine kinase inhibitors. Insufficient sample size for the analysis of branch retinal vein occlusion and central and branch retinal artery occlusions. BCR-ABL = breakpoint cluster region-Abelson murine leukemia; CI = confidence interval.

On the other hand, none of the BCR-ABL subanalyses demonstrated evidence of association between BCR-ABL TKIs and risks of any type of retinal vascular occlusions (Figs 5—7).

The negative control analyses revealed no significant differences in the risks or hazards of having dental caries between the study and control cohorts for the above analyses (Table S4, available at www.ophthalmologyretina.org), supporting the validity of their findings.

## Discussion

In this study of large, multiple-institutional cohorts of patients with cancer in the United States, we found that, while the overall risk of retinal vascular occlusions with systemic TKI use is low and may even be associated with a lower risk of BRVO and RAO for non—anti-VEGF TKIs, anti-VEGF TKIs are associated with a significantly higher hazard of having CRVO within the first 3 years of initiation.

The increased hazard of CRVO observed in the anti-VEGF TKI cohort aligns with the previous case reports of CRVO and CRVO-like features within 3 years of initiation of sorafenib, apatinib, regorafenib, axitinib, sunitinib, lenvatinib, or anlotinib, all of which have anti-VEGF properties.^9—17^ One possible explanation for this result is the hypertension―a main risk factor for RVO^20^―known to occur with the inhibition of VEGFRs, which normally activates the phosphatidylinositol 3-kinase/protein kinase B-driven production of vasodilatory nitric oxide and prostaglandins.^2,7,24^

Furthermore, as a part of the mitogen-activated protein kinase pathway, VEGFR activation also triggers downstream BRAF-mitogen-activated protein kinase kinase/extracellular signal-regulated kinase signaling cascade that drives cell proliferation and angiogenesis. Although inhibition of this pathway offers oncologic and antineovascular benefits, it also downregulates factors critical for endothelial repair while inducing pro-atherogenic surface adhesion molecules and coagulation cascade, ultimately promoting prothrombotic and atherosclerotic states with endothelial and pericyte dysfunction.^2,7,24^ Our observation that the anti-VEGF TKI cohort had higher hazards of CRVO compared with BRVO within the first 3 years (Fig 4B) further corroborates this hypothesis, as evidence suggests that systemic hypercoagulability may play a greater role in CRVO than BRVO.^25^ The anatomical location of the central retinal vein, which passes through the confined space of the lamina cribrosa, may also contribute to its relatively higher susceptibility to endothelial injury and thrombotic risk factors than in BRVO, which are most commonly due to compression by the adjacent atherosclerotic arteries at arteriovenous crossings.^20^

Nonetheless, current literature presents mixed findings on venous thrombotic risks associated with anti-VEGF TKIs. For instance, in contrast to our findings and several studies that reinforce the venous thrombotic risks of anti-VEGF therapy,^2,4,7,8,24^ a meta-analysis of 4430 anti-VEGF TKI users from studies with median progression-free survival under 3 years found no significant increase in the risk of systemic venous thromboembolism compared with non-TKI users.^26^ A meta-analysis of clinical trials comparing anti-VEGF TKIs against placebo controls further supported this conclusion, demonstrating no difference in venous thromboembolism risks between the cohorts.^27^ This suggests that the underlying pathophysiological link between anti-VEGF TKIs and CRVO may involve distinct characteristics of the retinal vascular environment.

For instance, the blood-retina barrier restricts the entry of many factors in the systemic circulation that promote vasodilation and endothelial repair, such as circulating endothelial progenitor cells, growth factors, antioxidants, and anti-inflammatory cytokines.^28,29^ Therefore, retinal endothelial cells may be particularly vulnerable to the insults caused by anti-VEGF TKIs and less able to recover from the resulting microvascular injury compared with those in systemic vessels.^28,29^ This also makes the retinal vascular flow especially dependent on the local production of vasodilatory nitric oxide and prostacyclins by the retinal endothelial cells.^28,30^ Because anti-VEGF inhibition reduces bioavailability of these vasodilators and damages the endothelial cells and pericytes,^7,24,30,31^ retinal veins may be more sensitive to anti-VEGF—induced vascular stasis and hypertension compared with systemic veins. These effects can additionally be compounded by the downregulation of the RAF-mitogen-activated protein kinase kinase/extracellular signal-regulated kinase pathway, especially given that RVO and retinal pigment epithelial dysfunction are established side effects of anticancer agents that inhibit this pathway.^16,30,32,33^

On the other hand, our subanalysis of BCR-ABL inhibitors interestingly did not demonstrate an increased hazard for retinal vascular events, even in the head-to-head comparative analysis (Fig 5B). Our null findings―despite the well-recognized systemic endothelial stress and prothrombotic changes that occur with BCR-ABL inhibitor therapies^2,6,7,17^―support the hypothesis that inhibiting VEGFRs and theirs downstream pathways may play a particularly significant role in retinal hemodynamic regulation, rather than a class-wide effect of all vasotoxic TKIs.

Whether the potential short-term risk of CRVO from TKI, as indicated in our results, extends to intraocular TKI therapy for retinal pathologies remains to be elucidated. Currently, intravitreal injections of anti-VEGF molecules (e.g., aflibercept, bevacizumab, ranibizumab), which interrupt pathological neovascularization by sequestering VEGF ligands that bind to the extracellular domains of the VEGFRs,^3,34^ are the mainstay of therapy against exudative complications of several retinal diseases such as age-related macular degeneration, diabetic macular edema, and RVO. ^5^ As anti-VEGF TKIs inhibit the VEGF pathway via antagonism of the intracellular domains of VEGFRs, anti-VEGF TKIs have received attention, especially for their potential to offer a dual mechanism to inhibit neovascularization when combined with the traditional anti-VEGF molecules.^3,35^ Retinal or systemic vascular occlusions have not been recognized as major adverse effects of anti-VEGF therapy and intraocular TKIs, although the available safety data on the latter is currently limited to small cohorts with short-term outcomes in controlled clinical trial settings.^3,36^ As research in this area is still in the nascent stages, continued investigations in larger, longitudinal studies are needed to clarify the retinal vascular risk profiles of intraocular TKIs.

The decreased risks of BRVO and RAO that we observed in the non—anti-VEGF TKI cohort compared with non-TKI controls may be explained by several factors. First, as the absence of anti-VEGF activity likely spared the normal vasodilatory and homeostatic effects of VEGF, the overall improvement in cancer severity achieved by TKI therapy could have sufficiently reduced the cancer-associated thrombogenicity without being offset by the vascular toxicity induced by VEGF inhibition. This is especially possible given that our RR analysis, unlike the HR analysis, did not limit the outcome observation window and thus compared the lifetime risk of retinal vascular occlusions rather than acute risks. Second, it should be noted that our control cohort comprised patients with prescription records for non-TKI antineoplastics, rather than patients with no antineoplastic therapy. Since several non-TKI antineoplastics have known systemic and retinal thrombotic and prohypertensive effects,^2,4,7,30,32^ the observed risk of retinal vascular occlusion in the TKI cohort may have been lower relative to those of non-TKI antineoplastics.

### Limitations

This study leveraged a large patient sample of a multiracial population, utilized rigorous patient selection criteria and PSM, and performed negative control and sensitivity analyses to enhance its validity and generalizability. Nonetheless, it also has limitations to consider. The study’s reliance on medical codes could have introduced misclassification bias, inaccuracies in PSM, and underrepresentation or overrepresentation of the outcomes due to miscoding, incomplete documentation, and variances in coding practices between healthcare institutions and clinicians. This is particularly relevant for lifestyle factors that affect retinal vascular risk, as information on lifestyle habits is not well captured in structured electronic health record data, and socioeconomic status is often underreported and underdocumented.^37,38^ Likewise, analyses could not be stratified by dosage, frequency, and individual types of TKIs due to limited sample size and incomplete data.

While the small outcome counts offer valuable insight into the rarity of retinal vascular occlusions as adverse effects of TKIs, they also resulted in wide CIs in the HR analyses and subanalyses. Therefore, the precision and reliability of the estimates may be limited for these analyses, although the association with CRVO―the key finding of this study―was nonetheless statistically significant even under the more rigorous threshold significance we used (CI *>*1.1). As the nonsignificant HR of BRVO with anti-VEGF TKIs could have been due to the underpowered nature of our analysis, the apparent preferential risk of CRVO (compared with BRVO) with anti-VEGF TKIs should be interpreted with caution.

It is important to note that most TKIs are multitargeted agents, and most agents categorized as anti-VEGF in our study are not pure VEGF-only inhibitors (Appendix and Table S1). For instance, lenvatinib, nintedanib, regorafenib, and ponatinib additionally inhibit fibroblast growth factor receptors,^6,16^ which, as with VEGFRs, activate the downstream BRAF-mitogen-activated protein kinase kinase/extracellular signal-regulated kinase pathway; sorafenib and regorafenib both directly inhibit BRAF (Appendix).^2,12^ Therefore, the observed elevated hazards of CRVO in the anti-VEGF cohort should not be interpreted as a result of VEGFR inhibition alone, but rather the combined disruption of the mitogen-activated protein kinase signaling axis―and potentially other pathways―from such off-target inhibition. Similarly, it is possible that our BCR-ABL TKI subanalysis failed to reach significance because the inclusion of multiple BCR-ABL TKIs into a single aggregate subcohort could have diluted agent-specific effects. Although we could not isolate selective anti-VEGF TKIs or stratify our analyses by individual TKI types due to insufficient sample sizes, considering the heterogeneous vascular risk profiles across TKIs,^6,7^ investigating the risk associated with individual agents would be a valuable area of future study.

Because TKIs are typically indicated for malignancy,^1^ we limited our cohorts to those with a cancer diagnosis to minimize potential confounding cancer-associated thrombosis. However, this also reduced our generalizability to patients without cancers or with isolated benign neoplasms. Conversely, although we additionally matched patients on cancer stages, other anticancer treatments, and cancer types that are particularly known to be thrombogenic, residual uncontrolled cancer-related factors could have affected our results. Though our ability to specifically investigate the impact of concomitant therapy on the relationship between TKIs and retinal vascular occlusions was limited by sample size, such stratified analysis on broader patient populations would be another particularly worthwhile area for future investigations, as concomitant therapy has been shown to be a significant modifier of the systemic thrombotic risks of TKIs.^26,27^ Finally, as noted previously, because we used non-TKI antineoplastics, rather than a placebo, as our control cohort, the retinal vascular impact of such non-TKI antineoplastics must be considered when evaluating the results.

## Conclusion

In summary, this matched cohort analysis of medical records of a large number of aggregated electronic health records of patients with cancer found that those with a record of receiving systemic TKIs had a low, but significantly increased hazard of having acute CRVO relative to controls on non-TKI antineoplastic therapy. Specifically, this association was observed only for TKIs with anti-VEGF properties, reflecting the trends observed in several recent case reports. TKIs without anti-VEGF properties had no such association with acute retinal vascular events and instead were associated with a lower lifetime risk of BRVO and RAO, which may reflect their potential benefit against malignancy-associated retinal thrombosis compared with non-TKI antineoplastics. Prospective studies utilizing retinal imaging and more stringent control against confounders would help refine these implications of this explorative study and further inform monitoring strategies for patients initiating TKI therapy.

## Data Availability:

The data that support the findings of this study are available from TriNetX, LLC, but third-party restrictions apply to the availability of these data. The data were used under license for this study, with restrictions that do not allow for the data to be redistributed or made publicly available. However, for accredited researchers, the TriNetX data is available for licensing at TriNetX, LLC. Data access may require a data sharing agreement and may incur data access fees.

## Supplementary Material

1

2

3

4

5

6

7

8

9

10

11

12

13

14

15

16

17

18

19

20

21

22

23

24

25

26

27

28

29

Supplemental material available at www.ophthalmologyretina.org.

## Abbreviations and Acronyms:

BCR-ABL: breakpoint cluster region-Abelson murine leukemia
BRVO: branch retinal vein occlusion
CI: confidence interval
CRVO: central retinal vein occlusion
HR: hazard ratio
ICD: International Classification of Diseases
PSM: propensity score matching
RAO: retinal artery occlusion
RR: risk ratio
RVO: retinal vein occlusion
TKI: tyrosine kinase inhibitor
VEGFR: VEGF receptor
