# Supplementary material for: Risks of Retinal Vascular Occlusions with the Systemic Use of Tyrosine Kinase Inhibitors
Source: Ophthalmol Retina. Author manuscript; Available in PMC 2026 Sep 21. (PMC13591267; doi:10.1016/j.oret.2026.02.019)
Supplement: Supplementary file 1 [file NIHMS2194774-supplement-1.pdf]

Appendix: Targets and indications of tyrosine kinase inhibitors included in the study

TKI Name	Molecular Targets	FDA-Approved Indications
Anti-VEGF TKI:		
Axitinib	VEGFR-1, VEGFR-2, VEGFR-3	RCC, Thyroid cancer
Fruquintinib	VEGFR-1, VEGFR-2, VEGFR-3	Metastatic colorectal cancer
Tivozanib	VEGFR-1, VEGFR-2, VEGFR-3	RCC
Nintedanib	VEGFR-1 to 3, FGFR1-3, PDGFR-α/β, FLT-3, Src, RET, TRKA/C, DDR1/2	IPF, NSCLC
Pazopanib	VEGFR-1, VEGFR-2, VEGFR-3, PDGFR-α, PDGFR-β, c-KIT	RCC
Regorafenib	VEGFR-1 to 3, TIE-2, PDGFR-β, FGFR-1, RET, KIT, BRAF	CRC, HCC, GIST
Sorafenib	VEGFR-1 to 3, PDGFR-α/β, FLT-3, RAF-1, BRAF, c-KIT, RET	Thyroid cancer, HCC, RCC
Sunitinib	VEGFR-1 to 3, PDGFR-α/β, FLT-3, CSF-1R, RET, c-KIT	GIST, Neuroectodermal tumors, RCC, Sarcomas
Lenvatinib	VEGFR-1, VEGFR-2, VEGFR-3, FGFR1-4, PDGFR-α, KIT, RET	Endometrial cancer, HCC, RCC, Thyroid cancer
Vandetanib	VEGFR-2, EGFR, RET	Medullary thyroid cancer
Cabozantinib	VEGFR-2, FLT-3, c-KIT, RET, MET	HCC, RCC, Thyroid cancer
Ponatinib	VEGFR, PDGFR, FGFR, SRC, BCR-ABL, FLT-3, c-KIT	CML, Ph+ ALL
Non-anti-VEGF TKI:		
Deucravacitinib	TYK2	Plaque psoriasis
Avapritinib	PDGFR-α, KIT	GIST, Systemic mastocytosis
Capmatinib	MET	NSCLC (MET exon 14)
Pemigatinib	FGFR1, FGFR2, FGFR3	Cholangiocarcinoma, MLN
Ripretinib	PDGFR-α, c-KIT	GIST
Tucatinib	HER2	Breast cancer, CRC
Entrectinib	TRKA, TRKB, TRKC, ROS1	NTRK+ Solid tumors, ROS1+ NSCLC
Erdafitinib	FGFR1, FGFR2, FGFR3, FGFR4	Urothelial carcinoma
Fedratinib	JAK2, FLT-3	Myelofibrosis
Pexidartinib	CSF-1R, c-KIT, FLT-3	Tenosynovial giant cell tumor
Zanubrutinib	BTK	Lymphomas, Waldenström macroglobulinemia, CLL/SLL
Binimetinib	MEK1, MEK2	Melanoma (BRAF+)
Dacomitinib	EGFR, HER2, HER4	NSCLC (EGFR+)
Fostamatinib	SYK	Chronic immune thrombocytopenia
Gilteritinib	FLT-3, AXL	AML (FLT-3+)
Larotrectinib	TRKA, TRKB, TRKC	NTRK+ Solid tumors
Lorlatinib	ALK, ROS1	ALK+ NSCLC, ROS1+ NSCLC
Acalabrutinib	BTK	Lymphomas, Waldenström macroglobulinemia
Brigatinib	ALK, ROS1, EGFR	ALK+ NSCLC
Midostaurin	FLT-3, c-KIT, PDGFR, PKC	AML, Systemic mastocytosis
Neratinib	EGFR, HER2, HER4	Breast cancer
Alectinib	ALK, RET	ALK+ NSCLC
Cobimetinib	MEK1, MEK2	Melanoma (BRAF+)
Osimertinib	EGFR	NSCLC (EGFR+)
Ceritinib	ALK	ALK+ NSCLC
Afatinib	EGFR, HER2, HER4	NSCLC (EGFR+)
Ibrutinib	BTK, ITK, TEC	CML, Lymphomas, Waldenström macroglobulinemia, GVHD
Bosutinib	BCR-ABL, SRC	CML
Crizotinib	ALK, ROS1, MET	ALK+ NSCLC, ROS1+ NSCLC
Ruxolitinib	JAK1, JAK2	Myelofibrosis, Polycythemia, GVHD
Nilotinib	BCR-ABL, c-KIT, PDGFR	CML
Dasatinib	BCR-ABL, SRC, c-KIT, PDGFR	CML, Ph+ ALL
Erlotinib	EGFR	NSCLC, pancreatic cancer
Gefitinib	EGFR	NSCLC (EGFR+)
Selpercatinib	RET	NSCLC, Medullary thyroid cancer, Solid tumors
Imatinib	BCR-ABL, KIT, PDGFR, ABL, DDR1	CML, Ph+ ALL, GIST, Mastocytosis
Tepotinib	MET	NSCLC (MET exon 14)
Asciminib	BCR-ABL	CML
Mobocertinib	EGFR	NSCLC (EGFR exon 20)
Lazertinib	EGFR	NSCLC
Lapatinib	EGFR, HER2	Breast cancer
Pacritinib	JAK2, FLT3, IRAK1, ACVR1/ALK2, CSF1R	Myelofibrosis
Momelotinib	JAK1, JAK2, ACVR1	Myelofibrosis
Pirtobrutinib	BTK	Mantle cell lymphoma, CLL/SLL
Futibatinib	FGFR1, FGFR2, FGFR3, FGFR4	Cholangiocarcinoma
Pralsetinib	RET	NSCLC
Quizartinib	FLT3, KIT	AML
Capivasertib	AKT1, AKT2, AKT3	Breast cancer
Repotrectinib	ROS1, TRKA, TRKB, TRKC	NSCLC, Solid tumors

Abbreviations: ACVR1, activin A receptor type 1; AKT, protein kinase B; ALK, anaplastic lymphoma kinase; ALL, acute lymphoblastic leukemia; AML, acute myeloid leukemia; BCR-ABL, breakpoint cluster region-Abelson; BTK, Bruton tyrosine kinase; CLL, chronic lymphocytic leukemia; CML, chronic myeloid leukemia; CRC, colorectal cancer; CSF-1R, colony stimulating factor 1 receptor; DDR, discoidin domain receptor; EGFR, epidermal growth factor receptor; ErbB, erythroblastic leukemia viral oncogene homolog; FGFR, fibroblast growth factor receptor; FLT, Fms-like tyrosine kinase; GIST, gastrointestinal stromal tumor; GVHD, graft-versus-host disease; HCC, hepatocellular carcinoma; HER, human epidermal growth factor receptor; IRAK1, interleukin-1 receptor-associated kinase 1; ITK, interleukin-2-inducible T-cell kinase; JAK, Janus kinase; KIT, mast/stem cell growth factor receptor; MEK, mitogen-activated protein kinase kinase; MET, mesenchymal-epithelial transition factor; NSCLC, non-small cell lung cancer; NTRK, neurotrophic tyrosine receptor kinase; PDGFR, platelet-derived growth factor receptor; Ph, Philadelphia chromosome (BCR-ABL); PKC, protein kinase C; PNET, pancreatic neuroendocrine tumor; RAF, rapidly accelerated fibrosarcoma; RCC, renal cell carcinoma; RET, rearranged during transfection; SLL, small lymphocytic lymphoma; SRC, sarcoma proto-oncogene tyrosine-protein kinase; STAMP, specifically targeting the ABL myristoyl pocket; SYK, spleen tyrosine kinase; TIE, tyrosine kinase with immunoglobulin-like and EGF-like domains 2; TRK, tropomyosin receptor kinase; TYK, tyrosine kinase; VEGFR, vascular endothelial growth factor receptor.
