# Supplementary material for: Risks of Retinal Vascular Occlusions with the Systemic Use of Tyrosine Kinase Inhibitors
Source: Ophthalmol Retina. Author manuscript; Available in PMC 2026 Sep 21. (PMC13591267; doi:10.1016/j.oret.2026.02.019)
Supplement: Supplementary file 2 [file NIHMS2194774-supplement-10.pdf]

eTable 3I: Results of the Propensity Score Matching Analysis Performed for the Risk Ratio Analysis Comparing the BCR-ABL TKI (Study) Cohort to the Non-TKI (Control) Cohort

	Before Matching					After Matching				
	Study		Control		SMD	Study		Control		SMD
Number of Patients	3,706		295,389			3,704		3,704		
Age at Index	61.87	(16.39)	68.17	(13.34)	0.422	61.90	(16.35)	62.17	(16.66)	0.016
Female	1,836	(49.54%)	168,335	(56.99%)	0.150	1,835	(49.54%)	1,903	(51.38%)	0.037
Ethnicity										
Hispanic or Latino	362	(9.77%)	15,202	(5.15%)	0.177	361	(9.75%)	313	(8.45%)	0.045
Unknown	632	(17.05%)	52,270	(17.70%)	0.017	632	(17.06%)	617	(16.66%)	0.011
Race										
White	2,479	(66.89%)	231,475	(78.36%)	0.259	2,479	(66.93%)	2,532	(68.36%)	0.031
Black or African American	629	(16.97%)	34,336	(11.62%)	0.153	628	(16.95%)	606	(16.36%)	0.016
Asian	165	(4.45%)	8,914	(3.02%)	0.076	165	(4.45%)	170	(4.59%)	0.006
Other	137	(3.70%)	6,324	(2.14%)	0.093	137	(3.70%)	113	(3.05%)	0.036
Unknown	252	(6.80%)	12,161	(4.12%)	0.118	251	(6.78%)	234	(6.32%)	0.019
[a]*Body Mass Index (kg/m²) [mean (SD)]*	28.99	(7.26)	28.53	(6.61)	0.065	29.00	(7.25)	29.27	(7.29)	0.037
<18.5	311	(8.39%)	21,321	(7.22%)	0.044	309	(8.34%)	285	(7.69%)	0.024
18.5-24.9	1,271	(34.30%)	108,616	(36.77%)	0.052	1,270	(34.29%)	1,239	(33.45%)	0.018
25-29.9	1,669	(45.04%)	142,151	(48.12%)	0.062	1,668	(45.03%)	1,686	(45.52%)	0.010
30-40	1,443	(38.94%)	116,300	(39.37%)	0.009	1,442	(38.93%)	1,418	(38.28%)	0.013
>40.0	463	(12.49%)	32,750	(11.09%)	0.044	463	(12.50%)	469	(12.66%)	0.005
Persons with potential health hazards related to socioeconomic and psychosocial circumstances	261	(7.04%)	19,403	(6.57%)	0.019	261	(7.05%)	252	(6.80%)	0.010
Tobacco use	185	(4.99%)	18,456	(6.25%)	0.055	185	(4.99%)	186	(5.02%)	0.001
Nicotine dependence	494	(13.33%)	46,758	(15.83%)	0.071	494	(13.34%)	462	(12.47%)	0.026
Alcohol related disorders	168	(4.53%)	18,218	(6.17%)	0.073	168	(4.54%)	142	(3.83%)	0.035
Diseases of the circulatory system	3,083	(83.19%)	252,478	(85.47%)	0.063	3,082	(83.21%)	3,051	(82.37%)	0.022
Hypertensive diseases	2,491	(67.22%)	203,731	(68.97%)	0.038	2,490	(67.22%)	2,458	(66.36%)	0.018
Ischemic heart diseases	1,057	(28.52%)	84,659	(28.66%)	0.003	1,057	(28.54%)	1,020	(27.54%)	0.022
Heart failure	654	(17.65%)	42,325	(14.33%)	0.091	654	(17.66%)	647	(17.47%)	0.005
Diseases of arteries, arterioles and capillaries	867	(23.39%)	89,315	(30.24%)	0.155	867	(23.41%)	855	(23.08%)	0.008
Diabetes mellitus	1,350	(36.43%)	93,197	(31.55%)	0.103	1,349	(36.42%)	1,346	(36.34%)	0.002
Cerebrovascular diseases	664	(17.92%)	54,816	(18.56%)	0.017	664	(17.93%)	662	(17.87%)	0.001
Chronic kidney disease (CKD)	816	(22.02%)	54,993	(18.62%)	0.085	815	(22.00%)	836	(22.57%)	0.014
Primary thrombophilia	63	(1.70%)	4,845	(1.64%)	0.005	63	(1.70%)	62	(1.67%)	0.002
Other thrombophilia	62	(1.67%)	4,465	(1.51%)	0.013	62	(1.67%)	58	(1.57%)	0.009
Polycythemia vera	15	(0.40%)	1,958	(0.66%)	0.035	15	(0.40%)	14	(0.38%)	0.004
Homocystinuria	10	(0.27%)	593	(0.20%)	0.014	10	(0.27%)	10	(0.27%)	0.000
Malignant immunoproliferative diseases and certain other B-cell lymphomas	17	(0.46%)	2,085	(0.71%)	0.032	17	(0.46%)	16	(0.43%)	0.004
Multiple myeloma and malignant plasma cell neoplasms	116	(3.13%)	10,959	(3.71%)	0.032	116	(3.13%)	114	(3.08%)	0.003
Systemic lupus erythematosus (SLE)	42	(1.13%)	4,653	(1.58%)	0.038	42	(1.13%)	51	(1.38%)	0.022
Glaucoma	652	(17.59%)	60,206	(20.38%)	0.071	652	(17.60%)	610	(16.47%)	0.030
Medications										
Antithrombotic agents	2,711	(73.15%)	207,443	(70.23%)	0.065	2,710	(73.16%)	2,670	(72.08%)	0.024
Antihypertensives	941	(25.39%)	78,992	(26.74%)	0.031	941	(25.40%)	900	(24.30%)	0.026
Estrogens	315	(8.50%)	35,197	(11.92%)	0.113	315	(8.50%)	323	(8.72%)	0.008
Immunosuppressants	788	(21.26%)	50,996	(17.26%)	0.102	786	(21.22%)	734	(19.82%)	0.035
Other antineoplastics										
Alkylating agents	454	(12.25%)	29,461	(9.97%)	0.072	454	(12.26%)	431	(11.64%)	0.019
Antimetabolites	651	(17.57%)	94,173	(31.88%)	0.336	651	(17.58%)	549	(14.82%)	0.075
Plant alkaloids and other natural products	358	(9.66%)	34,702	(11.75%)	0.068	358	(9.67%)	353	(9.53%)	0.005
Cytotoxic antibiotics and related substances	312	(8.42%)	21,453	(7.26%)	0.043	312	(8.42%)	284	(7.67%)	0.028
Monoclonal antibodies	268	(7.23%)	44,308	(15.00%)	0.249	268	(7.24%)	238	(6.43%)	0.032
Radiation Oncology Treatment	268	(7.23%)	33,257	(11.26%)	0.139	268	(7.24%)	286	(7.72%)	0.018
Cancer stages										
Stage 0	10	(0.27%)	1,853	(0.63%)	0.054	10	(0.27%)	14	(0.38%)	0.019
Stage 1	29	(0.78%)	6,668	(2.26%)	0.121	29	(0.78%)	39	(1.05%)	0.028
Stage 2	27	(0.73%)	4,236	(1.43%)	0.068	27	(0.73%)	27	(0.73%)	0.000
Stage 3	14	(0.38%)	3,206	(1.09%)	0.083	14	(0.38%)	16	(0.43%)	0.009
Stage 4	13	(0.35%)	3,107	(1.05%)	0.084	13	(0.35%)	17	(0.46%)	0.017

Abbreviations: SD, standard deviation; SMD, standardized mean difference; TKI, tyrosine kinase inhibitor

[a]*Data indexed at baseline was used*
