# Supplementary material for: Risks of Retinal Vascular Occlusions with the Systemic Use of Tyrosine Kinase Inhibitors
Source: Ophthalmol Retina. Author manuscript; Available in PMC 2026 Sep 21. (PMC13591267; doi:10.1016/j.oret.2026.02.019)
Supplement: Supplementary file 3 [file NIHMS2194774-supplement-11.pdf]

eTable 3J: Results of the Propensity Score Matching Analysis Performed for the Hazard Ratio Analysis Comparing the BCR-ABL TKI (Study) Cohort to the Non-TKI (Control) Cohort

	Before Matching					After Matching				
	Study		Control		SMD	Study		Control		SMD
Number of Patients	3,771		302,823			3,770		3,770		
Age at Index	61.91	(16.29)	68.18	(13.34)	0.421	61.92	(16.27)	61.98	(17.09)	0.003
Female	1,854	(49.16%)	173,242	(57.21%)	0.162	1,854	(49.18%)	1,872	(49.66%)	0.010
Ethnicity										
Hispanic or Latino	349	(9.25%)	14,661	(4.84%)	0.173	349	(9.26%)	322	(8.54%)	0.025
Unknown	609	(16.15%)	50,588	(16.71%)	0.015	609	(16.15%)	579	(15.36%)	0.022
Race										
White	2,587	(68.60%)	241,101	(79.62%)	0.253	2,587	(68.62%)	2,577	(68.36%)	0.006
Black or African American	605	(16.04%)	33,031	(10.91%)	0.151	604	(16.02%)	622	(16.50%)	0.013
Asian	159	(4.22%)	8,582	(2.83%)	0.075	159	(4.22%)	158	(4.19%)	0.001
Other	139	(3.69%)	6,195	(2.05%)	0.098	139	(3.69%)	151	(4.01%)	0.017
Unknown	239	(6.34%)	11,813	(3.90%)	0.111	239	(6.34%)	229	(6.07%)	0.011
[a]*Body Mass Index (kg/m²) [mean (SD)]*	29.13	(7.27)	28.67	(6.64)	0.066	29.14	(7.27)	29.05	(6.85)	0.012
<18.5	318	(8.43%)	22,032	(7.28%)	0.043	317	(8.41%)	316	(8.38%)	0.001
18.5-24.9	1,291	(34.23%)	111,466	(36.81%)	0.054	1,290	(34.22%)	1,284	(34.06%)	0.003
25-29.9	1,727	(45.80%)	148,050	(48.89%)	0.062	1,726	(45.78%)	1,715	(45.49%)	0.006
30-40	1,514	(40.15%)	122,893	(40.58%)	0.009	1,513	(40.13%)	1,498	(39.73%)	0.008
>40.0	494	(13.10%)	34,795	(11.49%)	0.049	494	(13.10%)	458	(12.15%)	0.029
Persons with potential health hazards related to socioeconomic and psychosocial circumstances	266	(7.05%)	20,193	(6.67%)	0.015	266	(7.06%)	256	(6.79%)	0.010
Tobacco use	192	(5.09%)	19,378	(6.40%)	0.056	192	(5.09%)	191	(5.07%)	0.001
Nicotine dependence	516	(13.68%)	48,694	(16.08%)	0.067	516	(13.69%)	493	(13.08%)	0.018
Alcohol related disorders	179	(4.75%)	18,500	(6.11%)	0.060	179	(4.75%)	178	(4.72%)	0.001
Diseases of the circulatory system	3,123	(82.82%)	258,384	(85.33%)	0.069	3,122	(82.81%)	3,106	(82.39%)	0.011
Hypertensive diseases	2,529	(67.06%)	209,292	(69.11%)	0.044	2,528	(67.06%)	2,501	(66.34%)	0.015
Ischemic heart diseases	1,069	(28.35%)	87,416	(28.87%)	0.011	1,069	(28.36%)	970	(25.73%)	0.059
Heart failure	639	(16.95%)	42,897	(14.17%)	0.077	639	(16.95%)	590	(15.65%)	0.035
Diseases of arteries, arterioles and capillaries	885	(23.47%)	91,713	(30.29%)	0.154	885	(23.47%)	839	(22.25%)	0.029
Diabetes mellitus	1,374	(36.44%)	95,648	(31.59%)	0.103	1,373	(36.42%)	1,346	(35.70%)	0.015
Cerebrovascular diseases	661	(17.53%)	56,127	(18.53%)	0.026	661	(17.53%)	620	(16.45%)	0.029
Chronic kidney disease (CKD)	824	(21.85%)	56,107	(18.53%)	0.083	823	(21.83%)	768	(20.37%)	0.036
Primary thrombophilia	66	(1.75%)	4,900	(1.62%)	0.010	66	(1.75%)	63	(1.67%)	0.006
Other thrombophilia	61	(1.62%)	4,319	(1.43%)	0.016	61	(1.62%)	53	(1.41%)	0.017
Polycythemia vera	13	(0.34%)	2,024	(0.67%)	0.046	13	(0.34%)	15	(0.40%)	0.009
Homocystinuria	10	(0.27%)	602	(0.20%)	0.014	10	(0.27%)	10	(0.27%)	0.000
Malignant immunoproliferative diseases and certain other B-cell lymphomas	19	(0.50%)	2,168	(0.72%)	0.027	19	(0.50%)	17	(0.45%)	0.008
Multiple myeloma and malignant plasma cell neoplasms	113	(3.00%)	11,028	(3.64%)	0.036	113	(3.00%)	116	(3.08%)	0.005
Systemic lupus erythematosus (SLE)	40	(1.06%)	4,759	(1.57%)	0.045	40	(1.06%)	30	(0.80%)	0.028
Glaucoma	655	(17.37%)	62,066	(20.50%)	0.080	655	(17.37%)	661	(17.53%)	0.004
Medications										
Antithrombotic agents	2,726	(72.29%)	210,405	(69.48%)	0.062	2,725	(72.28%)	2,641	(70.05%)	0.049
Antihypertensives	938	(24.87%)	80,102	(26.45%)	0.036	938	(24.88%)	868	(23.02%)	0.044
Estrogens	323	(8.57%)	36,702	(12.12%)	0.117	323	(8.57%)	319	(8.46%)	0.004
Immunosuppressants	772	(20.47%)	52,249	(17.25%)	0.082	771	(20.45%)	731	(19.39%)	0.027
Other antineoplastics										
Alkylating agents	429	(11.38%)	28,969	(9.57%)	0.059	429	(11.38%)	398	(10.56%)	0.026
Antimetabolites	635	(16.84%)	95,681	(31.60%)	0.350	635	(16.84%)	539	(14.30%)	0.070
Plant alkaloids and other natural products	344	(9.12%)	33,721	(11.14%)	0.067	344	(9.12%)	342	(9.07%)	0.002
Cytotoxic antibiotics and related substances	296	(7.85%)	22,241	(7.34%)	0.019	296	(7.85%)	303	(8.04%)	0.007
Monoclonal antibodies	269	(7.13%)	45,679	(15.08%)	0.255	269	(7.14%)	277	(7.35%)	0.008
Radiation Oncology Treatment	252	(6.68%)	33,490	(11.06%)	0.154	252	(6.68%)	252	(6.68%)	0.000
Cancer stages										
Stage 0	10	(0.27%)	2,159	(0.71%)	0.064	10	(0.27%)	11	(0.29%)	0.005
Stage 1	36	(0.95%)	7,820	(2.58%)	0.124	36	(0.95%)	48	(1.27%)	0.030
Stage 2	29	(0.77%)	4,881	(1.61%)	0.078	29	(0.77%)	36	(0.95%)	0.020
Stage 3	17	(0.45%)	3,590	(1.19%)	0.082	17	(0.45%)	20	(0.53%)	0.011
Stage 4	14	(0.37%)	3,160	(1.04%)	0.080	14	(0.37%)	14	(0.37%)	0.000

Abbreviations: SD, standard deviation; SMD, standardized mean difference; TKI, tyrosine kinase inhibitor

[a]*Data indexed at baseline was used*
