# Supplementary material for: Risks of Retinal Vascular Occlusions with the Systemic Use of Tyrosine Kinase Inhibitors
Source: Ophthalmol Retina. Author manuscript; Available in PMC 2026 Sep 21. (PMC13591267; doi:10.1016/j.oret.2026.02.019)
Supplement: Supplementary file 4 [file NIHMS2194774-supplement-12.pdf]

eFigure 1A: Results of the Sensitivity Analysis Comparing Patients With at Least a 6-Month Prescription Records of TKIs (Study Cohort) Versus Those with Non-TKI Antineoplastic Agents (Control Cohort) on the Risks of Having Retinal Vascular Occlusions

Outcome	Study Events / Total	Control Events / Total	Risk Ratio (95% CI)
Retinal vein occlusion	144 / 13 248	175 / 13 230	0.82 (0.66 – 1.02)
Central retinal vein occlusion	97 / 13 278	89 / 13 272	1.09 (0.82 – 1.45)
Branch retinal vein occlusion	68 / 13 277	114 / 13 260	0.60 (0.44 – 0.80)
Retinal artery occlusion	74 / 13 247	111 / 13 217	0.67 (0.50 – 0.89)
Central retinal artery occlusion	28 / 13 301	34 / 13 294	0.82 (0.50 – 1.36)
Branch retinal artery occlusion	27 / 13 294	40 / 13 285	0.68 (0.41 – 1.10)

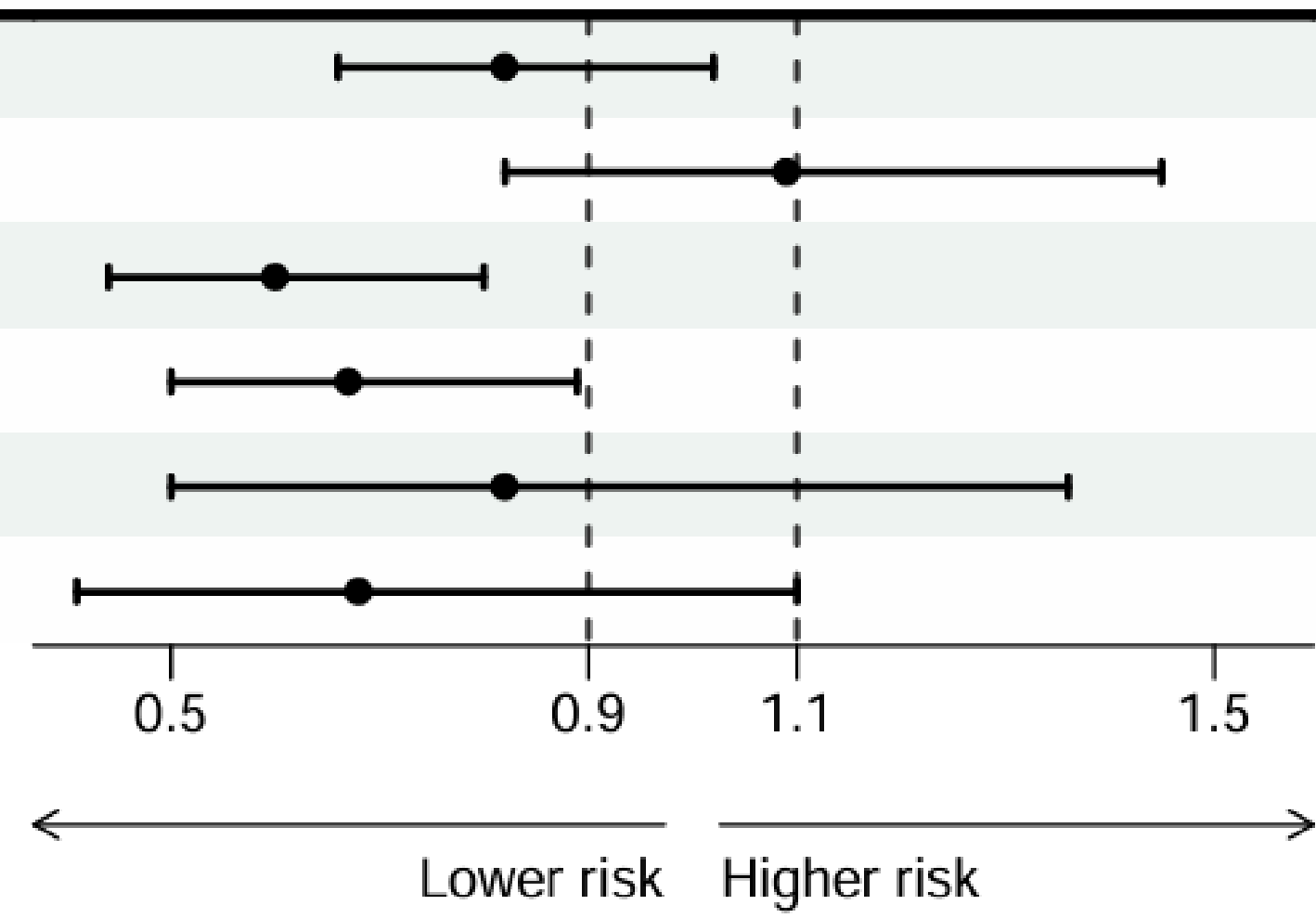

Abbreviation: TKI, tyrosine kinase inhibitor
