# Supplementary material for: Risks of Retinal Vascular Occlusions with the Systemic Use of Tyrosine Kinase Inhibitors
Source: Ophthalmol Retina. Author manuscript; Available in PMC 2026 Sep 21. (PMC13591267; doi:10.1016/j.oret.2026.02.019)
Supplement: Supplementary file 5 [file NIHMS2194774-supplement-13.pdf]

eTable 3K: Results of the Propensity Score Matching Analysis Performed for the Risk Ratio Analysis Comparing the BCR-ABL TKI (Study) Cohort to the Non-BCR-ABL TKI (Control) Cohort

	Before Matching					After Matching				
	Study		Control		SMD	Study		Control		SMD
Number of Patients	3,830		19,787			3,806		3,806		
Age at Index	61.82	(16.40)	66.75	(13.78)	0.325	61.99	(16.29)	61.85	(16.25)	0.009
Female	1,884	(49.19%)	9,544	(48.23%)	0.019	1,869	(49.11%)	1,861	(48.90%)	0.004
Ethnicity										
Hispanic or Latino	366	(9.56%)	1,272	(6.43%)	0.116	361	(9.49%)	366	(9.62%)	0.004
Unknown	632	(16.50%)	3,025	(15.29%)	0.033	629	(16.53%)	624	(16.40%)	0.004
Race										
White	2,590	(67.62%)	14,616	(73.87%)	0.138	2,581	(67.81%)	2,559	(67.24%)	0.012
Black or African American	632	(16.50%)	2,263	(11.44%)	0.146	625	(16.42%)	653	(17.16%)	0.020
Asian	169	(4.41%)	1,232	(6.23%)	0.081	169	(4.44%)	171	(4.49%)	0.003
Other	143	(3.73%)	557	(2.81%)	0.052	141	(3.70%)	141	(3.70%)	0.000
Unknown	252	(6.58%)	975	(4.93%)	0.071	248	(6.52%)	234	(6.15%)	0.015
[a]*Body Mass Index (kg/m²) [mean (SD)]*	28.97	(7.23)	27.36	(6.22)	0.239	28.95	(7.20)	28.51	(7.01)	0.061
<18.5	326	(8.51%)	2,227	(11.25%)	0.092	326	(8.57%)	305	(8.01%)	0.020
18.5-24.9	1,337	(34.91%)	9,016	(45.57%)	0.219	1,330	(34.94%)	1,315	(34.55%)	0.008
25-29.9	1,742	(45.48%)	10,734	(54.25%)	0.176	1,737	(45.64%)	1,724	(45.30%)	0.007
30-40	1,501	(39.19%)	7,888	(39.86%)	0.014	1,494	(39.25%)	1,473	(38.70%)	0.011
>40.0	479	(12.51%)	2,083	(10.53%)	0.062	473	(12.43%)	484	(12.72%)	0.009
Persons with potential health hazards related to socioeconomic and psychosocial circumstances	270	(7.05%)	1,316	(6.65%)	0.016	266	(6.99%)	260	(6.83%)	0.006
Tobacco use	190	(4.96%)	1,040	(5.26%)	0.013	190	(4.99%)	205	(5.39%)	0.018
Nicotine dependence	512	(13.37%)	2,910	(14.71%)	0.039	508	(13.35%)	534	(14.03%)	0.020
Alcohol related disorders	173	(4.52%)	1,216	(6.15%)	0.073	173	(4.55%)	163	(4.28%)	0.013
Diseases of the circulatory system	3,170	(82.77%)	17,598	(88.94%)	0.178	3,160	(83.03%)	3,173	(83.37%)	0.009
Hypertensive diseases	2,564	(66.95%)	13,765	(69.57%)	0.056	2,555	(67.13%)	2,560	(67.26%)	0.003
Ischemic heart diseases	1,075	(28.07%)	6,155	(31.11%)	0.067	1,073	(28.19%)	1,088	(28.59%)	0.009
Heart failure	666	(17.39%)	3,343	(16.89%)	0.013	662	(17.39%)	668	(17.55%)	0.004
Diseases of arteries, arterioles and capillaries	882	(23.03%)	5,921	(29.92%)	0.157	882	(23.17%)	870	(22.86%)	0.007
Diabetes mellitus	1,378	(35.98%)	6,494	(32.82%)	0.067	1,372	(36.05%)	1,402	(36.84%)	0.016
Cerebrovascular diseases	676	(17.65%)	4,228	(21.37%)	0.094	675	(17.74%)	683	(17.95%)	0.005
Chronic kidney disease (CKD)	836	(21.83%)	4,647	(23.49%)	0.040	833	(21.89%)	823	(21.62%)	0.006
Primary thrombophilia	66	(1.72%)	500	(2.53%)	0.056	66	(1.73%)	66	(1.73%)	0.000
Other thrombophilia	63	(1.64%)	619	(3.13%)	0.097	63	(1.66%)	57	(1.50%)	0.013
Polycythemia vera	15	(0.39%)	547	(2.76%)	0.191	15	(0.39%)	20	(0.53%)	0.019
Homocystinuria	10	(0.26%)	37	(0.19%)	0.016	10	(0.26%)	10	(0.26%)	0.000
Malignant immunoproliferative diseases and certain other B-cell lymphomas	20	(0.52%)	806	(4.07%)	0.239	20	(0.53%)	26	(0.68%)	0.020
Multiple myeloma and malignant plasma cell neoplasms	117	(3.05%)	713	(3.60%)	0.031	117	(3.07%)	139	(3.65%)	0.032
Systemic lupus erythematosus (SLE)	43	(1.12%)	236	(1.19%)	0.007	43	(1.13%)	41	(1.08%)	0.005
Glaucoma	663	(17.31%)	3,560	(17.99%)	0.018	658	(17.29%)	643	(16.89%)	0.010
Medications										
Antithrombotic agents	2,785	(72.72%)	15,852	(80.11%)	0.175	2,773	(72.86%)	2,822	(74.15%)	0.029
Antihypertensives	967	(25.25%)	5,540	(28.00%)	0.062	965	(25.35%)	968	(25.43%)	0.002
Estrogens	321	(8.38%)	1,861	(9.41%)	0.036	321	(8.43%)	310	(8.15%)	0.010
Immunosuppressants	815	(21.28%)	5,133	(25.94%)	0.110	814	(21.39%)	829	(21.78%)	0.010
Other antineoplastics										
Alkylating agents	471	(12.30%)	3,346	(16.91%)	0.131	470	(12.35%)	496	(13.03%)	0.021
Antimetabolites	685	(17.89%)	5,718	(28.90%)	0.262	684	(17.97%)	704	(18.50%)	0.014
Plant alkaloids and other natural products	377	(9.84%)	2,586	(13.07%)	0.101	376	(9.88%)	384	(10.09%)	0.007
Cytotoxic antibiotics and related substances	325	(8.49%)	2,099	(10.61%)	0.072	324	(8.51%)	329	(8.64%)	0.005
Monoclonal antibodies	284	(7.42%)	5,346	(27.02%)	0.538	284	(7.46%)	280	(7.36%)	0.004
Radiation Oncology Treatment	280	(7.31%)	4,376	(22.12%)	0.427	280	(7.36%)	288	(7.57%)	0.008
Cancer stages										
Stage 0	10	(0.26%)	63	(0.32%)	0.011	10	(0.26%)	10	(0.26%)	0.000
Stage 1	36	(0.94%)	432	(2.18%)	0.100	36	(0.95%)	31	(0.81%)	0.014
Stage 2	30	(0.78%)	303	(1.53%)	0.070	30	(0.79%)	22	(0.58%)	0.026
Stage 3	17	(0.44%)	395	(2.00%)	0.142	17	(0.45%)	20	(0.53%)	0.011
Stage 4	16	(0.42%)	679	(3.43%)	0.221	16	(0.42%)	24	(0.63%)	0.029

Abbreviations: SD, standard deviation; SMD, standardized mean difference; TKI, tyrosine kinase inhibitor

[a]*Data indexed at baseline was used*
