# Supplementary material for: Risks of Retinal Vascular Occlusions with the Systemic Use of Tyrosine Kinase Inhibitors
Source: Ophthalmol Retina. Author manuscript; Available in PMC 2026 Sep 21. (PMC13591267; doi:10.1016/j.oret.2026.02.019)
Supplement: Supplementary file 6 [file NIHMS2194774-supplement-14.pdf]

eTable 3L: Results of the Propensity Score Matching Analysis Performed for the Hazard Ratio Analysis Comparing the BCR-ABL TKI (Study) Cohort to the Non-BCR-ABL TKI (Control) Cohort

	Before Matching					After Matching				
	Study		Control		SMD	Study		Control		SMD
Number of Patients	3,771		18,112			3,700		3,700		
Age at Index	61.91	(16.29)	67.13	(13.66)	0.348	62.47	(15.85)	62.27	(16.05)	0.012
Female	1,854	(49.16%)	8,788	(48.52%)	0.013	1,822	(49.24%)	1,817	(49.11%)	0.003
Ethnicity										
Hispanic or Latino	349	(9.25%)	1,118	(6.17%)	0.116	331	(8.95%)	330	(8.92%)	0.001
Unknown	609	(16.15%)	2,754	(15.21%)	0.026	603	(16.30%)	633	(17.11%)	0.022
Race										
White	2,587	(68.60%)	13,483	(74.44%)	0.130	2,554	(69.03%)	2,519	(68.08%)	0.020
Black or African American	605	(16.04%)	2,034	(11.23%)	0.141	588	(15.89%)	593	(16.03%)	0.004
Asian	159	(4.22%)	1,112	(6.14%)	0.087	158	(4.27%)	167	(4.51%)	0.012
Other	139	(3.69%)	480	(2.65%)	0.059	131	(3.54%)	136	(3.68%)	0.007
Unknown	239	(6.34%)	877	(4.84%)	0.065	230	(6.22%)	245	(6.62%)	0.017
[a]*Body Mass Index (kg/m²) [mean (SD)]*	29.13	(7.27)	27.44	(6.20)	0.250	29.08	(7.18)	28.75	(7.02)	0.047
<18.5	318	(8.43%)	2,003	(11.06%)	0.089	309	(8.35%)	313	(8.46%)	0.004
18.5-24.9	1,291	(34.23%)	8,288	(45.76%)	0.237	1,273	(34.41%)	1,253	(33.86%)	0.011
25-29.9	1,727	(45.80%)	9,978	(55.09%)	0.187	1,704	(46.05%)	1,705	(46.08%)	0.001
30-40	1,514	(40.15%)	7,382	(40.76%)	0.012	1,491	(40.30%)	1,516	(40.97%)	0.014
>40.0	494	(13.10%)	1,948	(10.76%)	0.072	478	(12.92%)	498	(13.46%)	0.016
Persons with potential health hazards related to socioeconomic and psychosocial circumstances	266	(7.05%)	1,255	(6.93%)	0.005	258	(6.97%)	238	(6.43%)	0.022
Tobacco use	192	(5.09%)	1,007	(5.56%)	0.021	189	(5.11%)	179	(4.84%)	0.012
Nicotine dependence	516	(13.68%)	2,700	(14.91%)	0.035	507	(13.70%)	489	(13.22%)	0.014
Alcohol related disorders	179	(4.75%)	1,100	(6.07%)	0.059	179	(4.84%)	168	(4.54%)	0.014
Diseases of the circulatory system	3,123	(82.82%)	16,156	(89.20%)	0.185	3,084	(83.35%)	3,089	(83.49%)	0.004
Hypertensive diseases	2,529	(67.06%)	12,707	(70.16%)	0.067	2,506	(67.73%)	2,526	(68.27%)	0.012
Ischemic heart diseases	1,069	(28.35%)	5,810	(32.08%)	0.081	1,064	(28.76%)	1,050	(28.38%)	0.008
Heart failure	639	(16.95%)	3,096	(17.09%)	0.004	630	(17.03%)	642	(17.35%)	0.009
Diseases of arteries, arterioles and capillaries	885	(23.47%)	5,669	(31.30%)	0.176	882	(23.84%)	856	(23.14%)	0.017
Diabetes mellitus	1,374	(36.44%)	6,008	(33.17%)	0.069	1,355	(36.62%)	1,356	(36.65%)	0.001
Cerebrovascular diseases	661	(17.53%)	3,914	(21.61%)	0.103	650	(17.57%)	639	(17.27%)	0.008
Chronic kidney disease (CKD)	824	(21.85%)	4,336	(23.94%)	0.050	813	(21.97%)	843	(22.78%)	0.019
Primary thrombophilia	66	(1.75%)	460	(2.54%)	0.055	64	(1.73%)	76	(2.05%)	0.024
Other thrombophilia	61	(1.62%)	557	(3.08%)	0.096	60	(1.62%)	60	(1.62%)	0.000
Polycythemia vera	13	(0.34%)	541	(2.99%)	0.208	13	(0.35%)	20	(0.54%)	0.028
Homocystinuria	10	(0.27%)	38	(0.21%)	0.011	10	(0.27%)	10	(0.27%)	0.000
Malignant immunoproliferative diseases and certain other B-cell lymphomas	19	(0.50%)	801	(4.42%)	0.255	19	(0.51%)	32	(0.86%)	0.042
Multiple myeloma and malignant plasma cell neoplasms	113	(3.00%)	671	(3.70%)	0.039	111	(3.00%)	123	(3.32%)	0.019
Systemic lupus erythematosus (SLE)	40	(1.06%)	221	(1.22%)	0.015	40	(1.08%)	46	(1.24%)	0.015
Glaucoma	655	(17.37%)	3,353	(18.51%)	0.030	649	(17.54%)	630	(17.03%)	0.014
Medications										
Antithrombotic agents	2,726	(72.29%)	14,496	(80.04%)	0.183	2,685	(72.57%)	2,656	(71.78%)	0.017
Antihypertensives	938	(24.87%)	5,129	(28.32%)	0.078	927	(25.05%)	923	(24.95%)	0.002
Estrogens	323	(8.57%)	1,772	(9.78%)	0.042	318	(8.59%)	303	(8.19%)	0.015
Immunosuppressants	772	(20.47%)	4,698	(25.94%)	0.130	762	(20.59%)	784	(21.19%)	0.015
Other antineoplastics										
Alkylating agents	429	(11.38%)	3,017	(16.66%)	0.153	425	(11.49%)	429	(11.59%)	0.003
Antimetabolites	635	(16.84%)	5,107	(28.20%)	0.274	629	(17.00%)	609	(16.46%)	0.014
Plant alkaloids and other natural products	344	(9.12%)	2,321	(12.81%)	0.118	338	(9.14%)	336	(9.08%)	0.002
Cytotoxic antibiotics and related substances	296	(7.85%)	1,851	(10.22%)	0.083	291	(7.86%)	286	(7.73%)	0.005
Monoclonal antibodies	269	(7.13%)	4,942	(27.29%)	0.554	269	(7.27%)	256	(6.92%)	0.014
Radiation Oncology Treatment	252	(6.68%)	3,883	(21.44%)	0.434	252	(6.81%)	253	(6.84%)	0.001
Cancer stages										
Stage 0	10	(0.27%)	62	(0.34%)	0.014	10	(0.27%)	10	(0.27%)	0.000
Stage 1	36	(0.95%)	412	(2.27%)	0.105	36	(0.97%)	37	(1.00%)	0.003
Stage 2	29	(0.77%)	281	(1.55%)	0.073	29	(0.78%)	28	(0.76%)	0.003
Stage 3	17	(0.45%)	361	(1.99%)	0.141	17	(0.46%)	25	(0.68%)	0.029
Stage 4	14	(0.37%)	561	(3.10%)	0.210	14	(0.38%)	18	(0.49%)	0.016

Abbreviations: SD, standard deviation; SMD, standardized mean difference; TKI, tyrosine kinase inhibitor

[a]*Data indexed at baseline was used*
