# Supplementary material for: Risks of Retinal Vascular Occlusions with the Systemic Use of Tyrosine Kinase Inhibitors
Source: Ophthalmol Retina. Author manuscript; Available in PMC 2026 Sep 21. (PMC13591267; doi:10.1016/j.oret.2026.02.019)
Supplement: Supplementary file 7 [file NIHMS2194774-supplement-15.pdf]

eTable 3M: Results of the Propensity Score Matching Analysis Performed for the Risk Ratio Analysis Comparing the 6-Month TKI (Study) Cohort to the Non-TKI (Control) Cohort

	Before Matching					After Matching				
	Study		Control		SMD	Study		Control		SMD
Number of Patients	13,318		161,912			13,312		13,312		
Age at Index	63.56	(14.50)	63.80	(13.42)	0.017	63.55	(14.50)	63.20	(14.38)	0.025
Female	6,303	(47.33%)	92,772	(57.30%)	0.201	6,303	(47.35%)	6,327	(47.53%)	0.004
Ethnicity										
Hispanic or Latino	920	(6.91%)	7,546	(4.66%)	0.096	920	(6.91%)	918	(6.90%)	0.001
Unknown	2,154	(16.17%)	26,994	(16.67%)	0.013	2,152	(16.17%)	2,185	(16.41%)	0.007
Race										
White	9,717	(72.96%)	128,147	(79.15%)	0.145	9,712	(72.96%)	9,731	(73.10%)	0.003
Black or African American	1,399	(10.50%)	14,624	(9.03%)	0.050	1,399	(10.51%)	1,397	(10.49%)	0.000
Asian	740	(5.56%)	4,420	(2.73%)	0.142	739	(5.55%)	727	(5.46%)	0.004
Other	479	(3.60%)	4,180	(2.58%)	0.059	479	(3.60%)	473	(3.55%)	0.002
Unknown	898	(6.74%)	9,537	(5.89%)	0.035	898	(6.75%)	914	(6.87%)	0.005
[a]*Body Mass Index (kg/m²) [mean (SD)]*	28.02	(6.50)	28.81	(6.59)	0.120	28.02	(6.50)	28.37	(6.49)	0.053
<18.5	833	(6.25%)	6,796	(4.20%)	0.093	832	(6.25%)	764	(5.74%)	0.022
18.5-24.9	4,738	(35.58%)	49,025	(30.28%)	0.113	4,734	(35.56%)	4,590	(34.48%)	0.023
25-29.9	5,885	(44.19%)	65,399	(40.39%)	0.077	5,880	(44.17%)	5,686	(42.71%)	0.029
30-40	4,523	(33.96%)	54,682	(33.77%)	0.004	4,521	(33.96%)	4,445	(33.39%)	0.012
>40.0	1,198	(9.00%)	14,128	(8.73%)	0.009	1,198	(9.00%)	1,156	(8.68%)	0.011
Persons with potential health hazards related to socioeconomic and psychosocial circumstances	477	(3.58%)	4,820	(2.98%)	0.034	476	(3.58%)	469	(3.52%)	0.003
Tobacco use	409	(3.07%)	5,312	(3.28%)	0.012	409	(3.07%)	369	(2.77%)	0.018
Nicotine dependence	1,587	(11.92%)	20,434	(12.62%)	0.021	1,587	(11.92%)	1,496	(11.24%)	0.021
Alcohol related disorders	541	(4.06%)	6,682	(4.13%)	0.003	541	(4.06%)	506	(3.80%)	0.014
Diseases of the circulatory system	10,100	(75.84%)	114,048	(70.44%)	0.122	10,094	(75.83%)	9,840	(73.92%)	0.044
Hypertensive diseases	7,466	(56.06%)	86,887	(53.66%)	0.048	7,463	(56.06%)	7,203	(54.11%)	0.039
Ischemic heart diseases	2,907	(21.83%)	28,511	(17.61%)	0.106	2,906	(21.83%)	2,757	(20.71%)	0.027
Heart failure	1,331	(9.99%)	11,127	(6.87%)	0.113	1,330	(9.99%)	1,229	(9.23%)	0.026
Diseases of arteries, arterioles and capillaries	2,658	(19.96%)	29,375	(18.14%)	0.046	2,655	(19.94%)	2,514	(18.89%)	0.027
Diabetes mellitus	3,265	(24.52%)	34,117	(21.07%)	0.082	3,263	(24.51%)	3,186	(23.93%)	0.014
Cerebrovascular diseases	1,818	(13.65%)	17,320	(10.70%)	0.090	1,815	(13.63%)	1,703	(12.79%)	0.025
Chronic kidney disease (CKD)	2,106	(15.81%)	15,935	(9.84%)	0.179	2,102	(15.79%)	1,974	(14.83%)	0.027
Primary thrombophilia	213	(1.60%)	1,832	(1.13%)	0.040	211	(1.59%)	212	(1.59%)	0.001
Other thrombophilia	249	(1.87%)	1,573	(0.97%)	0.076	247	(1.86%)	220	(1.65%)	0.015
Polycythemia vera	372	(2.79%)	1,108	(0.68%)	0.162	370	(2.78%)	313	(2.35%)	0.027
Homocystinuria	21	(0.16%)	282	(0.17%)	0.004	21	(0.16%)	24	(0.18%)	0.005
Malignant immunoproliferative diseases and certain other B-cell lymphomas	437	(3.28%)	798	(0.49%)	0.206	432	(3.25%)	324	(2.43%)	0.049
Multiple myeloma and malignant plasma cell neoplasms	341	(2.56%)	5,990	(3.70%)	0.065	341	(2.56%)	335	(2.52%)	0.003
Systemic lupus erythematosus (SLE)	122	(0.92%)	2,224	(1.37%)	0.043	122	(0.92%)	121	(0.91%)	0.001
Glaucoma	1,492	(11.20%)	18,634	(11.51%)	0.010	1,491	(11.20%)	1,376	(10.34%)	0.028
Medications										
Antithrombotic agents	8,718	(65.46%)	90,006	(55.59%)	0.203	8,712	(65.44%)	8,494	(63.81%)	0.034
Antihypertensives	2,487	(18.67%)	24,922	(15.39%)	0.087	2,485	(18.67%)	2,413	(18.13%)	0.014
Estrogens	1,167	(8.76%)	19,686	(12.16%)	0.111	1,167	(8.77%)	1,150	(8.64%)	0.005
Immunosuppressants	2,313	(17.37%)	27,825	(17.19%)	0.005	2,312	(17.37%)	2,162	(16.24%)	0.030
Other antineoplastics										
Alkylating agents	1,416	(10.63%)	11,272	(6.96%)	0.130	1,415	(10.63%)	1,422	(10.68%)	0.002
Antimetabolites	2,442	(18.34%)	52,141	(32.20%)	0.323	2,442	(18.34%)	1,851	(13.90%)	0.121
Plant alkaloids and other natural products	953	(7.16%)	16,190	(10.00%)	0.102	953	(7.16%)	894	(6.72%)	0.017
Cytotoxic antibiotics and related substances	930	(6.98%)	8,778	(5.42%)	0.065	929	(6.98%)	1,015	(7.62%)	0.025
Monoclonal antibodies	1,871	(14.05%)	16,004	(9.88%)	0.129	1,868	(14.03%)	1,810	(13.60%)	0.013
Radiation Oncology Treatment	1,701	(12.77%)	11,614	(7.17%)	0.188	1,701	(12.78%)	1,659	(12.46%)	0.010
Cancer stages										
Stage 0	26	(0.20%)	869	(0.54%)	0.057	26	(0.20%)	41	(0.31%)	0.022
Stage 1	220	(1.65%)	2,726	(1.68%)	0.002	220	(1.65%)	211	(1.59%)	0.005
Stage 2	152	(1.14%)	2,129	(1.31%)	0.016	152	(1.14%)	152	(1.14%)	0.000
Stage 3	190	(1.43%)	1,849	(1.14%)	0.025	190	(1.43%)	147	(1.10%)	0.029
Stage 4	342	(2.57%)	1,742	(1.08%)	0.112	342	(2.57%)	296	(2.22%)	0.023

Abbreviations: SD, standard deviation; SMD, standardized mean difference; TKI, tyrosine kinase inhibitor

[a]*Data indexed at baseline was used*
