# Supplementary material for: Risks of Retinal Vascular Occlusions with the Systemic Use of Tyrosine Kinase Inhibitors
Source: Ophthalmol Retina. Author manuscript; Available in PMC 2026 Sep 21. (PMC13591267; doi:10.1016/j.oret.2026.02.019)
Supplement: Supplementary file 8 [file NIHMS2194774-supplement-16.pdf]

eTable 3N: Results of the Propensity Score Matching Analysis Performed for the Hazard Ratio Analysis Comparing the 6-Month TKI (Study) Cohort to the Non-TKI (Control) Cohort

	Before Matching					After Matching				
	Study		Control		SMD	Study		Control		SMD
Number of Patients	9,911		144,742			9,902		9,902		
Age at Index	63.04	(14.76)	63.50	(13.58)	0.033	63.03	(14.76)	62.57	(14.63)	0.031
Female	4,767	(48.10%)	84,744	(58.55%)	0.211	4,764	(48.11%)	4,670	(47.16%)	0.019
Ethnicity										
Hispanic or Latino	605	(6.10%)	6,023	(4.16%)	0.088	605	(6.11%)	602	(6.08%)	0.001
Unknown	1,722	(17.37%)	24,648	(17.03%)	0.009	1,722	(17.39%)	1,672	(16.89%)	0.013
Race										
White	7,129	(71.93%)	113,940	(78.72%)	0.158	7,123	(71.93%)	7,149	(72.20%)	0.006
Black or African American	1,191	(12.02%)	14,391	(9.94%)	0.066	1,190	(12.02%)	1,165	(11.77%)	0.008
Asian	548	(5.53%)	3,879	(2.68%)	0.144	546	(5.51%)	539	(5.44%)	0.003
Other	280	(2.83%)	2,940	(2.03%)	0.052	280	(2.83%)	277	(2.80%)	0.002
Unknown	702	(7.08%)	8,707	(6.02%)	0.043	702	(7.09%)	702	(7.09%)	0.000
[a]*Body Mass Index (kg/m²) [mean (SD)]*	28.28	(6.58)	29.04	(6.66)	0.114	28.29	(6.58)	28.57	(6.52)	0.043
<18.5	564	(5.69%)	5,526	(3.82%)	0.088	562	(5.68%)	535	(5.40%)	0.012
18.5-24.9	3,168	(31.96%)	39,946	(27.60%)	0.096	3,159	(31.90%)	3,070	(31.00%)	0.019
25-29.9	3,993	(40.29%)	54,525	(37.67%)	0.054	3,987	(40.26%)	3,880	(39.18%)	0.022
30-40	3,178	(32.07%)	46,750	(32.30%)	0.005	3,176	(32.07%)	3,083	(31.14%)	0.020
>40.0	859	(8.67%)	12,461	(8.61%)	0.002	859	(8.68%)	823	(8.31%)	0.013
Persons with potential health hazards related to socioeconomic and psychosocial circumstances	371	(3.74%)	4,519	(3.12%)	0.034	371	(3.75%)	340	(3.43%)	0.017
Tobacco use	334	(3.37%)	4,765	(3.29%)	0.004	334	(3.37%)	295	(2.98%)	0.022
Nicotine dependence	1,152	(11.62%)	17,788	(12.29%)	0.021	1,152	(11.63%)	1,093	(11.04%)	0.019
Alcohol related disorders	378	(3.81%)	5,904	(4.08%)	0.014	378	(3.82%)	361	(3.65%)	0.009
Diseases of the circulatory system	7,427	(74.94%)	102,084	(70.53%)	0.099	7,419	(74.92%)	7,197	(72.68%)	0.051
Hypertensive diseases	5,454	(55.03%)	77,692	(53.68%)	0.027	5,450	(55.04%)	5,279	(53.31%)	0.035
Ischemic heart diseases	2,073	(20.92%)	24,894	(17.20%)	0.095	2,068	(20.88%)	1,977	(19.97%)	0.023
Heart failure	931	(9.39%)	9,540	(6.59%)	0.103	930	(9.39%)	941	(9.50%)	0.004
Diseases of arteries, arterioles and capillaries	1,885	(19.02%)	26,169	(18.08%)	0.024	1,881	(19.00%)	1,791	(18.09%)	0.023
Diabetes mellitus	2,377	(23.98%)	30,167	(20.84%)	0.075	2,376	(24.00%)	2,290	(23.13%)	0.020
Cerebrovascular diseases	1,296	(13.08%)	15,000	(10.36%)	0.084	1,292	(13.05%)	1,231	(12.43%)	0.018
Chronic kidney disease (CKD)	1,401	(14.14%)	13,405	(9.26%)	0.152	1,398	(14.12%)	1,336	(13.49%)	0.018
Primary thrombophilia	140	(1.41%)	1,554	(1.07%)	0.031	140	(1.41%)	113	(1.14%)	0.024
Other thrombophilia	166	(1.67%)	1,318	(0.91%)	0.068	164	(1.66%)	135	(1.36%)	0.024
Polycythemia vera	283	(2.86%)	984	(0.68%)	0.166	280	(2.83%)	230	(2.32%)	0.032
Homocystinuria	13	(0.13%)	245	(0.17%)	0.010	12	(0.12%)	17	(0.17%)	0.013
Malignant immunoproliferative diseases and certain other B-cell lymphomas	354	(3.57%)	730	(0.50%)	0.218	345	(3.48%)	252	(2.54%)	0.055
Multiple myeloma and malignant plasma cell neoplasms	258	(2.60%)	5,291	(3.66%)	0.060	258	(2.61%)	224	(2.26%)	0.022
Systemic lupus erythematosus (SLE)	93	(0.94%)	2,050	(1.42%)	0.044	93	(0.94%)	67	(0.68%)	0.029
Glaucoma	1,113	(11.23%)	16,841	(11.64%)	0.013	1,111	(11.22%)	1,004	(10.14%)	0.035
Medications										
Antithrombotic agents	6,367	(64.24%)	78,504	(54.24%)	0.205	6,361	(64.24%)	6,157	(62.18%)	0.043
Antihypertensives	1,712	(17.27%)	21,265	(14.69%)	0.071	1,712	(17.29%)	1,633	(16.49%)	0.021
Estrogens	864	(8.72%)	17,674	(12.21%)	0.114	864	(8.73%)	791	(7.99%)	0.027
Immunosuppressants	1,730	(17.46%)	25,459	(17.59%)	0.004	1,729	(17.46%)	1,612	(16.28%)	0.032
Other antineoplastics										
Alkylating agents	1,108	(11.18%)	10,010	(6.92%)	0.149	1,105	(11.16%)	1,031	(10.41%)	0.024
Antimetabolites	1,715	(17.30%)	45,752	(31.61%)	0.338	1,715	(17.32%)	1,345	(13.58%)	0.104
Plant alkaloids and other natural products	665	(6.71%)	12,886	(8.90%)	0.082	665	(6.72%)	600	(6.06%)	0.027
Cytotoxic antibiotics and related substances	666	(6.72%)	7,868	(5.44%)	0.054	666	(6.73%)	658	(6.65%)	0.003
Monoclonal antibodies	1,371	(13.83%)	14,027	(9.69%)	0.129	1,366	(13.80%)	1,275	(12.88%)	0.027
Radiation Oncology Treatment	1,068	(10.78%)	9,128	(6.31%)	0.160	1,067	(10.78%)	989	(9.99%)	0.026
Cancer stages										
Stage 0	20	(0.20%)	821	(0.57%)	0.059	20	(0.20%)	28	(0.28%)	0.016
Stage 1	157	(1.58%)	2,466	(1.70%)	0.009	157	(1.59%)	180	(1.82%)	0.018
Stage 2	99	(1.00%)	1,863	(1.29%)	0.027	99	(1.00%)	102	(1.03%)	0.003
Stage 3	136	(1.37%)	1,465	(1.01%)	0.033	136	(1.37%)	147	(1.48%)	0.009
Stage 4	204	(2.06%)	1,185	(0.82%)	0.104	201	(2.03%)	164	(1.66%)	0.028

Abbreviations: SD, standard deviation; SMD, standardized mean difference; TKI, tyrosine kinase inhibitor

[a]*Data indexed at baseline was used*
