# Supplementary material for: Risks of Retinal Vascular Occlusions with the Systemic Use of Tyrosine Kinase Inhibitors
Source: Ophthalmol Retina. Author manuscript; Available in PMC 2026 Sep 21. (PMC13591267; doi:10.1016/j.oret.2026.02.019)
Supplement: Supplementary file 9 [file NIHMS2194774-supplement-17.pdf]

eTable 3O: Results of the Propensity Score Matching Analysis Performed for the Risk Ratio Analysis Comparing the 1-Year TKI (Study) Cohort to the Non-TKI (Control) Cohort

	Before Matching					After Matching				
	Study		Control		SMD	Study		Control		SMD
Number of Patients	10,738		138,830			10,735		10,735		
Age at Index	63.42	(14.39)	63.66	(13.26)	0.018	63.41	(14.39)	63.43	(14.03)	0.001
Female	5,049	(47.02%)	80,196	(57.77%)	0.216	5,047	(47.01%)	5,014	(46.71%)	0.006
Ethnicity										
Hispanic or Latino	714	(6.65%)	6,136	(4.42%)	0.098	714	(6.65%)	688	(6.41%)	0.010
Unknown	1,750	(16.30%)	22,906	(16.50%)	0.005	1,750	(16.30%)	1,791	(16.68%)	0.010
Race										
White	7,865	(73.24%)	110,950	(79.92%)	0.158	7,862	(73.24%)	7,926	(73.83%)	0.014
Black or African American	1,133	(10.55%)	12,064	(8.69%)	0.063	1,133	(10.55%)	1,082	(10.08%)	0.016
Asian	599	(5.58%)	3,572	(2.57%)	0.152	599	(5.58%)	591	(5.51%)	0.003
Other	378	(3.52%)	3,402	(2.45%)	0.063	378	(3.52%)	381	(3.55%)	0.002
Unknown	693	(6.45%)	8,010	(5.77%)	0.029	693	(6.46%)	687	(6.40%)	0.002
[a]*Body Mass Index (kg/m²) [mean (SD)]*	28.12	(6.56)	28.85	(6.58)	0.112	28.12	(6.56)	28.42	(6.49)	0.046
<18.5	633	(5.89%)	5,573	(4.01%)	0.087	633	(5.90%)	583	(5.43%)	0.020
18.5-24.9	3,708	(34.53%)	41,154	(29.64%)	0.105	3,706	(34.52%)	3,677	(34.25%)	0.006
25-29.9	4,607	(42.90%)	55,097	(39.69%)	0.065	4,606	(42.91%)	4,506	(41.97%)	0.019
30-40	3,531	(32.88%)	46,187	(33.27%)	0.008	3,529	(32.87%)	3,507	(32.67%)	0.004
>40.0	953	(8.88%)	11,919	(8.59%)	0.010	953	(8.88%)	935	(8.71%)	0.006
Persons with potential health hazards related to socioeconomic and psychosocial circumstances	333	(3.10%)	3,806	(2.74%)	0.021	332	(3.09%)	322	(3.00%)	0.005
Tobacco use	303	(2.82%)	4,086	(2.94%)	0.007	303	(2.82%)	304	(2.83%)	0.001
Nicotine dependence	1,192	(11.10%)	16,640	(11.99%)	0.028	1,192	(11.10%)	1,123	(10.46%)	0.021
Alcohol related disorders	381	(3.55%)	5,463	(3.94%)	0.020	381	(3.55%)	369	(3.44%)	0.006
Diseases of the circulatory system	7,954	(74.07%)	96,400	(69.44%)	0.103	7,951	(74.07%)	7,777	(72.45%)	0.037
Hypertensive diseases	5,838	(54.37%)	73,191	(52.72%)	0.033	5,837	(54.37%)	5,719	(53.27%)	0.022
Ischemic heart diseases	2,223	(20.70%)	23,463	(16.90%)	0.097	2,221	(20.69%)	2,168	(20.20%)	0.012
Heart failure	972	(9.05%)	8,958	(6.45%)	0.097	972	(9.05%)	962	(8.96%)	0.003
Diseases of arteries, arterioles and capillaries	2,002	(18.64%)	24,586	(17.71%)	0.024	2,002	(18.65%)	1,905	(17.75%)	0.023
Diabetes mellitus	2,517	(23.44%)	27,897	(20.09%)	0.081	2,516	(23.44%)	2,437	(22.70%)	0.017
Cerebrovascular diseases	1,392	(12.96%)	14,049	(10.12%)	0.089	1,392	(12.97%)	1,341	(12.49%)	0.014
Chronic kidney disease (CKD)	1,608	(14.97%)	12,906	(9.30%)	0.175	1,605	(14.95%)	1,548	(14.42%)	0.015
Primary thrombophilia	167	(1.56%)	1,535	(1.11%)	0.039	167	(1.56%)	163	(1.52%)	0.003
Other thrombophilia	184	(1.71%)	1,294	(0.93%)	0.068	184	(1.71%)	163	(1.52%)	0.016
Polycythemia vera	326	(3.04%)	983	(0.71%)	0.172	326	(3.04%)	291	(2.71%)	0.020
Homocystinuria	18	(0.17%)	243	(0.18%)	0.002	18	(0.17%)	19	(0.18%)	0.002
Malignant immunoproliferative diseases and certain other B-cell lymphomas	352	(3.28%)	668	(0.48%)	0.207	349	(3.25%)	253	(2.36%)	0.054
Multiple myeloma and malignant plasma cell neoplasms	250	(2.33%)	5,075	(3.66%)	0.078	250	(2.33%)	257	(2.39%)	0.004
Systemic lupus erythematosus (SLE)	91	(0.85%)	1,959	(1.41%)	0.053	91	(0.85%)	90	(0.84%)	0.001
Glaucoma	1,154	(10.75%)	15,504	(11.17%)	0.013	1,154	(10.75%)	1,096	(10.21%)	0.018
Medications										
Antithrombotic agents	6,807	(63.39%)	74,894	(53.95%)	0.193	6,805	(63.39%)	6,642	(61.87%)	0.031
Antihypertensives	1,867	(17.39%)	20,230	(14.57%)	0.077	1,866	(17.38%)	1,851	(17.24%)	0.004
Estrogens	897	(8.35%)	17,461	(12.58%)	0.138	897	(8.36%)	806	(7.51%)	0.031
Immunosuppressants	1,727	(16.08%)	24,696	(17.79%)	0.045	1,727	(16.09%)	1,616	(15.05%)	0.029
Other antineoplastics										
Alkylating agents	1,079	(10.05%)	9,093	(6.55%)	0.127	1,077	(10.03%)	1,104	(10.28%)	0.008
Antimetabolites	1,806	(16.82%)	45,124	(32.50%)	0.370	1,806	(16.82%)	1,437	(13.39%)	0.096
Plant alkaloids and other natural products	646	(6.02%)	11,991	(8.64%)	0.101	646	(6.02%)	630	(5.87%)	0.006
Cytotoxic antibiotics and related substances	656	(6.11%)	7,346	(5.29%)	0.035	656	(6.11%)	674	(6.28%)	0.007
Monoclonal antibodies	1,367	(12.73%)	11,704	(8.43%)	0.140	1,365	(12.72%)	1,307	(12.18%)	0.016
Radiation Oncology Treatment	1,197	(11.15%)	8,919	(6.42%)	0.167	1,197	(11.15%)	1,163	(10.83%)	0.010
Cancer stages										
Stage 0	20	(0.19%)	731	(0.53%)	0.057	20	(0.19%)	30	(0.28%)	0.019
Stage 1	168	(1.56%)	2,222	(1.60%)	0.003	168	(1.56%)	148	(1.38%)	0.015
Stage 2	117	(1.09%)	1,694	(1.22%)	0.012	117	(1.09%)	101	(0.94%)	0.015
Stage 3	155	(1.44%)	1,433	(1.03%)	0.037	155	(1.44%)	178	(1.66%)	0.017
Stage 4	278	(2.59%)	1,324	(0.95%)	0.124	276	(2.57%)	251	(2.34%)	0.015

Abbreviations: SD, standard deviation; SMD, standardized mean difference; TKI, tyrosine kinase inhibitor

[a]*Data indexed at baseline was used*
