# Supplementary material for: Risks of Retinal Vascular Occlusions with the Systemic Use of Tyrosine Kinase Inhibitors
Source: Ophthalmol Retina. Author manuscript; Available in PMC 2026 Sep 21. (PMC13591267; doi:10.1016/j.oret.2026.02.019)
Supplement: Supplementary file 10 [file NIHMS2194774-supplement-18.pdf]

eTable 3P: Results of the Propensity Score Matching Analysis Performed for the Hazard Ratio Analysis Comparing the 1-Year TKI (Study) Cohort to the Non-TKI (Control) Cohort

	Before Matching					After Matching				
	Study		Control		SMD	Study		Control		SMD
Number of Patients	8,365		126,730			8,362		8,362		
Age at Index	62.91	(14.68)	63.44	(13.41)	0.038	62.91	(14.68)	62.62	(14.37)	0.020
Female	4,002	(47.84%)	74,503	(58.79%)	0.221	4,000	(47.84%)	3,934	(47.05%)	0.016
Ethnicity										
Hispanic or Latino	497	(5.94%)	4,981	(3.93%)	0.093	497	(5.94%)	454	(5.43%)	0.022
Unknown	1,483	(17.73%)	21,451	(16.93%)	0.021	1,483	(17.73%)	1,451	(17.35%)	0.010
Race										
White	6,037	(72.17%)	100,594	(79.38%)	0.169	6,036	(72.18%)	6,113	(73.10%)	0.021
Black or African American	1,006	(12.03%)	12,232	(9.65%)	0.076	1,005	(12.02%)	1,021	(12.21%)	0.006
Asian	455	(5.44%)	3,235	(2.55%)	0.148	454	(5.43%)	423	(5.06%)	0.017
Other	237	(2.83%)	2,459	(1.94%)	0.059	237	(2.83%)	222	(2.65%)	0.011
Unknown	578	(6.91%)	7,461	(5.89%)	0.042	578	(6.91%)	527	(6.30%)	0.025
[a]*Body Mass Index (kg/m²) [mean (SD)]*	28.37	(6.63)	29.05	(6.65)	0.102	28.38	(6.63)	28.73	(6.74)	0.054
<18.5	463	(5.53%)	4,701	(3.71%)	0.087	463	(5.54%)	472	(5.64%)	0.005
18.5-24.9	2,594	(31.01%)	34,571	(27.28%)	0.082	2,593	(31.01%)	2,497	(29.86%)	0.025
25-29.9	3,269	(39.08%)	47,281	(37.31%)	0.036	3,269	(39.09%)	3,093	(36.99%)	0.043
30-40	2,614	(31.25%)	40,527	(31.98%)	0.016	2,613	(31.25%)	2,579	(30.84%)	0.009
>40.0	722	(8.63%)	10,776	(8.50%)	0.005	722	(8.63%)	727	(8.69%)	0.002
Persons with potential health hazards related to socioeconomic and psychosocial circumstances	277	(3.31%)	3,656	(2.88%)	0.025	277	(3.31%)	263	(3.15%)	0.009
Tobacco use	261	(3.12%)	3,845	(3.03%)	0.005	261	(3.12%)	264	(3.16%)	0.002
Nicotine dependence	919	(10.99%)	15,072	(11.89%)	0.028	919	(10.99%)	897	(10.73%)	0.008
Alcohol related disorders	298	(3.56%)	4,975	(3.93%)	0.019	298	(3.56%)	302	(3.61%)	0.003
Diseases of the circulatory system	6,148	(73.50%)	88,510	(69.84%)	0.081	6,146	(73.50%)	5,926	(70.87%)	0.059
Hypertensive diseases	4,489	(53.66%)	67,203	(53.03%)	0.013	4,487	(53.66%)	4,280	(51.18%)	0.050
Ischemic heart diseases	1,687	(20.17%)	21,195	(16.72%)	0.089	1,686	(20.16%)	1,617	(19.34%)	0.021
Heart failure	730	(8.73%)	8,015	(6.32%)	0.091	729	(8.72%)	676	(8.08%)	0.023
Diseases of arteries, arterioles and capillaries	1,514	(18.10%)	22,535	(17.78%)	0.008	1,514	(18.11%)	1,435	(17.16%)	0.025
Diabetes mellitus	1,950	(23.31%)	25,469	(20.10%)	0.078	1,949	(23.31%)	1,923	(23.00%)	0.007
Cerebrovascular diseases	1,050	(12.55%)	12,648	(9.98%)	0.081	1,050	(12.56%)	1,001	(11.97%)	0.018
Chronic kidney disease (CKD)	1,143	(13.66%)	11,256	(8.88%)	0.152	1,141	(13.65%)	1,098	(13.13%)	0.015
Primary thrombophilia	117	(1.40%)	1,334	(1.05%)	0.031	117	(1.40%)	108	(1.29%)	0.009
Other thrombophilia	134	(1.60%)	1,120	(0.88%)	0.065	134	(1.60%)	123	(1.47%)	0.011
Polycythemia vera	259	(3.10%)	902	(0.71%)	0.175	258	(3.09%)	220	(2.63%)	0.027
Homocystinuria	10	(0.12%)	215	(0.17%)	0.013	10	(0.12%)	10	(0.12%)	0.000
Malignant immunoproliferative diseases and certain other B-cell lymphomas	294	(3.51%)	624	(0.49%)	0.217	291	(3.48%)	216	(2.58%)	0.052
Multiple myeloma and malignant plasma cell neoplasms	204	(2.44%)	4,611	(3.64%)	0.070	204	(2.44%)	204	(2.44%)	0.000
Systemic lupus erythematosus (SLE)	72	(0.86%)	1,841	(1.45%)	0.055	71	(0.85%)	71	(0.85%)	0.000
Glaucoma	914	(10.93%)	14,393	(11.36%)	0.014	914	(10.93%)	858	(10.26%)	0.022
Medications										
Antithrombotic agents	5,227	(62.49%)	67,101	(52.95%)	0.194	5,225	(62.49%)	5,108	(61.09%)	0.029
Antihypertensives	1,343	(16.05%)	17,806	(14.05%)	0.056	1,343	(16.06%)	1,263	(15.10%)	0.026
Estrogens	693	(8.28%)	15,799	(12.47%)	0.137	692	(8.28%)	672	(8.04%)	0.009
Immunosuppressants	1,373	(16.41%)	22,898	(18.07%)	0.044	1,372	(16.41%)	1,228	(14.69%)	0.048
Other antineoplastics										
Alkylating agents	905	(10.82%)	8,287	(6.54%)	0.152	904	(10.81%)	809	(9.67%)	0.037
Antimetabolites	1,377	(16.46%)	40,622	(32.05%)	0.370	1,377	(16.47%)	1,018	(12.17%)	0.123
Plant alkaloids and other natural products	510	(6.10%)	10,050	(7.93%)	0.072	510	(6.10%)	472	(5.64%)	0.019
Cytotoxic antibiotics and related substances	525	(6.28%)	6,738	(5.32%)	0.041	525	(6.28%)	527	(6.30%)	0.001
Monoclonal antibodies	1,070	(12.79%)	10,576	(8.35%)	0.145	1,067	(12.76%)	1,032	(12.34%)	0.013
Radiation Oncology Treatment	817	(9.77%)	7,396	(5.84%)	0.147	815	(9.75%)	760	(9.09%)	0.023
Cancer stages										
Stage 0	18	(0.22%)	703	(0.55%)	0.055	18	(0.22%)	20	(0.24%)	0.005
Stage 1	122	(1.46%)	2,050	(1.62%)	0.013	121	(1.45%)	110	(1.32%)	0.011
Stage 2	79	(0.94%)	1,517	(1.20%)	0.025	79	(0.94%)	73	(0.87%)	0.008
Stage 3	117	(1.40%)	1,178	(0.93%)	0.044	117	(1.40%)	109	(1.30%)	0.008
Stage 4	176	(2.10%)	982	(0.77%)	0.112	175	(2.09%)	144	(1.72%)	0.027

Abbreviations: SD, standard deviation; SMD, standardized mean difference; TKI, tyrosine kinase inhibitor

[a]*Data indexed at baseline was used*
