# Supplementary material for: Risks of Retinal Vascular Occlusions with the Systemic Use of Tyrosine Kinase Inhibitors
Source: Ophthalmol Retina. Author manuscript; Available in PMC 2026 Sep 21. (PMC13591267; doi:10.1016/j.oret.2026.02.019)
Supplement: Supplementary file 11 [file NIHMS2194774-supplement-19.pdf]

eTable 3Q: Results of the Propensity Score Matching Analysis Performed for the Risk Ratio Analysis Comparing the 2-Year TKI (Study) Cohort to the Non-TKI (Control) Cohort

	Before Matching					After Matching				
	**Study**		**Control**		**SMD**	**Study**		**Control**		**SMD**
Number of Patients	8,009		118,257			7,997		7,997		
Age at Index	63.01	(14.11)	63.45	(13.00)	0.033	62.99	(14.11)	62.49	(14.01)	0.035
Female	3,783	(47.23%)	69,224	(58.54%)	0.228	3,777	(47.23%)	3,815	(47.71%)	0.010
Ethnicity										
Hispanic or Latino	482	(6.02%)	4,416	(3.73%)	0.106	480	(6.00%)	457	(5.71%)	0.012
Unknown	1,241	(15.50%)	18,025	(15.24%)	0.007	1,241	(15.52%)	1,198	(14.98%)	0.015
Race										
White	5,918	(73.89%)	95,861	(81.06%)	0.172	5,908	(73.88%)	5,957	(74.49%)	0.014
Black or African American	896	(11.19%)	10,405	(8.80%)	0.080	895	(11.19%)	902	(11.28%)	0.003
Asian	397	(4.96%)	2,648	(2.24%)	0.146	397	(4.96%)	367	(4.59%)	0.018
Other	259	(3.23%)	2,410	(2.04%)	0.075	258	(3.23%)	252	(3.15%)	0.004
Unknown	496	(6.19%)	6,269	(5.30%)	0.038	496	(6.20%)	465	(5.81%)	0.016
[a]*Body Mass Index (kg/m²) [mean (SD)]*	28.10	(6.45)	28.92	(6.56)	0.125	28.11	(6.46)	28.61	(6.57)	0.077
<18.5	400	(4.99%)	4,171	(3.53%)	0.073	399	(4.99%)	385	(4.81%)	0.008
18.5-24.9	2,536	(31.66%)	32,548	(27.52%)	0.091	2,528	(31.61%)	2,389	(29.87%)	0.038
25-29.9	3,157	(39.42%)	43,816	(37.05%)	0.049	3,150	(39.39%)	3,009	(37.63%)	0.036
30-40	2,397	(29.93%)	36,702	(31.04%)	0.024	2,392	(29.91%)	2,376	(29.71%)	0.004
>40.0	646	(8.07%)	9,511	(8.04%)	0.001	645	(8.07%)	647	(8.09%)	0.001
Persons with potential health hazards related to socioeconomic and psychosocial circumstances	202	(2.52%)	2,712	(2.29%)	0.015	201	(2.51%)	222	(2.78%)	0.016
Tobacco use	200	(2.50%)	2,864	(2.42%)	0.005	200	(2.50%)	219	(2.74%)	0.015
Nicotine dependence	814	(10.16%)	13,135	(11.11%)	0.031	814	(10.18%)	844	(10.55%)	0.012
Alcohol related disorders	263	(3.28%)	4,340	(3.67%)	0.021	262	(3.28%)	267	(3.34%)	0.003
Diseases of the circulatory system	5,736	(71.62%)	80,286	(67.89%)	0.081	5,724	(71.58%)	5,576	(69.73%)	0.041
Hypertensive diseases	4,173	(52.10%)	60,519	(51.18%)	0.019	4,164	(52.07%)	4,057	(50.73%)	0.027
Ischemic heart diseases	1,511	(18.87%)	18,778	(15.88%)	0.079	1,505	(18.82%)	1,438	(17.98%)	0.022
Heart failure	643	(8.03%)	6,931	(5.86%)	0.085	639	(7.99%)	612	(7.65%)	0.013
Diseases of arteries, arterioles and capillaries	1,330	(16.61%)	20,007	(16.92%)	0.008	1,328	(16.61%)	1,252	(15.66%)	0.026
Diabetes mellitus	1,740	(21.73%)	22,350	(18.90%)	0.070	1,736	(21.71%)	1,701	(21.27%)	0.011
Cerebrovascular diseases	925	(11.55%)	10,994	(9.30%)	0.074	922	(11.53%)	878	(10.98%)	0.017
Chronic kidney disease (CKD)	1,085	(13.55%)	10,038	(8.49%)	0.162	1,080	(13.51%)	1,004	(12.55%)	0.028
Primary thrombophilia	107	(1.34%)	1,269	(1.07%)	0.024	106	(1.33%)	102	(1.28%)	0.004
Other thrombophilia	86	(1.07%)	1,023	(0.87%)	0.021	86	(1.08%)	91	(1.14%)	0.006
Polycythemia vera	266	(3.32%)	871	(0.74%)	0.184	264	(3.30%)	235	(2.94%)	0.021
Homocystinuria	10	(0.12%)	207	(0.18%)	0.013	10	(0.13%)	10	(0.13%)	0.000
Malignant immunoproliferative diseases and certain other B-cell lymphomas	280	(3.50%)	525	(0.44%)	0.221	269	(3.36%)	190	(2.38%)	0.059
Multiple myeloma and malignant plasma cell neoplasms	166	(2.07%)	4,248	(3.59%)	0.092	166	(2.08%)	169	(2.11%)	0.003
Systemic lupus erythematosus (SLE)	63	(0.79%)	1,695	(1.43%)	0.062	63	(0.79%)	72	(0.90%)	0.012
Glaucoma	843	(10.53%)	12,600	(10.65%)	0.004	841	(10.52%)	800	(10.00%)	0.017
Medications										
Antithrombotic agents	4,884	(60.98%)	61,085	(51.65%)	0.189	4,872	(60.92%)	4,702	(58.80%)	0.043
Antihypertensives	1,173	(14.65%)	15,532	(13.13%)	0.044	1,172	(14.66%)	1,121	(14.02%)	0.018
Estrogens	645	(8.05%)	15,283	(12.92%)	0.159	644	(8.05%)	618	(7.73%)	0.012
Immunosuppressants	1,132	(14.13%)	22,113	(18.70%)	0.123	1,132	(14.16%)	1,038	(12.98%)	0.034
Other antineoplastics										
Alkylating agents	772	(9.64%)	7,304	(6.18%)	0.129	768	(9.60%)	796	(9.95%)	0.012
Antimetabolites	1,193	(14.90%)	39,318	(33.25%)	0.440	1,193	(14.92%)	910	(11.38%)	0.105
Plant alkaloids and other natural products	424	(5.29%)	8,332	(7.05%)	0.073	424	(5.30%)	375	(4.69%)	0.028
Cytotoxic antibiotics and related substances	446	(5.57%)	5,931	(5.02%)	0.025	446	(5.58%)	455	(5.69%)	0.005
Monoclonal antibodies	933	(11.65%)	8,189	(6.92%)	0.163	926	(11.58%)	919	(11.49%)	0.003
Radiation Oncology Treatment	764	(9.54%)	6,403	(5.41%)	0.157	762	(9.53%)	766	(9.58%)	0.002
Cancer stages										
Stage 0	13	(0.16%)	573	(0.48%)	0.057	13	(0.16%)	11	(0.14%)	0.006
Stage 1	119	(1.49%)	1,627	(1.38%)	0.009	118	(1.48%)	99	(1.24%)	0.021
Stage 2	77	(0.96%)	1,199	(1.01%)	0.005	77	(0.96%)	64	(0.80%)	0.017
Stage 3	109	(1.36%)	952	(0.81%)	0.054	109	(1.36%)	108	(1.35%)	0.001
Stage 4	177	(2.21%)	867	(0.73%)	0.123	172	(2.15%)	158	(1.98%)	0.012

Abbreviations: SD, standard deviation; SMD, standardized mean difference; TKI, tyrosine kinase inhibitor
[a]*Data indexed at baseline was used*
