# Supplementary material for: Risks of Retinal Vascular Occlusions with the Systemic Use of Tyrosine Kinase Inhibitors
Source: Ophthalmol Retina. Author manuscript; Available in PMC 2026 Sep 21. (PMC13591267; doi:10.1016/j.oret.2026.02.019)
Supplement: Supplementary file 12 [file NIHMS2194774-supplement-2.pdf]

eTable 3A: Results of the Propensity Score Matching Analysis Performed for the Risk Ratio Analysis Comparing the TKI (Study) Cohort to the Non-TKI (Control) Cohort

	Before Matching					After Matching				
	Study		Control		SMD	Study		Control		SMD
Number of Patients	24,894		313,661			24,888		24,888		
Age at Index	64.62	(14.59)	65.31	(13.37)	0.049	64.61	(14.59)	64.60	(14.27)	0.001
Female	11,865	(47.66%)	174,618	(55.67%)	0.161	11,864	(47.67%)	11,701	(47.01%)	0.013
Ethnicity										
Hispanic or Latino	1,734	(6.97%)	14,968	(4.77%)	0.093	1,734	(6.97%)	1,802	(7.24%)	0.011
Unknown	3,978	(15.98%)	54,114	(17.25%)	0.034	3,977	(15.98%)	3,919	(15.75%)	0.006
Race										
White	18,036	(72.45%)	245,976	(78.42%)	0.139	18,030	(72.44%)	17,885	(71.86%)	0.013
Black or African American	2,678	(10.76%)	29,105	(9.28%)	0.049	2,678	(10.76%)	2,708	(10.88%)	0.004
Asian	1,395	(5.60%)	8,973	(2.86%)	0.137	1,395	(5.61%)	1,469	(5.90%)	0.013
Other	925	(3.72%)	8,233	(2.62%)	0.062	925	(3.72%)	917	(3.68%)	0.002
Unknown	1,691	(6.79%)	19,375	(6.18%)	0.025	1,691	(6.79%)	1,719	(6.91%)	0.004
[a]*Body Mass Index (kg/m²) [mean (SD)]*	27.76	(6.45)	28.72	(6.58)	0.149	27.75	(6.45)	28.10	(6.52)	0.054
<18.5	1,823	(7.32%)	14,683	(4.68%)	0.111	1,823	(7.32%)	1,768	(7.10%)	0.009
18.5-24.9	9,603	(38.58%)	100,341	(31.99%)	0.138	9,601	(38.58%)	9,422	(37.86%)	0.015
25-29.9	11,723	(47.09%)	134,544	(42.89%)	0.084	11,718	(47.08%)	11,379	(45.72%)	0.027
30-40	8,909	(35.79%)	111,341	(35.50%)	0.006	8,903	(35.77%)	8,670	(34.84%)	0.020
>40.0	2,368	(9.51%)	28,822	(9.19%)	0.011	2,367	(9.51%)	2,194	(8.82%)	0.024
Persons with potential health hazards related to socioeconomic and psychosocial circumstances	1,122	(4.51%)	11,703	(3.73%)	0.039	1,121	(4.50%)	1,078	(4.33%)	0.008
Tobacco use	947	(3.80%)	12,318	(3.93%)	0.006	947	(3.81%)	947	(3.81%)	0.000
Nicotine dependence	3,342	(13.42%)	42,235	(13.47%)	0.001	3,341	(13.42%)	3,201	(12.86%)	0.017
Alcohol related disorders	1,227	(4.93%)	14,683	(4.68%)	0.012	1,227	(4.93%)	1,206	(4.85%)	0.004
Diseases of the circulatory system	19,763	(79.39%)	232,315	(74.07%)	0.126	19,757	(79.38%)	19,259	(77.38%)	0.049
Hypertensive diseases	14,951	(60.06%)	181,042	(57.72%)	0.048	14,945	(60.05%)	14,507	(58.29%)	0.036
Ischemic heart diseases	6,220	(24.99%)	64,253	(20.48%)	0.108	6,217	(24.98%)	6,026	(24.21%)	0.018
Heart failure	3,095	(12.43%)	26,702	(8.51%)	0.128	3,092	(12.42%)	3,082	(12.38%)	0.001
Diseases of arteries, arterioles and capillaries	5,757	(23.13%)	64,427	(20.54%)	0.063	5,755	(23.12%)	5,518	(22.17%)	0.023
Diabetes mellitus	6,774	(27.21%)	73,679	(23.49%)	0.086	6,771	(27.21%)	6,651	(26.72%)	0.011
Cerebrovascular diseases	4,093	(16.44%)	39,450	(12.58%)	0.110	4,091	(16.44%)	3,934	(15.81%)	0.017
Chronic kidney disease (CKD)	4,496	(18.06%)	37,352	(11.91%)	0.173	4,491	(18.04%)	4,396	(17.66%)	0.010
Primary thrombophilia	469	(1.88%)	3,746	(1.19%)	0.056	467	(1.88%)	419	(1.68%)	0.015
Other thrombophilia	567	(2.28%)	3,283	(1.05%)	0.096	565	(2.27%)	502	(2.02%)	0.017
Polycythemia vera	543	(2.18%)	1,780	(0.57%)	0.139	542	(2.18%)	438	(1.76%)	0.030
Homocystinuria	49	(0.20%)	579	(0.18%)	0.003	49	(0.20%)	37	(0.15%)	0.012
Malignant immunoproliferative diseases and certain other B-cell lymphomas	757	(3.04%)	1,599	(0.51%)	0.193	751	(3.02%)	595	(2.39%)	0.039
Multiple myeloma and malignant plasma cell neoplasms	665	(2.67%)	9,156	(2.92%)	0.015	664	(2.67%)	607	(2.44%)	0.015
Systemic lupus erythematosus (SLE)	260	(1.04%)	3,797	(1.21%)	0.016	260	(1.04%)	265	(1.06%)	0.002
Glaucoma	3,329	(13.37%)	42,325	(13.49%)	0.004	3,327	(13.37%)	3,214	(12.91%)	0.013
Medications										
Antithrombotic agents	17,403	(69.91%)	185,970	(59.29%)	0.223	17,397	(69.90%)	17,117	(68.78%)	0.024
Antihypertensives	5,463	(21.95%)	58,556	(18.67%)	0.082	5,460	(21.94%)	5,178	(20.81%)	0.028
Estrogens	2,217	(8.91%)	35,629	(11.36%)	0.081	2,216	(8.90%)	2,095	(8.42%)	0.017
Immunosuppressants	4,762	(19.13%)	44,186	(14.09%)	0.136	4,759	(19.12%)	4,474	(17.98%)	0.029
Other antineoplastics										
Alkylating agents	2,820	(11.33%)	22,076	(7.04%)	0.149	2,817	(11.32%)	2,631	(10.57%)	0.024
Antimetabolites	5,178	(20.80%)	96,380	(30.73%)	0.228	5,177	(20.80%)	4,072	(16.36%)	0.114
Plant alkaloids and other natural products	2,433	(9.77%)	31,080	(9.91%)	0.005	2,433	(9.78%)	2,231	(8.96%)	0.028
Cytotoxic antibiotics and related substances	2,067	(8.30%)	20,289	(6.47%)	0.070	2,067	(8.31%)	1,988	(7.99%)	0.012
Monoclonal antibodies	4,094	(16.45%)	27,590	(8.80%)	0.232	4,088	(16.43%)	3,883	(15.60%)	0.022
Radiation Oncology Treatment	4,089	(16.43%)	25,179	(8.03%)	0.258	4,086	(16.42%)	3,984	(16.01%)	0.011
Cancer stages										
Stage 0	65	(0.26%)	1,775	(0.57%)	0.048	65	(0.26%)	60	(0.24%)	0.004
Stage 1	474	(1.90%)	6,022	(1.92%)	0.001	473	(1.90%)	449	(1.80%)	0.007
Stage 2	320	(1.29%)	4,386	(1.40%)	0.010	320	(1.29%)	307	(1.23%)	0.005
Stage 3	429	(1.72%)	3,589	(1.14%)	0.049	428	(1.72%)	423	(1.70%)	0.002
Stage 4	690	(2.77%)	3,460	(1.10%)	0.121	689	(2.77%)	694	(2.79%)	0.001

Abbreviations: SD, standard deviation; SMD, standardized mean difference; TKI, tyrosine kinase inhibitor

[a]*Data indexed at baseline was used*
