# Supplementary material for: Risks of Retinal Vascular Occlusions with the Systemic Use of Tyrosine Kinase Inhibitors
Source: Ophthalmol Retina. Author manuscript; Available in PMC 2026 Sep 21. (PMC13591267; doi:10.1016/j.oret.2026.02.019)
Supplement: Supplementary file 13 [file NIHMS2194774-supplement-20.pdf]

eTable 3R: Results of the Propensity Score Matching Analysis Performed for the Hazard Ratio Analysis Comparing the 2-Year TKI (Study) Cohort to the Non-TKI (Control) Cohort

	Before Matching					After Matching				
	Study		Control		SMD	Study		Control		SMD
Number of Patients	6,403		111,268			6,376		6,376		
Age at Index	62.44	(14.29)	66.84	(13.01)	0.322	62.44	(14.29)	62.03	(15.83)	0.028
Female	3,071	(47.96%)	65,930	(59.25%)	0.228	3,067	(48.10%)	3,094	(48.53%)	0.008
Ethnicity										
Hispanic or Latino	398	(6.22%)	4,094	(3.68%)	0.117	395	(6.20%)	364	(5.71%)	0.021
Unknown	1,036	(16.18%)	16,833	(15.13%)	0.029	1,034	(16.22%)	1,033	(16.20%)	0.000
Race										
White	4,723	(73.76%)	90,557	(81.39%)	0.184	4,704	(73.78%)	4,723	(74.07%)	0.007
Black or African American	725	(11.32%)	9,498	(8.54%)	0.093	723	(11.34%)	742	(11.64%)	0.009
Asian	298	(4.65%)	2,398	(2.16%)	0.138	295	(4.63%)	274	(4.30%)	0.016
Other	216	(3.37%)	2,256	(2.03%)	0.083	215	(3.37%)	192	(3.01%)	0.021
Unknown	408	(6.37%)	5,951	(5.35%)	0.044	406	(6.37%)	411	(6.45%)	0.003
[a]*Body Mass Index (kg/m²) [mean (SD)]*	28.23	(6.43)	28.76	(6.56)	0.082	28.23	(6.44)	28.70	(6.88)	0.071
<18.5	308	(4.81%)	7,097	(6.38%)	0.068	308	(4.83%)	344	(5.40%)	0.026
18.5-24.9	1,938	(30.27%)	41,943	(37.70%)	0.157	1,930	(30.27%)	1,936	(30.36%)	0.002
25-29.9	2,455	(38.34%)	55,029	(49.46%)	0.225	2,448	(38.39%)	2,463	(38.63%)	0.005
30-40	1,907	(29.78%)	44,542	(40.03%)	0.216	1,904	(29.86%)	1,859	(29.16%)	0.015
>40.0	511	(7.98%)	12,538	(11.27%)	0.112	511	(8.01%)	540	(8.47%)	0.017
Persons with potential health hazards related to socioeconomic and psychosocial circumstances	157	(2.45%)	5,816	(5.23%)	0.145	157	(2.46%)	182	(2.85%)	0.024
Tobacco use	149	(2.33%)	4,666	(4.19%)	0.105	149	(2.34%)	164	(2.57%)	0.015
Nicotine dependence	621	(9.70%)	16,143	(14.51%)	0.148	621	(9.74%)	622	(9.76%)	0.001
Alcohol related disorders	207	(3.23%)	6,273	(5.64%)	0.117	207	(3.25%)	199	(3.12%)	0.007
Diseases of the circulatory system	4,518	(70.56%)	94,544	(84.97%)	0.352	4,508	(70.70%)	4,425	(69.40%)	0.028
Hypertensive diseases	3,254	(50.82%)	72,737	(65.37%)	0.298	3,252	(51.00%)	3,175	(49.80%)	0.024
Ischemic heart diseases	1,150	(17.96%)	28,873	(25.95%)	0.194	1,146	(17.97%)	1,128	(17.69%)	0.007
Heart failure	468	(7.31%)	12,927	(11.62%)	0.148	467	(7.32%)	496	(7.78%)	0.017
Diseases of arteries, arterioles and capillaries	1,032	(16.12%)	34,803	(31.28%)	0.362	1,032	(16.19%)	1,052	(16.50%)	0.008
Diabetes mellitus	1,364	(21.30%)	28,874	(25.95%)	0.110	1,361	(21.35%)	1,316	(20.64%)	0.017
Cerebrovascular diseases	696	(10.87%)	18,727	(16.83%)	0.173	695	(10.90%)	689	(10.81%)	0.003
Chronic kidney disease (CKD)	802	(12.53%)	17,857	(16.05%)	0.101	800	(12.55%)	802	(12.58%)	0.001
Primary thrombophilia	81	(1.27%)	2,253	(2.02%)	0.060	81	(1.27%)	74	(1.16%)	0.010
Other thrombophilia	63	(0.98%)	2,006	(1.80%)	0.070	63	(0.99%)	71	(1.11%)	0.012
Polycythemia vera	211	(3.30%)	1,204	(1.08%)	0.152	211	(3.31%)	229	(3.59%)	0.015
Homocystinuria	10	(0.16%)	366	(0.33%)	0.035	10	(0.16%)	10	(0.16%)	0.000
Malignant immunoproliferative diseases and certain other B-cell lymphomas	245	(3.83%)	879	(0.79%)	0.203	223	(3.50%)	255	(4.00%)	0.026
Multiple myeloma and malignant plasma cell neoplasms	141	(2.20%)	4,680	(4.21%)	0.114	140	(2.20%)	165	(2.59%)	0.026
Systemic lupus erythematosus (SLE)	51	(0.80%)	2,281	(2.05%)	0.106	51	(0.80%)	57	(0.89%)	0.010
Glaucoma	682	(10.65%)	19,604	(17.62%)	0.201	682	(10.70%)	667	(10.46%)	0.008
Medications										
Antithrombotic agents	3,814	(59.57%)	79,016	(71.01%)	0.242	3,803	(59.65%)	3,764	(59.03%)	0.012
Antihypertensives	892	(13.93%)	26,868	(24.15%)	0.262	892	(13.99%)	876	(13.74%)	0.007
Estrogens	540	(8.43%)	20,759	(18.66%)	0.302	540	(8.47%)	561	(8.80%)	0.012
Immunosuppressants	920	(14.37%)	30,256	(27.19%)	0.320	919	(14.41%)	970	(15.21%)	0.023
Other antineoplastics										
Alkylating agents	604	(9.43%)	11,187	(10.05%)	0.021	601	(9.43%)	658	(10.32%)	0.030
Antimetabolites	931	(14.54%)	50,080	(45.01%)	0.707	931	(14.60%)	921	(14.44%)	0.004
Plant alkaloids and other natural products	306	(4.78%)	12,672	(11.39%)	0.244	306	(4.80%)	322	(5.05%)	0.012
Cytotoxic antibiotics and related substances	329	(5.14%)	8,808	(7.92%)	0.113	329	(5.16%)	325	(5.10%)	0.003
Monoclonal antibodies	726	(11.34%)	16,718	(15.02%)	0.109	723	(11.34%)	789	(12.37%)	0.032
Radiation Oncology Treatment	505	(7.89%)	13,136	(11.81%)	0.132	505	(7.92%)	503	(7.89%)	0.001
Cancer stages										
Stage 0	10	(0.16%)	776	(0.70%)	0.083	10	(0.16%)	20	(0.31%)	0.032
Stage 1	82	(1.28%)	2,244	(2.02%)	0.058	82	(1.29%)	78	(1.22%)	0.006
Stage 2	53	(0.83%)	1,410	(1.27%)	0.043	53	(0.83%)	60	(0.94%)	0.012
Stage 3	82	(1.28%)	982	(0.88%)	0.038	81	(1.27%)	75	(1.18%)	0.009
Stage 4	113	(1.76%)	807	(0.73%)	0.094	109	(1.71%)	96	(1.51%)	0.016

Abbreviations: SD, standard deviation; SMD, standardized mean difference; TKI, tyrosine kinase inhibitor

[a]*Data indexed at baseline was used*
