# Supplementary material for: Risks of Retinal Vascular Occlusions with the Systemic Use of Tyrosine Kinase Inhibitors
Source: Ophthalmol Retina. Author manuscript; Available in PMC 2026 Sep 21. (PMC13591267; doi:10.1016/j.oret.2026.02.019)
Supplement: Supplementary file 14 [file NIHMS2194774-supplement-21.pdf]

eTable 4A: Results of the risk ratio analyses using dental caries as the negative control outcome

Study cohort			Control Cohort			Risk Ratio	95% Confidence Interval
Name	Total	Event	Name	Total	Event		
TKI	24,030	595 (2.48%)	Non-TKI	23,996	649 (2.70%)	0.92	0.82-1.02
Non-anti-VEGF TKI	16,625	382 (2.30%)	Non-TKI	16,603	456 (2.75%)	0.84	0.73-0.96
Anti-VEGF TKI	6,468	142 (2.20%)	Non-TKI	6,435	178 (2.77%)	0.79	0.64-0.99
Non-BCR-ABL TKI	19,087	268 (1.40%)	Non-TKI	18,986	307 (1.62%)	0.87	0.74-1.02
BCR-ABL TKI	3,690	105 (2.85%)	Non-TKI	3,694	73 (1.98%)	1.44	1.07-1.93
BCR-ABL TKI	3,695	85 (2.30%)	Non-BCR-ABL TKI	3,699	57 (1.54%)	1.49	1.07-2.08

Abbreviations: TKI, tyrosine kinase inhibitor; VEGF, vascular endothelial growth factor
