# Supplementary material for: Risks of Retinal Vascular Occlusions with the Systemic Use of Tyrosine Kinase Inhibitors
Source: Ophthalmol Retina. Author manuscript; Available in PMC 2026 Sep 21. (PMC13591267; doi:10.1016/j.oret.2026.02.019)
Supplement: Supplementary file 15 [file NIHMS2194774-supplement-22.pdf]

eTable 4B: Results of the hazard ratio analyses using dental caries as the negative control outcome

Study cohort				Control Cohort				Hazard Ratio	95% Confidence Interval
Cohort Name	Total	Event		Name	Total	Event			
TKI	17,051	291	(1.71%)	Non-TKI	16,976	278	(1.64%)	1.16	0.98-1.37
Non-anti-VEGF TKI	11,944	182	(1.52%)	Non-TKI	11,939	178	(1.49%)	1.15	0.94-1.41
Anti-VEGF TKI	3,558	84	(2.36%)	Non-TKI	3,543	68	(1.92%)	1.57	1.14-2.16
Non-BCR-ABL TKI	17,456	204	(1.17%)	Non-TKI	17,449	202	(1.16%)	1.18	0.97-1.44
BCR-ABL TKI	3,637	71	(1.95%)	Non-TKI	3,612	52	(1.44%)	1.30	0.91-1.86
BCR-ABL TKI	3,573	68	(1.90%)	Non-BCR-ABL TKI	3,569	50	(1.40%)	1.11	0.77-1.61

Abbreviations: TKI, tyrosine kinase inhibitor; VEGF, vascular endothelial growth factor
