# Supplementary material for: Risks of Retinal Vascular Occlusions with the Systemic Use of Tyrosine Kinase Inhibitors
Source: Ophthalmol Retina. Author manuscript; Available in PMC 2026 Sep 21. (PMC13591267; doi:10.1016/j.oret.2026.02.019)
Supplement: Supplementary file 16 [file NIHMS2194774-supplement-23.pdf]

eFigure 1B: Results of the Sensitivity Analysis Comparing Patients With at Least a 6-Month Prescription Records of TKIs (Study Cohort) Versus Those With Non-TKI Antineoplastic Agents (Control Cohort) on the Hazards of Having Retinal Vascular Occlusions Within Three Years

Outcome	Study Events / Total	Control Events / Total	Hazard Ratio (95% CI)
Retinal vein occlusion	71 / 9 856	55 / 9 852	1.34 (0.94 – 1.90)
Central retinal vein occlusion	47 / 9 879	30 / 9 874	1.62 (1.02 – 2.56)
Branch retinal vein occlusion	30 / 9 875	33 / 9 872	0.95 (0.58 – 1.55)
Retinal artery occlusion	35 / 9 859	27 / 9 830	1.34 (0.81 – 2.22)

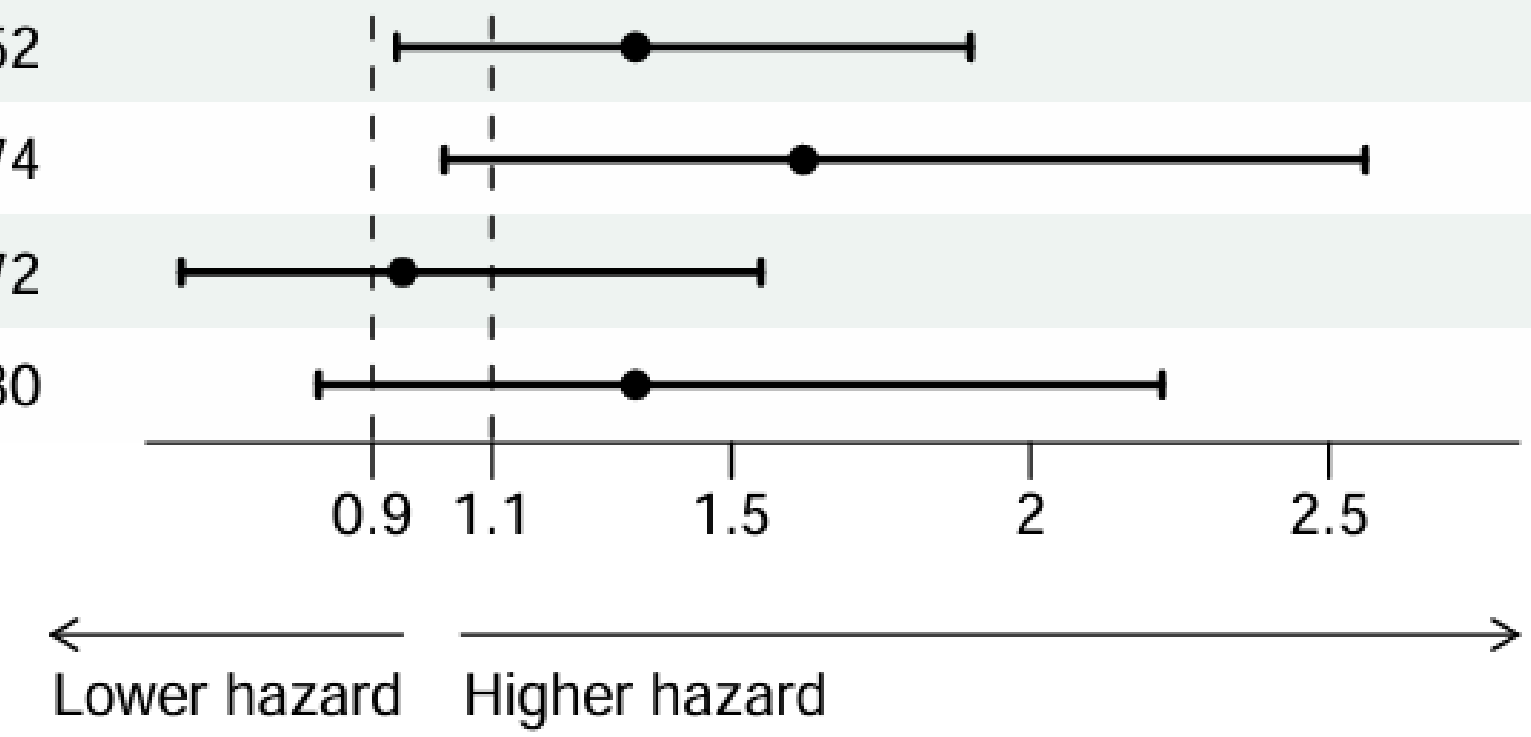

Abbreviation: TKI, tyrosine kinase inhibitor
Central and branch retinal artery occlusion: insufficient sample size
