# Supplementary material for: Risks of Retinal Vascular Occlusions with the Systemic Use of Tyrosine Kinase Inhibitors
Source: Ophthalmol Retina. Author manuscript; Available in PMC 2026 Sep 21. (PMC13591267; doi:10.1016/j.oret.2026.02.019)
Supplement: Supplementary file 17 [file NIHMS2194774-supplement-24.pdf]

eFigure 2A: Results of the Sensitivity Analysis Comparing Patients With at Least a 1-Year Prescription Records of TKIs (Study Cohort) Versus Those With Non-TKI Antineoplastic Agents (Control Cohort)

Outcome	Study Events / Total	Control Events / Total	Risk Ratio (95% CI)
Retinal vein occlusion	122 / 10 693	147 / 10 684	0.83 (0.65 – 1.05)
Central retinal vein occlusion	85 / 10 710	83 / 10 705	1.02 (0.76 – 1.38)
Branch retinal vein occlusion	56 / 10 715	92 / 10 707	0.61 (0.44 – 0.85)
Retinal artery occlusion	63 / 10 685	81 / 10 669	0.78 (0.56 – 1.08)
Central retinal artery occlusion	22 / 10 727	22 / 10 723	1.00 (0.55 – 1.80)
Branch retinal artery occlusion	22 / 10 724	27 / 10 718	0.81 (0.46 – 1.43)

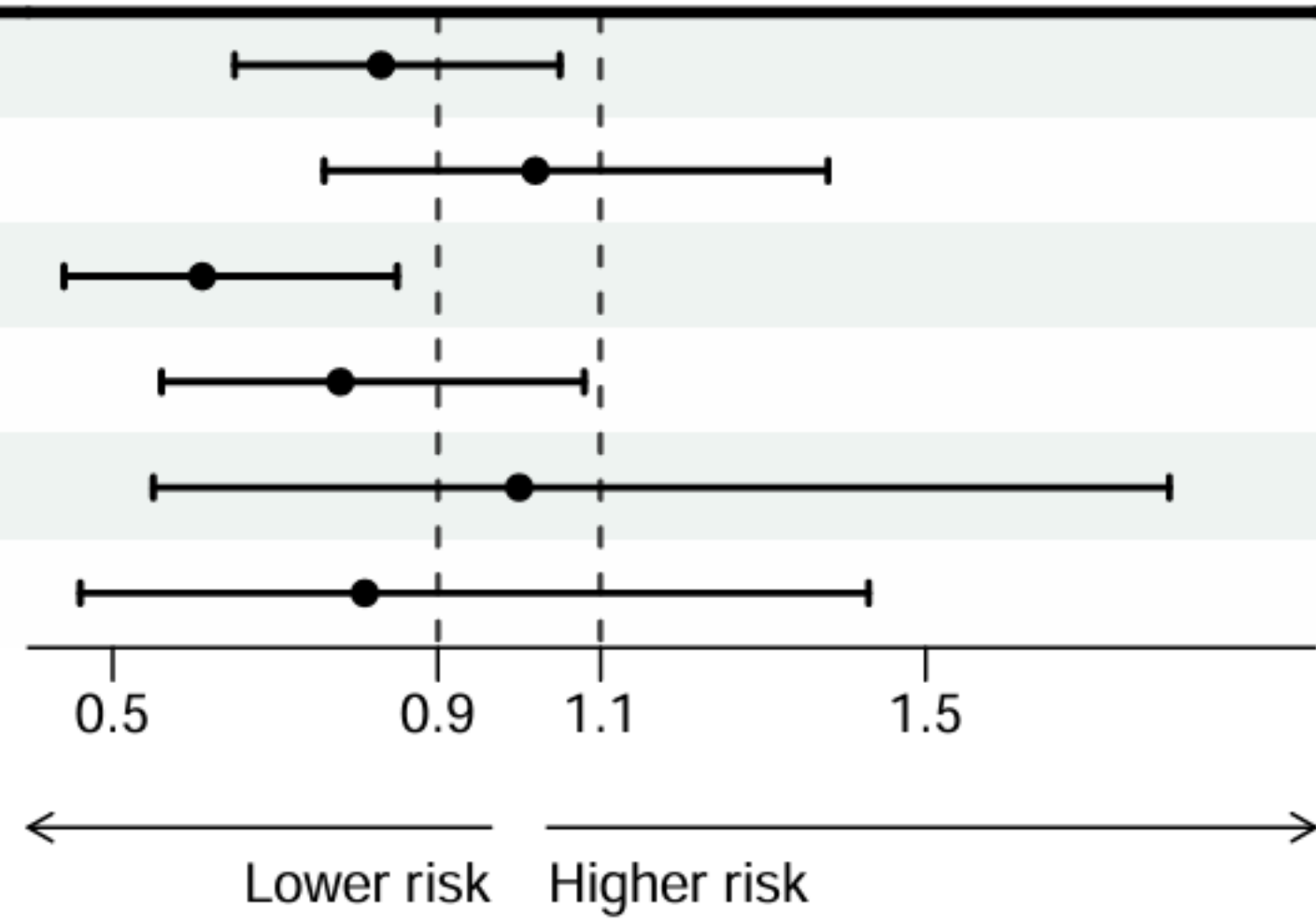

Abbreviation: TKI, tyrosine kinase inhibitor
