# Supplementary material for: Risks of Retinal Vascular Occlusions with the Systemic Use of Tyrosine Kinase Inhibitors
Source: Ophthalmol Retina. Author manuscript; Available in PMC 2026 Sep 21. (PMC13591267; doi:10.1016/j.oret.2026.02.019)
Supplement: Supplementary file 18 [file NIHMS2194774-supplement-25.pdf]

eFigure 2B: Results of the Sensitivity Analysis Comparing Patients With at Least a 1-Year Prescription Records of TKIs (Study Cohort) Versus Those With Non-TKI Antineoplastic Agents (Control Cohort) on the Hazards of Having Retinal Vascular Occlusions Within Three Years

Outcome	Study Events / Total	Control Events / Total	Hazard Ratio (95% CI)
Retinal vein occlusion	57 / 8 331	47 / 8 324	1.24 (0.84 – 1.82)
Central retinal vein occlusion	39 / 8 346	17 / 8 347	2.35 (1.33 – 4.15)
Branch retinal vein occlusion	23 / 8 345	34 / 8 335	0.69 (0.41 – 1.17)
Retinal artery occlusion	33 / 8 329	27 / 8 321	1.25 (0.75 – 2.07)

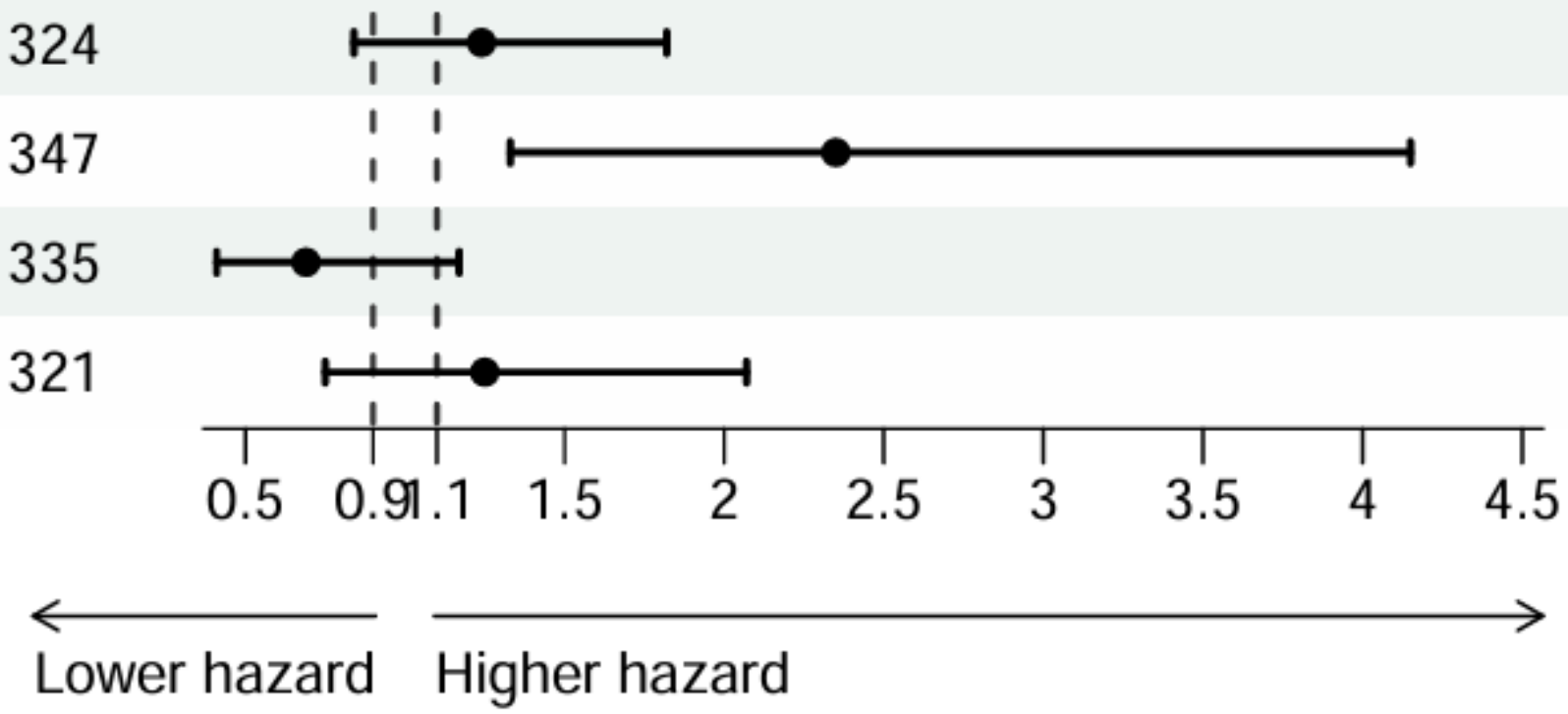

Abbreviation: TKI, tyrosine kinase inhibitor
Central and branch retinal artery occlusion: insufficient sample size
