# Supplementary material for: Risks of Retinal Vascular Occlusions with the Systemic Use of Tyrosine Kinase Inhibitors
Source: Ophthalmol Retina. Author manuscript; Available in PMC 2026 Sep 21. (PMC13591267; doi:10.1016/j.oret.2026.02.019)
Supplement: Supplementary file 19 [file NIHMS2194774-supplement-26.pdf]

eFigure 3B: Results of the Sensitivity Analysis Comparing Patients With at Least a 2-Year Prescription Records of TKIs (Study Cohort) Versus Those With Non-TKI Antineoplastic Agents (Control Cohort) on the Hazards of Having Retinal Vascular Occlusions Within Three Years

Outcome	Study Events / Total	Control Events / Total	Hazard Ratio (95% CI)
Retinal vein occlusion	63 / 6 350	34 / 6 315	1.58 (1.04 – 2.40)
Central retinal vein occlusion	41 / 6 362	16 / 6 342	2.20 (1.23 – 3.92)
Branch retinal vein occlusion	33 / 6 364	26 / 6 345	1.07 (0.64 – 1.80)
Retinal artery occlusion	29 / 6 360	23 / 6 342	1.07 (0.62 – 1.85)

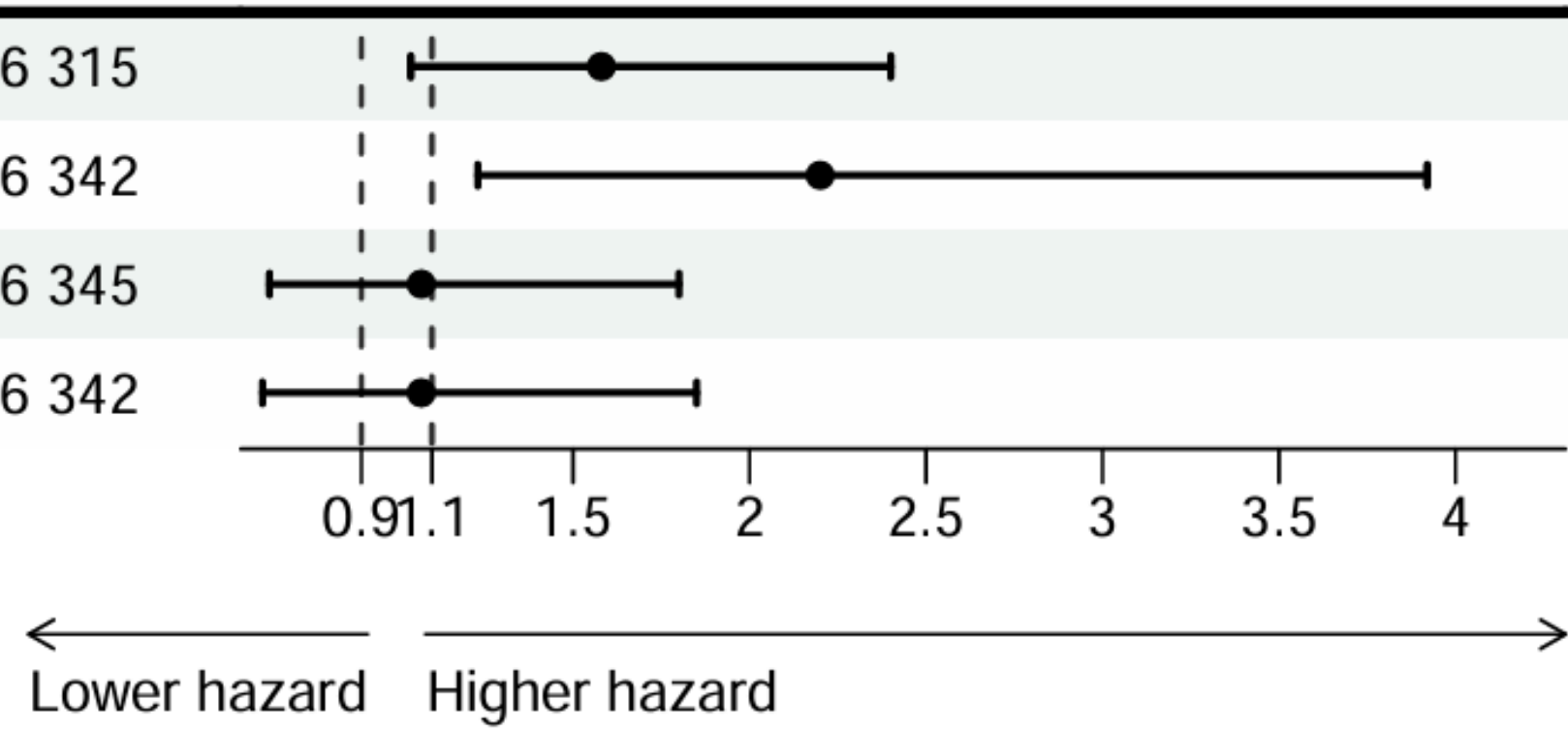

Abbreviation: TKI, tyrosine kinase inhibitor
Central and branch retinal artery occlusion: insufficient sample size
