# Supplementary material for: Risks of Retinal Vascular Occlusions with the Systemic Use of Tyrosine Kinase Inhibitors
Source: Ophthalmol Retina. Author manuscript; Available in PMC 2026 Sep 21. (PMC13591267; doi:10.1016/j.oret.2026.02.019)
Supplement: Supplementary file 20 [file NIHMS2194774-supplement-27.pdf]

eFigure 3A: Results of the Sensitivity Analysis Comparing Patients With at Least a 2-Year Prescription Records of TKIs (Study Cohort) Versus Those With Non-TKI Antineoplastic Agents (Control Cohort)

Outcome	Study Events / Total	Control Events / Total	Risk Ratio (95% CI)
Retinal vein occlusion	104 / 7 968	121 / 7 955	0.86 (0.66 – 1.11)
Central retinal vein occlusion	69 / 7 980	59 / 7 980	1.17 (0.83 – 1.65)
Branch retinal vein occlusion	51 / 7 984	81 / 7 967	0.63 (0.44 – 0.89)
Retinal artery occlusion	54 / 7 971	65 / 7 950	0.83 (0.58 – 1.19)
Central retinal artery occlusion	18 / 7 992	20 / 7 991	0.90 (0.48 – 1.70)
Branch retinal artery occlusion	18 / 7 991	23 / 7 980	0.78 (0.42 – 1.45)

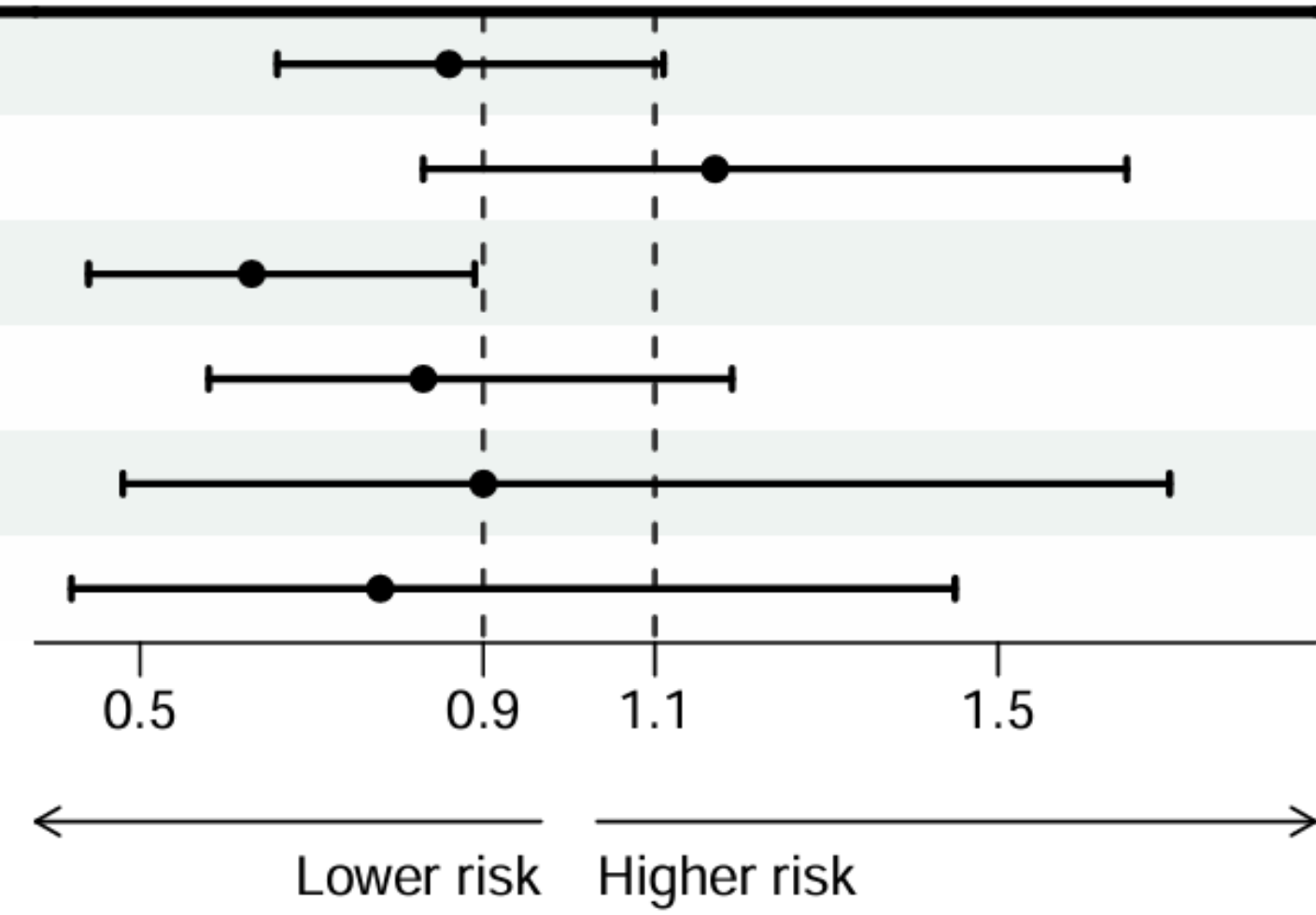

Abbreviation: TKI, tyrosine kinase inhibitor
