# Supplementary material for: Risks of Retinal Vascular Occlusions with the Systemic Use of Tyrosine Kinase Inhibitors
Source: Ophthalmol Retina. Author manuscript; Available in PMC 2026 Sep 21. (PMC13591267; doi:10.1016/j.oret.2026.02.019)
Supplement: Supplementary file 21 [file NIHMS2194774-supplement-28.pdf]

eTable 1: Relevant codes used for the analyses

Name	Code System	Relevant code
Cancer	ICD-10	C00-C96
Ophthalmic care	CPT; ICD-10; HCPCS	1012793; Z01.0, H25, Z96.1, Z98.4; S0620, S0621
Tyrosine kinase inhibitors (TKI)		
with anti-VEGF activity (anti-VEGF TKI):		
[a]Axitinib, [a]Fruquintinib, [a]Tivozanib, [b]Ponatinib, Cabozantinib, Lenvatinib, Nintedanib, Pazopanib, Regorafenib, Sorafenib, Sunitinib, Vandetanib	RxNorm	[a]1242999, [a]2670179, [a]2534233, [b]1364347, 1363268, 1603296, 1592737, 714438, 1312397, 495881, 357977, 1098413
without anti-VEGF activity (non-anti-VEGF TKI):		
Acalabrutinib, Afatinib, Alectinib, Asciminib, Avapritinib, Binimetinib, Bosutinib, Brigatinib, Capivasertib, Capmatinib, Ceritinib, Cobimetinib, Crizotinib, Dacomitinib, Dasatinib, Deucravacitinib, Entrectinib, Erdafitinib, Erlotinib, Fedratinib, Fostamatinib, Futibatinib, Gefitinib, Gilteritinib, Ibrutinib, Imatinib, Lapatinib, Larotrectinib, Lazertinib, Lorlatinib, Midostaurin, Mobocertinib, Momelotinib, Neratinib, Nilotinib, Osimertinib, Pacritinib, Pemigatinib, Pexidartinib, Pirtobrutinib, Pralsetinib, Quizartinib, Repotrectinib, Ripretinib, Ruxolitinib, Selpercatinib, Tepotinib, Tucatinib, Zanubrutinib	RxNorm	1986808, 1430438, 1727455, 2584304, 2272107, 2049122, 1307619, 1921217, 2669967, 2362165, 1535457, 1722365, 1148495, 2058849, 475342, 2612087, 2197862, 2123125, 337525, 2197490, 2044896, 2628190, 328134, 2105806, 1442981, 282388, 480167, 2105628, 2691150, 2103164, 1919083, 2570736, 2665204, 1940643, 662281, 1721560, 2595243, 2359268, 2183102, 2629338, 2394936, 2643048, 2670644, 2369389, 1193326, 2370147, 2477103, 2361285, 2262435
BCR-ABL TKI:		
Asciminib, Bosutinib, Dasatinib, Imatinib, Nilotinib, [b]Ponatinib	RxNorm	2584304, 1307619, 475342, 282388, 662281, [b]1364347
Non-BCR-ABL TKI:		
Acalabrutinib, Afatinib, Alectinib, Asciminib, Avapritinib, Axitinib, Binimetinib, Bosutinib, Brigatinib, Cabozantinib, Capmatinib, Ceritinib, Cobimetinib, Crizotinib, Dacomitinib, Dasatinib, Deucravacitinib, Entrectinib, Erdafitinib, Erlotinib, Fedratinib, Fostamatinib, Fruquintinib, Gefitinib, Gilteritinib, Ibrutinib, Imatinib, Larotrectinib, Lazertinib, Lenvatinib, Lorlatinib, Midostaurin, Mobocertinib, Neratinib, Nilotinib, Nintedanib, Osimertinib, Pazopanib, Pemigatinib, Pexidartinib, Ponatinib, Regorafenib, Ripretinib, Ruxolitinib, Selpercatinib, Sorafenib, Sunitinib, Tepotinib, Tivozanib, Tucatinib, Vandetanib, Zanubrutinib	RxNorm	1986808, 1430438, 1727455, 2584304, 2272107, 1242999, 2049122, 1307619, 1921217, 1363268, 2362165, 1535457, 1722365, 1148495, 2058849, 475342, 2612087, 2197862, 2123125, 337525, 2197490, 2044896, 2670179, 328134, 2105806, 1442981, 282388, 2105628, 2691150, 1603296, 2103164, 1919083, 2570736, 1940643, 662281, 1592737, 1721560, 714438, 2359268, 2183102, 1364347, 1312397, 2369389, 1193326, 2370147, 495881, 357977, 2477103, 2534233, 2361285, 1098413, 2262435
Antineoplastic agents	ATC	L01
Intravitreal anti-VEGF injection *(exclusion)*	ATC	S01LA
Confounding maculopathy (exclusion):		
Exudative age-related macular degeneration	ICD-10	H35.32
Diabetic macular edema	ICD-10	E10.311, E10.321, E10.331, E10.341, E10.35, E11.311, E11.321, E11.331, E11.341, E11.35
Proliferative diabetic retinopathy	ICD-10	E10.35, E11.35
Degenerative myopia with choroidal neovascularization	ICD-10	H44.2A
Retinal vein occlusion	ICD-10	H34.81, H34.83
Central retinal vein occlusion	ICD-10	H34.81
Branch retinal vein occlusion	ICD-10	H34.83
Retinal artery occlusion	ICD-10	H34.0-H34.2
Central retinal artery occlusion	ICD-10	H34.1
Branch retinal artery occlusion	ICD-10	H34.23
Negative control (dental caries)	ICD-10	K02.9

Abbreviations: ICD-10, International Classification of Diseases, 10th Revision; CPT, Current Procedural Terminology; HCPCS, Healthcare Common Procedure Coding System; ATC, Anatomical Therapeutic Chemical; VEGF, vascular endothelial growth factor

[a]Highly selective for VEGF receptor

[b]Ponatinib targets VEGF receptor, BCR-ABL, and FGFR
