# Supplementary material for: Risks of Retinal Vascular Occlusions with the Systemic Use of Tyrosine Kinase Inhibitors
Source: Ophthalmol Retina. Author manuscript; Available in PMC 2026 Sep 21. (PMC13591267; doi:10.1016/j.oret.2026.02.019)
Supplement: Supplementary file 22 [file NIHMS2194774-supplement-29.pdf]

eTable 2: Covariates and their relevant codes for propensity score matching

Covariate	Code System	Relevant code
Age at Index		
Female		
Ethnicity		
Hispanic or Latino		
Unknown		
Race		
White		
Black or African American		
Asian		
Other		
Unknown		
[a]*Body Mass Index (kg/m²)*	LOINC	39156-5
<18.5		
18.5-24.9		
25-29.9		
30-40		
>40.0		
Persons with potential health hazards related to socioeconomic and psychosocial circumstances	ICD-10	Z55-Z65
Smoking	ICD-10	F17, Z72.0
Alcohol related disorders	ICD-10	F10
Diseases of the circulatory system	ICD-10	I00-I99
Hypertensive diseases	ICD-10	I10-I1A
Ischemic heart diseases	ICD-10	I20-I25
Heart failure	ICD-10	I50
Diseases of arteries, arterioles and capillaries	ICD-10	I70-I79
Diabetes mellitus	ICD-10	E08-E13
Cerebrovascular diseases	ICD-10	I60-I69
Chronic kidney disease (CKD)	ICD-10	N18
Primary thrombophilia	ICD-10	D68.5
Other thrombophilia	ICD-10	D68.6
Polycythemia vera	ICD-10	D45
Homocystinuria	ICD-10	E72.11
Malignant immunoproliferative diseases and certain other B-cell lymphomas	ICD-10	C88
Multiple myeloma and malignant plasma cell neoplasms	ICD-10	C90
Systemic lupus erythematosus (SLE)	ICD-10	M32
Glaucoma	ICD-10	H40-H42
Medications		
Antithrombotic agents	ATC	B01
Antihypertensives	ATC	C02
Estrogens	ATC	G03C
Immunosuppressants	ATC	L04
Other antineoplastics		
Alkylating agents	ATC	L01A
Antimetabolites	ATC	L01B
Plant alkaloids and other natural products	ATC	L01C
Cytotoxic antibiotics and related substances	ATC	L01D
Monoclonal antibodies and antibody drug conjugates	ATC	L01F
Radiation Oncology Treatment	CPT	1010843
Cancer stages		
Stage 0	AJCC	S0
Stage 1	AJCC	S1
Stage 2	AJCC	S2
Stage 3	AJCC	S3
Stage 4	AJCC	S4

Abbreviations: ICD-10, International Classification of Diseases, 10th Revision; CPT, Current Procedural Terminology; LOINC, Logical Observation Identifiers Names and Codes; ATC, Anatomical Therapeutic Chemical; AJCC, American Joint Committee on Cancer

[a]*Data indexed at baseline*
