# Supplementary material for: Risks of Retinal Vascular Occlusions with the Systemic Use of Tyrosine Kinase Inhibitors
Source: Ophthalmol Retina. Author manuscript; Available in PMC 2026 Sep 21. (PMC13591267; doi:10.1016/j.oret.2026.02.019)
Supplement: Supplementary file 23 [file NIHMS2194774-supplement-3.pdf]

eTable 3B: Results of the Propensity Score Matching Analysis Performed for the Hazard Ratio Analysis Comparing the TKI (Study) Cohort to the Non-TKI (Control) Cohort

	Before Matching					After Matching				
	Study		Control		SMD	Study		Control		SMD
Number of Patients	17,591		281,634			17,590		17,590		
Age at Index	63.87	(14.82)	64.86	(13.36)	0.070	63.87	(14.82)	63.53	(14.56)	0.023
Female	8,619	(49.00%)	159,894	(56.77%)	0.156	8,619	(49.00%)	8,484	(48.23%)	0.015
Ethnicity										
Hispanic or Latino	1,252	(7.12%)	13,459	(4.78%)	0.099	1,252	(7.12%)	1,339	(7.61%)	0.019
Unknown	2,903	(16.50%)	48,334	(17.16%)	0.018	2,903	(16.50%)	2,844	(16.17%)	0.009
Race										
White	12,637	(71.84%)	221,748	(78.74%)	0.160	12,636	(71.84%)	12,483	(70.97%)	0.019
Black or African American	1,887	(10.73%)	25,210	(8.95%)	0.060	1,887	(10.73%)	1,922	(10.93%)	0.006
Asian	992	(5.64%)	7,870	(2.79%)	0.142	992	(5.64%)	1,029	(5.85%)	0.009
Other	689	(3.92%)	7,372	(2.62%)	0.073	689	(3.92%)	753	(4.28%)	0.018
Unknown	1,276	(7.25%)	17,689	(6.28%)	0.039	1,276	(7.25%)	1,305	(7.42%)	0.006
[a]*Body Mass Index (kg/m²) [mean (SD)]*	27.95	(6.43)	28.85	(6.56)	0.139	27.95	(6.43)	28.29	(6.47)	0.052
<18.5	1,178	(6.70%)	12,175	(4.32%)	0.104	1,177	(6.69%)	1,153	(6.55%)	0.005
18.5-24.9	6,461	(36.73%)	87,924	(31.22%)	0.117	6,460	(36.73%)	6,258	(35.58%)	0.024
25-29.9	7,934	(45.10%)	119,330	(42.37%)	0.055	7,933	(45.10%)	7,736	(43.98%)	0.023
30-40	6,080	(34.56%)	99,248	(35.24%)	0.014	6,080	(34.57%)	5,892	(33.50%)	0.023
>40.0	1,588	(9.03%)	25,699	(9.12%)	0.003	1,588	(9.03%)	1,437	(8.17%)	0.031
Persons with potential health hazards related to socioeconomic and psychosocial circumstances	762	(4.33%)	10,140	(3.60%)	0.037	762	(4.33%)	727	(4.13%)	0.010
Tobacco use	594	(3.38%)	9,921	(3.52%)	0.008	594	(3.38%)	575	(3.27%)	0.006
Nicotine dependence	2,087	(11.86%)	35,096	(12.46%)	0.018	2,087	(11.86%)	2,012	(11.44%)	0.013
Alcohol related disorders	737	(4.19%)	12,221	(4.34%)	0.007	737	(4.19%)	713	(4.05%)	0.007
Diseases of the circulatory system	13,460	(76.52%)	205,054	(72.81%)	0.085	13,459	(76.52%)	13,043	(74.15%)	0.055
Hypertensive diseases	10,020	(56.96%)	158,523	(56.29%)	0.014	10,020	(56.96%)	9,660	(54.92%)	0.041
Ischemic heart diseases	3,988	(22.67%)	53,830	(19.11%)	0.088	3,988	(22.67%)	3,774	(21.46%)	0.029
Heart failure	1,834	(10.43%)	20,770	(7.37%)	0.107	1,834	(10.43%)	1,762	(10.02%)	0.014
Diseases of arteries, arterioles and capillaries	3,781	(21.49%)	55,404	(19.67%)	0.045	3,780	(21.49%)	3,598	(20.45%)	0.025
Diabetes mellitus	4,433	(25.20%)	62,859	(22.32%)	0.068	4,433	(25.20%)	4,276	(24.31%)	0.021
Cerebrovascular diseases	2,594	(14.75%)	32,616	(11.58%)	0.094	2,593	(14.74%)	2,502	(14.22%)	0.015
Chronic kidney disease (CKD)	2,847	(16.18%)	30,470	(10.82%)	0.157	2,847	(16.19%)	2,769	(15.74%)	0.012
Primary thrombophilia	283	(1.61%)	3,248	(1.15%)	0.039	283	(1.61%)	256	(1.46%)	0.012
Other thrombophilia	335	(1.90%)	2,766	(0.98%)	0.077	335	(1.90%)	299	(1.70%)	0.015
Polycythemia vera	420	(2.39%)	1,573	(0.56%)	0.152	419	(2.38%)	321	(1.82%)	0.039
Homocystinuria	28	(0.16%)	521	(0.18%)	0.006	28	(0.16%)	23	(0.13%)	0.007
Malignant immunoproliferative diseases and certain other B-cell lymphomas	623	(3.54%)	1,398	(0.50%)	0.218	622	(3.54%)	478	(2.72%)	0.047
Multiple myeloma and malignant plasma cell neoplasms	458	(2.60%)	7,605	(2.70%)	0.006	458	(2.60%)	413	(2.35%)	0.016
Systemic lupus erythematosus (SLE)	187	(1.06%)	3,448	(1.22%)	0.015	187	(1.06%)	172	(0.98%)	0.008
Glaucoma	2,222	(12.63%)	36,979	(13.13%)	0.015	2,222	(12.63%)	2,111	(12.00%)	0.019
Medications										
Antithrombotic agents	11,631	(66.12%)	161,509	(57.35%)	0.181	11,630	(66.12%)	11,321	(64.36%)	0.037
Antihypertensives	3,528	(20.06%)	50,499	(17.93%)	0.054	3,528	(20.06%)	3,354	(19.07%)	0.025
Estrogens	1,700	(9.66%)	33,849	(12.02%)	0.076	1,700	(9.66%)	1,598	(9.08%)	0.020
Immunosuppressants	3,425	(19.47%)	40,401	(14.35%)	0.137	3,425	(19.47%)	3,145	(17.88%)	0.041
Other antineoplastics										
Alkylating agents	1,804	(10.26%)	18,459	(6.55%)	0.134	1,804	(10.26%)	1,666	(9.47%)	0.026
Antimetabolites	3,216	(18.28%)	85,374	(30.31%)	0.283	3,216	(18.28%)	2,461	(13.99%)	0.117
Plant alkaloids and other natural products	1,347	(7.66%)	23,574	(8.37%)	0.026	1,347	(7.66%)	1,248	(7.09%)	0.022
Cytotoxic antibiotics and related substances	1,210	(6.88%)	18,023	(6.40%)	0.019	1,210	(6.88%)	1,142	(6.49%)	0.015
Monoclonal antibodies	2,600	(14.78%)	22,401	(7.95%)	0.216	2,600	(14.78%)	2,478	(14.09%)	0.020
Radiation Oncology Treatment	2,252	(12.80%)	18,993	(6.74%)	0.205	2,252	(12.80%)	2,184	(12.42%)	0.012
Cancer stages										
Stage 0	45	(0.26%)	1,641	(0.58%)	0.051	45	(0.26%)	49	(0.28%)	0.004
Stage 1	304	(1.73%)	5,383	(1.91%)	0.014	304	(1.73%)	288	(1.64%)	0.007
Stage 2	176	(1.00%)	3,733	(1.33%)	0.030	176	(1.00%)	170	(0.97%)	0.003
Stage 3	264	(1.50%)	2,763	(0.98%)	0.047	264	(1.50%)	248	(1.41%)	0.008
Stage 4	342	(1.94%)	2,068	(0.73%)	0.105	342	(1.94%)	305	(1.73%)	0.016

Abbreviations: SD, standard deviation; SMD, standardized mean difference; TKI, tyrosine kinase inhibitor

[a]*Data indexed at baseline was used*
