# Supplementary material for: Risks of Retinal Vascular Occlusions with the Systemic Use of Tyrosine Kinase Inhibitors
Source: Ophthalmol Retina. Author manuscript; Available in PMC 2026 Sep 21. (PMC13591267; doi:10.1016/j.oret.2026.02.019)
Supplement: Supplementary file 24 [file NIHMS2194774-supplement-4.pdf]

eTable 3C: Results of the Propensity Score Matching Analysis Performed for the Risk Ratio Analysis Comparing the non-anti-VEGF TKI (Study) Cohort to the Non-TKI (Control) Cohort

	Before Matching					After Matching				
	Study		Control		SMD	Study		Control		SMD
Number of Patients	17,194		313,661			17,185		17,185		
Age at Index	64.52	(15.03)	65.31	(13.37)	0.055	64.52	(15.03)	64.14	(14.80)	0.026
Female	8,688	(50.53%)	174,618	(55.67%)	0.103	8,685	(50.54%)	8,492	(49.42%)	0.022
Ethnicity										
Hispanic or Latino	1,028	(5.98%)	14,968	(4.77%)	0.054	1,027	(5.98%)	1,060	(6.17%)	0.008
Unknown	2,737	(15.92%)	54,114	(17.25%)	0.036	2,737	(15.93%)	2,631	(15.31%)	0.017
Race										
White	12,569	(73.10%)	245,976	(78.42%)	0.124	12,564	(73.11%)	12,479	(72.62%)	0.011
Black or African American	1,713	(9.96%)	29,105	(9.28%)	0.023	1,712	(9.96%)	1,747	(10.17%)	0.007
Asian	1,023	(5.95%)	8,973	(2.86%)	0.151	1,021	(5.94%)	1,094	(6.37%)	0.018
Other	639	(3.72%)	8,233	(2.62%)	0.062	639	(3.72%)	655	(3.81%)	0.005
Unknown	1,148	(6.68%)	19,375	(6.18%)	0.020	1,147	(6.67%)	1,104	(6.42%)	0.010
[a]*Body Mass Index (kg/m²) [mean (SD)]*	27.41	(6.31)	28.72	(6.58)	0.204	27.41	(6.31)	27.83	(6.47)	0.065
<18.5	1,312	(7.63%)	14,683	(4.68%)	0.123	1,311	(7.63%)	1,330	(7.74%)	0.004
18.5-24.9	6,928	(40.29%)	100,341	(31.99%)	0.173	6,921	(40.27%)	6,737	(39.20%)	0.022
25-29.9	7,923	(46.08%)	134,544	(42.89%)	0.064	7,919	(46.08%)	7,593	(44.18%)	0.038
30-40	5,727	(33.31%)	111,341	(35.50%)	0.046	5,727	(33.33%)	5,617	(32.69%)	0.014
>40.0	1,474	(8.57%)	28,822	(9.19%)	0.022	1,474	(8.58%)	1,360	(7.91%)	0.024
Persons with potential health hazards related to socioeconomic and psychosocial circumstances	742	(4.32%)	11,703	(3.73%)	0.030	742	(4.32%)	725	(4.22%)	0.005
Tobacco use	582	(3.38%)	12,318	(3.93%)	0.029	582	(3.39%)	518	(3.01%)	0.021
Nicotine dependence	2,102	(12.23%)	42,235	(13.47%)	0.037	2,102	(12.23%)	2,018	(11.74%)	0.015
Alcohol related disorders	676	(3.93%)	14,683	(4.68%)	0.037	676	(3.93%)	618	(3.60%)	0.018
Diseases of the circulatory system	13,341	(77.59%)	232,315	(74.07%)	0.082	13,333	(77.59%)	12,969	(75.47%)	0.050
Hypertensive diseases	9,798	(56.98%)	181,042	(57.72%)	0.015	9,792	(56.98%)	9,484	(55.19%)	0.036
Ischemic heart diseases	3,993	(23.22%)	64,253	(20.48%)	0.066	3,990	(23.22%)	3,922	(22.82%)	0.009
Heart failure	2,064	(12.00%)	26,702	(8.51%)	0.115	2,062	(12.00%)	2,064	(12.01%)	0.000
Diseases of arteries, arterioles and capillaries	3,788	(22.03%)	64,427	(20.54%)	0.036	3,783	(22.01%)	3,580	(20.83%)	0.029
Diabetes mellitus	4,193	(24.39%)	73,679	(23.49%)	0.021	4,192	(24.39%)	4,092	(23.81%)	0.014
Cerebrovascular diseases	2,787	(16.21%)	39,450	(12.58%)	0.104	2,786	(16.21%)	2,679	(15.59%)	0.017
Chronic kidney disease (CKD)	2,773	(16.13%)	37,352	(11.91%)	0.122	2,771	(16.12%)	2,793	(16.25%)	0.003
Primary thrombophilia	343	(1.99%)	3,746	(1.19%)	0.064	341	(1.98%)	307	(1.79%)	0.015
Other thrombophilia	422	(2.45%)	3,283	(1.05%)	0.107	419	(2.44%)	384	(2.23%)	0.013
Polycythemia vera	493	(2.87%)	1,780	(0.57%)	0.178	491	(2.86%)	498	(2.90%)	0.002
Homocystinuria	35	(0.20%)	579	(0.18%)	0.004	35	(0.20%)	29	(0.17%)	0.008
Malignant immunoproliferative diseases and certain other B-cell lymphomas	720	(4.19%)	1,599	(0.51%)	0.245	712	(4.14%)	597	(3.47%)	0.035
Multiple myeloma and malignant plasma cell neoplasms	567	(3.30%)	9,156	(2.92%)	0.022	566	(3.29%)	510	(2.97%)	0.019
Systemic lupus erythematosus (SLE)	181	(1.05%)	3,797	(1.21%)	0.015	181	(1.05%)	163	(0.95%)	0.011
Glaucoma	2,248	(13.07%)	42,325	(13.49%)	0.012	2,246	(13.07%)	2,173	(12.64%)	0.013
Medications										
Antithrombotic agents	11,728	(68.21%)	185,970	(59.29%)	0.186	11,719	(68.19%)	11,384	(66.24%)	0.042
Antihypertensives	3,479	(20.23%)	58,556	(18.67%)	0.040	3,476	(20.23%)	3,327	(19.36%)	0.022
Estrogens	1,718	(9.99%)	35,629	(11.36%)	0.044	1,717	(9.99%)	1,565	(9.11%)	0.030
Immunosuppressants	3,617	(21.04%)	44,186	(14.09%)	0.183	3,613	(21.02%)	3,323	(19.34%)	0.042
Other antineoplastics										
Alkylating agents	2,353	(13.69%)	22,076	(7.04%)	0.219	2,346	(13.65%)	2,167	(12.61%)	0.031
Antimetabolites	4,006	(23.30%)	96,380	(30.73%)	0.168	4,005	(23.31%)	3,139	(18.27%)	0.124
Plant alkaloids and other natural products	1,596	(9.28%)	31,080	(9.91%)	0.021	1,595	(9.28%)	1,446	(8.41%)	0.031
Cytotoxic antibiotics and related substances	1,334	(7.76%)	20,289	(6.47%)	0.050	1,333	(7.76%)	1,215	(7.07%)	0.026
Monoclonal antibodies	3,045	(17.71%)	27,590	(8.80%)	0.265	3,037	(17.67%)	2,938	(17.10%)	0.015
Radiation Oncology Treatment	2,506	(14.57%)	25,179	(8.03%)	0.208	2,505	(14.58%)	2,422	(14.09%)	0.014
Cancer stages										
Stage 0	44	(0.26%)	1,775	(0.57%)	0.048	44	(0.26%)	41	(0.24%)	0.004
Stage 1	261	(1.52%)	6,022	(1.92%)	0.031	261	(1.52%)	242	(1.41%)	0.009
Stage 2	172	(1.00%)	4,386	(1.40%)	0.037	172	(1.00%)	156	(0.91%)	0.010
Stage 3	198	(1.15%)	3,589	(1.14%)	0.001	198	(1.15%)	194	(1.13%)	0.002
Stage 4	427	(2.48%)	3,460	(1.10%)	0.104	427	(2.48%)	429	(2.50%)	0.001

Abbreviations: Abbreviations: SD, standard deviation; SMD, standardized mean difference; TKI, tyrosine kinase inhibitor; VEGF, vascular endothelial growth factor

[a]*Data indexed at baseline was used*
