# Supplementary material for: Risks of Retinal Vascular Occlusions with the Systemic Use of Tyrosine Kinase Inhibitors
Source: Ophthalmol Retina. Author manuscript; Available in PMC 2026 Sep 21. (PMC13591267; doi:10.1016/j.oret.2026.02.019)
Supplement: Supplementary file 25 [file NIHMS2194774-supplement-5.pdf]

eTable 3D: Results of the Propensity Score Matching Analysis Performed for the Hazard Ratio Analysis Comparing the non-anti-VEGF TKI (Study) Cohort to the Non-TKI (Control) Cohort

	Before Matching					After Matching				
	Study		Control		SMD	Study		Control		SMD
Number of Patients	12,321		281,634			12,313		12,313		
Age at Index	63.37	(15.28)	64.86	(13.36)	0.104	63.37	(15.28)	63.15	(14.94)	0.014
Female	6,337	(51.43%)	159,894	(56.77%)	0.107	6,336	(51.46%)	6,249	(50.75%)	0.014
Ethnicity										
Hispanic or Latino	777	(6.31%)	13,459	(4.78%)	0.067	777	(6.31%)	774	(6.29%)	0.001
Unknown	2,016	(16.36%)	48,334	(17.16%)	0.021	2,015	(16.36%)	1,989	(16.15%)	0.006
Race										
White	8,874	(72.02%)	221,748	(78.74%)	0.156	8,869	(72.03%)	8,783	(71.33%)	0.016
Black or African American	1,290	(10.47%)	25,210	(8.95%)	0.051	1,289	(10.47%)	1,314	(10.67%)	0.007
Asian	710	(5.76%)	7,870	(2.79%)	0.147	709	(5.76%)	718	(5.83%)	0.003
Other	492	(3.99%)	7,372	(2.62%)	0.077	492	(4.00%)	521	(4.23%)	0.012
Unknown	885	(7.18%)	17,689	(6.28%)	0.036	884	(7.18%)	907	(7.37%)	0.007
[a]*Body Mass Index (kg/m²) [mean (SD)]*	27.69	(6.31)	28.85	(6.56)	0.180	27.69	(6.31)	28.01	(6.51)	0.050
<18.5	851	(6.91%)	12,175	(4.32%)	0.112	850	(6.90%)	809	(6.57%)	0.013
18.5-24.9	4,685	(38.02%)	87,924	(31.22%)	0.143	4,680	(38.01%)	4,539	(36.86%)	0.024
25-29.9	5,477	(44.45%)	119,330	(42.37%)	0.042	5,473	(44.45%)	5,287	(42.94%)	0.030
30-40	4,021	(32.64%)	99,248	(35.24%)	0.055	4,020	(32.65%)	3,815	(30.98%)	0.036
>40.0	1,043	(8.47%)	25,699	(9.12%)	0.023	1,043	(8.47%)	975	(7.92%)	0.020
Persons with potential health hazards related to socioeconomic and psychosocial circumstances	538	(4.37%)	10,140	(3.60%)	0.039	538	(4.37%)	515	(4.18%)	0.009
Tobacco use	391	(3.17%)	9,921	(3.52%)	0.019	390	(3.17%)	346	(2.81%)	0.021
Nicotine dependence	1,374	(11.15%)	35,096	(12.46%)	0.041	1,373	(11.15%)	1,235	(10.03%)	0.036
Alcohol related disorders	450	(3.65%)	12,221	(4.34%)	0.035	450	(3.65%)	404	(3.28%)	0.020
Diseases of the circulatory system	9,205	(74.71%)	205,054	(72.81%)	0.043	9,197	(74.69%)	8,855	(71.92%)	0.063
Hypertensive diseases	6,629	(53.80%)	158,523	(56.29%)	0.050	6,623	(53.79%)	6,390	(51.90%)	0.038
Ischemic heart diseases	2,565	(20.82%)	53,830	(19.11%)	0.043	2,562	(20.81%)	2,442	(19.83%)	0.024
Heart failure	1,201	(9.75%)	20,770	(7.37%)	0.085	1,200	(9.75%)	1,146	(9.31%)	0.015
Diseases of arteries, arterioles and capillaries	2,514	(20.40%)	55,404	(19.67%)	0.018	2,512	(20.40%)	2,321	(18.85%)	0.039
Diabetes mellitus	2,804	(22.76%)	62,859	(22.32%)	0.010	2,803	(22.76%)	2,708	(21.99%)	0.019
Cerebrovascular diseases	1,776	(14.41%)	32,616	(11.58%)	0.084	1,773	(14.40%)	1,666	(13.53%)	0.025
Chronic kidney disease (CKD)	1,770	(14.37%)	30,470	(10.82%)	0.107	1,769	(14.37%)	1,747	(14.19%)	0.005
Primary thrombophilia	214	(1.74%)	3,248	(1.15%)	0.049	213	(1.73%)	200	(1.62%)	0.008
Other thrombophilia	249	(2.02%)	2,766	(0.98%)	0.085	248	(2.01%)	235	(1.91%)	0.008
Polycythemia vera	340	(2.76%)	1,573	(0.56%)	0.173	338	(2.75%)	327	(2.66%)	0.006
Homocystinuria	18	(0.15%)	521	(0.18%)	0.010	18	(0.15%)	15	(0.12%)	0.007
Malignant immunoproliferative diseases and certain other B-cell lymphomas	569	(4.62%)	1,398	(0.50%)	0.263	561	(4.56%)	420	(3.41%)	0.059
Multiple myeloma and malignant plasma cell neoplasms	389	(3.16%)	7,605	(2.70%)	0.027	388	(3.15%)	371	(3.01%)	0.008
Systemic lupus erythematosus (SLE)	138	(1.12%)	3,448	(1.22%)	0.010	138	(1.12%)	114	(0.93%)	0.019
Glaucoma	1,560	(12.66%)	36,979	(13.13%)	0.014	1,560	(12.67%)	1,436	(11.66%)	0.031
Medications										
Antithrombotic agents	7,959	(64.60%)	161,509	(57.35%)	0.149	7,952	(64.58%)	7,661	(62.22%)	0.049
Antihypertensives	2,333	(18.94%)	50,499	(17.93%)	0.026	2,328	(18.91%)	2,212	(17.96%)	0.024
Estrogens	1,331	(10.80%)	33,849	(12.02%)	0.038	1,331	(10.81%)	1,247	(10.13%)	0.022
Immunosuppressants	2,595	(21.06%)	40,401	(14.35%)	0.177	2,590	(21.03%)	2,449	(19.89%)	0.028
Other antineoplastics										
Alkylating agents	1,485	(12.05%)	18,459	(6.55%)	0.190	1,479	(12.01%)	1,403	(11.39%)	0.019
Antimetabolites	2,502	(20.31%)	85,374	(30.31%)	0.232	2,500	(20.30%)	1,929	(15.67%)	0.121
Plant alkaloids and other natural products	926	(7.52%)	23,574	(8.37%)	0.032	926	(7.52%)	841	(6.83%)	0.027
Cytotoxic antibiotics and related substances	836	(6.79%)	18,023	(6.40%)	0.016	836	(6.79%)	754	(6.12%)	0.027
Monoclonal antibodies	1,919	(15.58%)	22,401	(7.95%)	0.238	1,913	(15.54%)	1,850	(15.02%)	0.014
Radiation Oncology Treatment	1,430	(11.61%)	18,993	(6.74%)	0.169	1,429	(11.61%)	1,386	(11.26%)	0.011
Cancer stages										
Stage 0	30	(0.24%)	1,641	(0.58%)	0.053	30	(0.24%)	32	(0.26%)	0.003
Stage 1	186	(1.51%)	5,383	(1.91%)	0.031	186	(1.51%)	163	(1.32%)	0.016
Stage 2	105	(0.85%)	3,733	(1.33%)	0.046	105	(0.85%)	92	(0.75%)	0.012
Stage 3	126	(1.02%)	2,763	(0.98%)	0.004	126	(1.02%)	112	(0.91%)	0.012
Stage 4	229	(1.86%)	2,068	(0.73%)	0.100	228	(1.85%)	244	(1.98%)	0.009

Abbreviations: Abbreviations: SD, standard deviation; SMD, standardized mean difference; TKI, tyrosine kinase inhibitor; VEGF, vascular endothelial growth factor

[a]*Data indexed at baseline was used*
