# Supplementary material for: Risks of Retinal Vascular Occlusions with the Systemic Use of Tyrosine Kinase Inhibitors
Source: Ophthalmol Retina. Author manuscript; Available in PMC 2026 Sep 21. (PMC13591267; doi:10.1016/j.oret.2026.02.019)
Supplement: Supplementary file 26 [file NIHMS2194774-supplement-6.pdf]

eTable 3E: Results of the Propensity Score Matching Analysis Performed for the Risk Ratio Analysis Comparing the anti-VEGF TKI (Study) Cohort to the Non-TKI (Control) Cohort

	Before Matching					After Matching				
	Study		Control		SMD	Study		Control		SMD
Number of Patients	6,733		333,644			6,728		6,728		
Age at Index	65.82	(12.69)	65.52	(13.25)	0.023	65.82	(12.69)	65.55	(13.57)	0.021
Female	2,740	(40.70%)	186,361	(55.86%)	0.307	2,740	(40.73%)	2,596	(38.59%)	0.044
Ethnicity										
Hispanic or Latino	588	(8.73%)	15,250	(4.57%)	0.168	587	(8.72%)	567	(8.43%)	0.011
Unknown	995	(14.78%)	50,222	(15.05%)	0.008	995	(14.79%)	955	(14.19%)	0.017
Race										
White	4,814	(71.50%)	263,382	(78.94%)	0.173	4,813	(71.54%)	4,743	(70.50%)	0.023
Black or African American	880	(13.07%)	31,177	(9.34%)	0.118	878	(13.05%)	939	(13.96%)	0.027
Asian	306	(4.54%)	9,252	(2.77%)	0.094	305	(4.53%)	303	(4.50%)	0.001
Other	216	(3.21%)	8,252	(2.47%)	0.044	216	(3.21%)	211	(3.14%)	0.004
Unknown	457	(6.79%)	19,529	(5.85%)	0.038	457	(6.79%)	451	(6.70%)	0.004
[a]*Body Mass Index (kg/m²) [mean (SD)]*	28.56	(6.66)	28.72	(6.56)	0.023	28.57	(6.66)	29.05	(6.71)	0.073
<18.5	443	(6.58%)	14,389	(4.31%)	0.100	443	(6.58%)	441	(6.55%)	0.001
18.5-24.9	2,240	(33.27%)	101,424	(30.40%)	0.062	2,237	(33.25%)	2,188	(32.52%)	0.016
25-29.9	3,210	(47.68%)	136,182	(40.82%)	0.138	3,206	(47.65%)	3,185	(47.34%)	0.006
30-40	2,750	(40.84%)	112,191	(33.63%)	0.150	2,748	(40.84%)	2,739	(40.71%)	0.003
>40.0	779	(11.57%)	28,757	(8.62%)	0.098	778	(11.56%)	781	(11.61%)	0.001
Persons with potential health hazards related to socioeconomic and psychosocial circumstances	340	(5.05%)	12,052	(3.61%)	0.071	339	(5.04%)	338	(5.02%)	0.001
Tobacco use	345	(5.12%)	13,105	(3.93%)	0.058	344	(5.11%)	359	(5.34%)	0.010
Nicotine dependence	1,139	(16.92%)	44,312	(13.28%)	0.102	1,138	(16.91%)	1,166	(17.33%)	0.011
Alcohol related disorders	520	(7.72%)	15,538	(4.66%)	0.127	519	(7.71%)	500	(7.43%)	0.011
Diseases of the circulatory system	5,783	(85.89%)	247,221	(74.10%)	0.298	5,778	(85.88%)	5,727	(85.12%)	0.022
Hypertensive diseases	4,726	(70.19%)	192,750	(57.77%)	0.261	4,721	(70.17%)	4,710	(70.01%)	0.004
Ischemic heart diseases	2,063	(30.64%)	68,514	(20.54%)	0.233	2,059	(30.60%)	2,057	(30.57%)	0.001
Heart failure	985	(14.63%)	28,483	(8.54%)	0.191	984	(14.63%)	974	(14.48%)	0.004
Diseases of arteries, arterioles and capillaries	1,859	(27.61%)	69,268	(20.76%)	0.160	1,856	(27.59%)	1,807	(26.86%)	0.016
Diabetes mellitus	2,378	(35.32%)	78,382	(23.49%)	0.262	2,374	(35.29%)	2,351	(34.94%)	0.007
Cerebrovascular diseases	1,212	(18.00%)	41,894	(12.56%)	0.152	1,210	(17.98%)	1,139	(16.93%)	0.028
Chronic kidney disease (CKD)	1,647	(24.46%)	39,835	(11.94%)	0.329	1,642	(24.41%)	1,591	(23.65%)	0.018
Primary thrombophilia	109	(1.62%)	3,910	(1.17%)	0.038	109	(1.62%)	107	(1.59%)	0.002
Other thrombophilia	127	(1.89%)	3,375	(1.01%)	0.073	127	(1.89%)	107	(1.59%)	0.023
Polycythemia vera	32	(0.48%)	1,845	(0.55%)	0.011	32	(0.48%)	32	(0.48%)	0.000
Homocystinuria	14	(0.21%)	606	(0.18%)	0.006	14	(0.21%)	10	(0.15%)	0.014
Malignant immunoproliferative diseases and certain other B-cell lymphomas	18	(0.27%)	1,677	(0.50%)	0.038	18	(0.27%)	19	(0.28%)	0.003
Multiple myeloma and malignant plasma cell neoplasms	79	(1.17%)	9,519	(2.85%)	0.120	79	(1.17%)	86	(1.28%)	0.009
Systemic lupus erythematosus (SLE)	74	(1.10%)	3,941	(1.18%)	0.008	74	(1.10%)	63	(0.94%)	0.016
Glaucoma	1,022	(15.18%)	45,135	(13.53%)	0.047	1,021	(15.18%)	964	(14.33%)	0.024
Medications										
Antithrombotic agents	5,121	(76.06%)	197,514	(59.20%)	0.366	5,116	(76.04%)	5,046	(75.00%)	0.024
Antihypertensives	1,869	(27.76%)	62,153	(18.63%)	0.218	1,866	(27.73%)	1,792	(26.63%)	0.025
Estrogens	435	(6.46%)	37,795	(11.33%)	0.172	435	(6.47%)	394	(5.86%)	0.025
Immunosuppressants	993	(14.75%)	47,019	(14.09%)	0.019	992	(14.74%)	928	(13.79%)	0.027
Other antineoplastics										
Alkylating agents	363	(5.39%)	25,167	(7.54%)	0.088	363	(5.40%)	394	(5.86%)	0.020
Antimetabolites	957	(14.21%)	102,270	(30.65%)	0.402	957	(14.22%)	845	(12.56%)	0.049
Plant alkaloids and other natural products	779	(11.57%)	32,841	(9.84%)	0.056	779	(11.58%)	774	(11.50%)	0.002
Cytotoxic antibiotics and related substances	632	(9.39%)	20,847	(6.25%)	0.117	631	(9.38%)	638	(9.48%)	0.004
Monoclonal antibodies	978	(14.53%)	29,849	(8.95%)	0.174	976	(14.51%)	958	(14.24%)	0.008
Radiation Oncology Treatment	1,520	(22.58%)	26,703	(8.00%)	0.413	1,515	(22.52%)	1,450	(21.55%)	0.023
Cancer stages										
Stage 0	17	(0.25%)	1,299	(0.39%)	0.024	17	(0.25%)	14	(0.21%)	0.009
Stage 1	167	(2.48%)	4,812	(1.44%)	0.075	165	(2.45%)	158	(2.35%)	0.007
Stage 2	111	(1.65%)	3,406	(1.02%)	0.055	111	(1.65%)	104	(1.55%)	0.008
Stage 3	178	(2.64%)	2,854	(0.86%)	0.137	174	(2.59%)	173	(2.57%)	0.001
Stage 4	180	(2.67%)	2,740	(0.82%)	0.142	180	(2.68%)	168	(2.50%)	0.011

Abbreviations: Abbreviations: SD, standard deviation; SMD, standardized mean difference; TKI, tyrosine kinase inhibitor; VEGF, vascular endothelial growth factor

[a]*Data indexed at baseline was used*
