# Supplementary material for: Risks of Retinal Vascular Occlusions with the Systemic Use of Tyrosine Kinase Inhibitors
Source: Ophthalmol Retina. Author manuscript; Available in PMC 2026 Sep 21. (PMC13591267; doi:10.1016/j.oret.2026.02.019)
Supplement: Supplementary file 27 [file NIHMS2194774-supplement-7.pdf]

eTable 3F: Results of the Propensity Score Matching Analysis Performed for the Hazard Ratio Analysis Comparing the anti-VEGF TKI (Study) Cohort to the Non-TKI (Control) Cohort

	Before Matching					After Matching				
	Study		Control		SMD	Study		Control		SMD
Number of Patients	3,684		289,820			3,676		3,676		
Age at Index	65.68	(12.68)	64.94	(13.24)	0.057	65.68	(12.68)	65.53	(13.16)	0.012
Female	1,550	(42.07%)	165,889	(57.24%)	0.307	1,550	(42.17%)	1,513	(41.16%)	0.020
Ethnicity										
Hispanic or Latino	348	(9.45%)	13,362	(4.61%)	0.190	346	(9.41%)	350	(9.52%)	0.004
Unknown	561	(15.23%)	43,071	(14.86%)	0.010	560	(15.23%)	533	(14.50%)	0.021
Race										
White	2,633	(71.47%)	229,701	(79.26%)	0.181	2,630	(71.55%)	2,620	(71.27%)	0.006
Black or African American	458	(12.43%)	26,054	(8.99%)	0.111	458	(12.46%)	473	(12.87%)	0.012
Asian	171	(4.64%)	7,829	(2.70%)	0.103	169	(4.60%)	160	(4.35%)	0.012
Other	118	(3.20%)	7,213	(2.49%)	0.043	118	(3.21%)	111	(3.02%)	0.011
Unknown	276	(7.49%)	17,290	(5.97%)	0.061	274	(7.45%)	275	(7.48%)	0.001
[a]*Body Mass Index (kg/m²) [mean (SD)]*	28.77	(6.54)	28.85	(6.54)	0.012	28.78	(6.54)	29.30	(6.50)	0.080
<18.5	223	(6.05%)	11,436	(3.95%)	0.097	222	(6.04%)	194	(5.28%)	0.033
18.5-24.9	1,150	(31.22%)	85,892	(29.64%)	0.034	1,147	(31.20%)	1,078	(29.33%)	0.041
25-29.9	1,717	(46.61%)	116,828	(40.31%)	0.127	1,710	(46.52%)	1,647	(44.80%)	0.034
30-40	1,507	(40.91%)	96,733	(33.38%)	0.156	1,502	(40.86%)	1,461	(39.74%)	0.023
>40.0	383	(10.40%)	24,846	(8.57%)	0.062	382	(10.39%)	373	(10.15%)	0.008
Persons with potential health hazards related to socioeconomic and psychosocial circumstances	188	(5.10%)	10,161	(3.51%)	0.079	187	(5.09%)	196	(5.33%)	0.011
Tobacco use	167	(4.53%)	10,086	(3.48%)	0.054	167	(4.54%)	171	(4.65%)	0.005
Nicotine dependence	545	(14.79%)	35,229	(12.16%)	0.077	544	(14.80%)	540	(14.69%)	0.003
Alcohol related disorders	220	(5.97%)	12,447	(4.29%)	0.076	219	(5.96%)	212	(5.77%)	0.008
Diseases of the circulatory system	3,097	(84.07%)	210,503	(72.63%)	0.280	3,089	(84.03%)	3,009	(81.86%)	0.058
Hypertensive diseases	2,536	(68.84%)	162,415	(56.04%)	0.267	2,529	(68.80%)	2,467	(67.11%)	0.036
Ischemic heart diseases	1,083	(29.40%)	54,726	(18.88%)	0.248	1,078	(29.33%)	1,052	(28.62%)	0.016
Heart failure	472	(12.81%)	20,888	(7.21%)	0.188	469	(12.76%)	409	(11.13%)	0.050
Diseases of arteries, arterioles and capillaries	1,003	(27.23%)	57,341	(19.79%)	0.176	998	(27.15%)	953	(25.92%)	0.028
Diabetes mellitus	1,217	(33.03%)	64,068	(22.11%)	0.246	1,211	(32.94%)	1,179	(32.07%)	0.019
Cerebrovascular diseases	609	(16.53%)	33,055	(11.41%)	0.148	607	(16.51%)	545	(14.83%)	0.046
Chronic kidney disease (CKD)	866	(23.51%)	30,751	(10.61%)	0.348	858	(23.34%)	834	(22.69%)	0.016
Primary thrombophilia	42	(1.14%)	3,295	(1.14%)	0.000	42	(1.14%)	37	(1.01%)	0.013
Other thrombophilia	57	(1.55%)	2,763	(0.95%)	0.053	56	(1.52%)	51	(1.39%)	0.011
Polycythemia vera	19	(0.52%)	1,529	(0.53%)	0.002	19	(0.52%)	20	(0.54%)	0.004
Homocystinuria	10	(0.27%)	533	(0.18%)	0.018	10	(0.27%)	12	(0.33%)	0.010
Malignant immunoproliferative diseases and certain other B-cell lymphomas	10	(0.27%)	1,395	(0.48%)	0.034	10	(0.27%)	15	(0.41%)	0.023
Multiple myeloma and malignant plasma cell neoplasms	36	(0.98%)	7,312	(2.52%)	0.118	36	(0.98%)	34	(0.92%)	0.006
Systemic lupus erythematosus (SLE)	43	(1.17%)	3,478	(1.20%)	0.003	43	(1.17%)	41	(1.12%)	0.005
Glaucoma	534	(14.50%)	38,040	(13.13%)	0.040	533	(14.50%)	515	(14.01%)	0.014
Medications										
Antithrombotic agents	2,670	(72.48%)	165,016	(56.94%)	0.330	2,662	(72.42%)	2,604	(70.84%)	0.035
Antihypertensives	981	(26.63%)	51,651	(17.82%)	0.213	975	(26.52%)	903	(24.56%)	0.045
Estrogens	260	(7.06%)	35,315	(12.19%)	0.175	260	(7.07%)	244	(6.64%)	0.017
Immunosuppressants	574	(15.58%)	41,448	(14.30%)	0.036	572	(15.56%)	523	(14.23%)	0.037
Other antineoplastics										
Alkylating agents	161	(4.37%)	20,275	(7.00%)	0.114	161	(4.38%)	163	(4.43%)	0.003
Antimetabolites	451	(12.24%)	87,502	(30.19%)	0.450	451	(12.27%)	355	(9.66%)	0.084
Plant alkaloids and other natural products	314	(8.52%)	23,558	(8.13%)	0.014	314	(8.54%)	315	(8.57%)	0.001
Cytotoxic antibiotics and related substances	269	(7.30%)	17,809	(6.14%)	0.046	269	(7.32%)	243	(6.61%)	0.028
Monoclonal antibodies	538	(14.60%)	23,234	(8.02%)	0.209	532	(14.47%)	559	(15.21%)	0.021
Radiation Oncology Treatment	675	(18.32%)	19,116	(6.60%)	0.361	668	(18.17%)	633	(17.22%)	0.025
Cancer stages										
Stage 0	10	(0.27%)	1,154	(0.40%)	0.022	10	(0.27%)	10	(0.27%)	0.000
Stage 1	74	(2.01%)	4,115	(1.42%)	0.045	73	(1.99%)	74	(2.01%)	0.002
Stage 2	43	(1.17%)	2,749	(0.95%)	0.021	42	(1.14%)	33	(0.90%)	0.024
Stage 3	93	(2.52%)	2,010	(0.69%)	0.146	88	(2.39%)	77	(2.09%)	0.020
Stage 4	62	(1.68%)	1,445	(0.50%)	0.114	61	(1.66%)	59	(1.61%)	0.004

Abbreviations: Abbreviations: SD, standard deviation; SMD, standardized mean difference; TKI, tyrosine kinase inhibitor; VEGF, vascular endothelial growth factor

[a]Data indexed at baseline was used
