# Supplementary material for: Risks of Retinal Vascular Occlusions with the Systemic Use of Tyrosine Kinase Inhibitors
Source: Ophthalmol Retina. Author manuscript; Available in PMC 2026 Sep 21. (PMC13591267; doi:10.1016/j.oret.2026.02.019)
Supplement: Supplementary file 28 [file NIHMS2194774-supplement-8.pdf]

eTable 3G: Results of the Propensity Score Matching Analysis Performed for the Risk Ratio Analysis Comparing the Non-BCR-ABL TKI (Study) Cohort to the Non-TKI (Control) Cohort

	Before Matching					After Matching				
	Study		Control		SMD	Study		Control		SMD
Number of Patients	19,786		305,867			19,784		19,784		
Age at Index	66.75	(13.78)	68.10	(13.38)	0.100	66.75	(13.78)	66.70	(14.66)	0.003
Female	9,543	(48.23%)	173,918	(56.86%)	0.173	9,542	(48.23%)	9,426	(47.64%)	0.012
Ethnicity										
Hispanic or Latino	1,272	(6.43%)	15,390	(5.03%)	0.060	1,272	(6.43%)	1,291	(6.53%)	0.004
Unknown	3,025	(15.29%)	52,388	(17.13%)	0.050	3,025	(15.29%)	2,938	(14.85%)	0.012
Race										
White	14,615	(73.87%)	241,295	(78.89%)	0.118	14,614	(73.87%)	14,490	(73.24%)	0.014
Black or African American	2,263	(11.44%)	34,584	(11.31%)	0.004	2,262	(11.43%)	2,251	(11.38%)	0.002
Asian	1,232	(6.23%)	9,037	(2.95%)	0.157	1,232	(6.23%)	1,282	(6.48%)	0.010
Other	557	(2.82%)	6,515	(2.13%)	0.044	557	(2.82%)	598	(3.02%)	0.012
Unknown	975	(4.93%)	12,230	(4.00%)	0.045	975	(4.93%)	989	(5.00%)	0.003
[a]*Body Mass Index (kg/m²) [mean (SD)]*	27.36	(6.22)	28.56	(6.63)	0.187	27.36	(6.22)	27.75	(6.40)	0.063
<18.5	2,227	(11.26%)	22,824	(7.46%)	0.131	2,226	(11.25%)	2,209	(11.17%)	0.003
18.5-24.9	9,016	(45.57%)	112,937	(36.92%)	0.176	9,015	(45.57%)	9,085	(45.92%)	0.007
25-29.9	10,733	(54.25%)	147,924	(48.36%)	0.118	10,731	(54.24%)	10,670	(53.93%)	0.006
30-40	7,888	(39.87%)	121,522	(39.73%)	0.003	7,887	(39.87%)	7,605	(38.44%)	0.029
>40.0	2,083	(10.53%)	34,128	(11.16%)	0.020	2,083	(10.53%)	2,003	(10.12%)	0.013
Persons with potential health hazards related to socioeconomic and psychosocial circumstances	1,316	(6.65%)	20,001	(6.54%)	0.005	1,316	(6.65%)	1,234	(6.24%)	0.017
Tobacco use	1,040	(5.26%)	18,995	(6.21%)	0.041	1,040	(5.26%)	1,017	(5.14%)	0.005
Nicotine dependence	2,910	(14.71%)	48,257	(15.78%)	0.030	2,909	(14.70%)	2,957	(14.95%)	0.007
Alcohol related disorders	1,216	(6.15%)	18,712	(6.12%)	0.001	1,216	(6.15%)	1,171	(5.92%)	0.010
Diseases of the circulatory system	17,597	(88.94%)	260,327	(85.11%)	0.114	17,595	(88.94%)	17,534	(88.63%)	0.010
Hypertensive diseases	13,764	(69.56%)	210,018	(68.66%)	0.020	13,762	(69.56%)	13,703	(69.26%)	0.006
Ischemic heart diseases	6,155	(31.11%)	86,698	(28.34%)	0.060	6,154	(31.11%)	6,022	(30.44%)	0.014
Heart failure	3,343	(16.90%)	43,349	(14.17%)	0.075	3,342	(16.89%)	3,249	(16.42%)	0.013
Diseases of arteries, arterioles and capillaries	5,921	(29.93%)	91,223	(29.82%)	0.002	5,920	(29.92%)	5,800	(29.32%)	0.013
Diabetes mellitus	6,494	(32.82%)	95,782	(31.31%)	0.032	6,494	(32.82%)	6,524	(32.98%)	0.003
Cerebrovascular diseases	4,228	(21.37%)	56,006	(18.31%)	0.077	4,226	(21.36%)	4,143	(20.94%)	0.010
Chronic kidney disease (CKD)	4,647	(23.49%)	56,616	(18.51%)	0.122	4,646	(23.48%)	4,606	(23.28%)	0.005
Primary thrombophilia	500	(2.53%)	4,947	(1.62%)	0.064	499	(2.52%)	474	(2.40%)	0.008
Other thrombophilia	619	(3.13%)	4,537	(1.48%)	0.110	618	(3.12%)	570	(2.88%)	0.014
Polycythemia vera	547	(2.76%)	1,997	(0.65%)	0.163	546	(2.76%)	474	(2.40%)	0.023
Homocystinuria	37	(0.19%)	608	(0.20%)	0.003	37	(0.19%)	40	(0.20%)	0.003
Malignant immunoproliferative diseases and certain other B-cell lymphomas	806	(4.07%)	2,174	(0.71%)	0.221	804	(4.06%)	668	(3.38%)	0.036
Multiple myeloma and malignant plasma cell neoplasms	713	(3.60%)	11,298	(3.69%)	0.005	713	(3.60%)	685	(3.46%)	0.008
Systemic lupus erythematosus (SLE)	236	(1.19%)	4,734	(1.55%)	0.031	236	(1.19%)	232	(1.17%)	0.002
Glaucoma	3,560	(17.99%)	61,775	(20.20%)	0.056	3,560	(17.99%)	3,512	(17.75%)	0.006
Medications										
Antithrombotic agents	15,851	(80.11%)	213,715	(69.87%)	0.238	15,849	(80.11%)	15,857	(80.15%)	0.001
Antihypertensives	5,540	(28.00%)	80,517	(26.32%)	0.038	5,538	(27.99%)	5,403	(27.31%)	0.015
Estrogens	1,861	(9.41%)	36,267	(11.86%)	0.080	1,860	(9.40%)	1,767	(8.93%)	0.016
Immunosuppressants	5,132	(25.94%)	52,700	(17.23%)	0.213	5,130	(25.93%)	5,098	(25.77%)	0.004
Other antineoplastics										
Alkylating agents	3,346	(16.91%)	30,536	(9.98%)	0.204	3,344	(16.90%)	3,267	(16.51%)	0.010
Antimetabolites	5,718	(28.90%)	97,961	(32.03%)	0.068	5,717	(28.90%)	5,198	(26.27%)	0.059
Plant alkaloids and other natural products	2,585	(13.06%)	36,042	(11.78%)	0.039	2,585	(13.07%)	2,563	(12.95%)	0.003
Cytotoxic antibiotics and related substances	2,099	(10.61%)	22,598	(7.39%)	0.113	2,098	(10.60%)	2,072	(10.47%)	0.004
Monoclonal antibodies	5,346	(27.02%)	46,733	(15.28%)	0.291	5,344	(27.01%)	5,367	(27.13%)	0.003
Radiation Oncology Treatment	4,375	(22.11%)	35,114	(11.48%)	0.287	4,373	(22.10%)	4,475	(22.62%)	0.012
Cancer stages										
Stage 0	63	(0.32%)	2,184	(0.71%)	0.055	63	(0.32%)	45	(0.23%)	0.017
Stage 1	431	(2.18%)	7,978	(2.61%)	0.028	431	(2.18%)	416	(2.10%)	0.005
Stage 2	303	(1.53%)	5,077	(1.66%)	0.010	303	(1.53%)	287	(1.45%)	0.007
Stage 3	395	(2.00%)	3,883	(1.27%)	0.057	395	(2.00%)	434	(2.19%)	0.014
Stage 4	679	(3.43%)	3,849	(1.26%)	0.144	679	(3.43%)	685	(3.46%)	0.002

Abbreviations: SD, standard deviation; SMD, standardized mean difference; TKI, tyrosine kinase inhibitor

[a]*Data indexed at baseline was used*
