# Supplementary material for: Risks of Retinal Vascular Occlusions with the Systemic Use of Tyrosine Kinase Inhibitors
Source: Ophthalmol Retina. Author manuscript; Available in PMC 2026 Sep 21. (PMC13591267; doi:10.1016/j.oret.2026.02.019)
Supplement: Supplementary file 29 [file NIHMS2194774-supplement-9.pdf]

eTable 3H: Results of the Propensity Score Matching Analysis Performed for the Hazard Ratio Analysis Comparing the Non-BCR-ABL TKI (Study) Cohort to the Non-TKI (Control) Cohort

	Before Matching					After Matching				
	Study		Control		SMD	Study		Control		SMD
Number of Patients	18,112		302,836			18,107		18,107		
Age at Index	67.13	(13.66)	68.18	(13.34)	0.077	67.13	(13.66)	66.95	(14.39)	0.013
Female	8,788	(48.52%)	173,248	(57.21%)	0.175	8,787	(48.53%)	8,697	(48.03%)	0.010
Ethnicity										
Hispanic or Latino	1,118	(6.17%)	14,663	(4.84%)	0.058	1,118	(6.17%)	1,130	(6.24%)	0.003
Unknown	2,754	(15.21%)	50,588	(16.70%)	0.041	2,753	(15.20%)	2,699	(14.91%)	0.008
Race										
White	13,483	(74.44%)	241,107	(79.62%)	0.123	13,478	(74.44%)	13,480	(74.45%)	0.000
Black or African American	2,034	(11.23%)	33,037	(10.91%)	0.010	2,034	(11.23%)	1,982	(10.95%)	0.009
Asian	1,112	(6.14%)	8,582	(2.83%)	0.160	1,112	(6.14%)	1,153	(6.37%)	0.009
Other	480	(2.65%)	6,195	(2.05%)	0.040	480	(2.65%)	503	(2.78%)	0.008
Unknown	877	(4.84%)	11,814	(3.90%)	0.046	877	(4.84%)	862	(4.76%)	0.004
[a]*Body Mass Index (kg/m²) [mean (SD)]*	27.44	(6.20)	28.67	(6.64)	0.192	27.44	(6.20)	27.86	(6.40)	0.066
<18.5	2,003	(11.06%)	22,033	(7.28%)	0.131	2,002	(11.06%)	1,958	(10.81%)	0.008
18.5-24.9	8,288	(45.76%)	111,473	(36.81%)	0.183	8,283	(45.74%)	8,321	(45.95%)	0.004
25-29.9	9,978	(55.09%)	148,058	(48.89%)	0.124	9,973	(55.08%)	9,828	(54.28%)	0.016
30-40	7,382	(40.76%)	122,901	(40.58%)	0.004	7,380	(40.76%)	7,297	(40.30%)	0.009
>40.0	1,948	(10.76%)	34,798	(11.49%)	0.023	1,948	(10.76%)	1,890	(10.44%)	0.010
Persons with potential health hazards related to socioeconomic and psychosocial circumstances	1,255	(6.93%)	20,199	(6.67%)	0.010	1,254	(6.93%)	1,222	(6.75%)	0.007
Tobacco use	1,007	(5.56%)	19,380	(6.40%)	0.035	1,007	(5.56%)	1,002	(5.53%)	0.001
Nicotine dependence	2,700	(14.91%)	48,699	(16.08%)	0.032	2,700	(14.91%)	2,659	(14.68%)	0.006
Alcohol related disorders	1,100	(6.07%)	18,502	(6.11%)	0.002	1,100	(6.07%)	1,138	(6.28%)	0.009
Diseases of the circulatory system	16,156	(89.20%)	258,395	(85.33%)	0.116	16,151	(89.20%)	16,108	(88.96%)	0.008
Hypertensive diseases	12,707	(70.16%)	209,303	(69.11%)	0.023	12,702	(70.15%)	12,596	(69.56%)	0.013
Ischemic heart diseases	5,810	(32.08%)	87,420	(28.87%)	0.070	5,807	(32.07%)	5,733	(31.66%)	0.009
Heart failure	3,096	(17.09%)	42,898	(14.17%)	0.081	3,094	(17.09%)	3,049	(16.84%)	0.007
Diseases of arteries, arterioles and capillaries	5,669	(31.30%)	91,717	(30.29%)	0.022	5,668	(31.30%)	5,483	(30.28%)	0.022
Diabetes mellitus	6,008	(33.17%)	95,653	(31.59%)	0.034	6,006	(33.17%)	5,963	(32.93%)	0.005
Cerebrovascular diseases	3,914	(21.61%)	56,128	(18.53%)	0.077	3,912	(21.60%)	3,872	(21.38%)	0.005
Chronic kidney disease (CKD)	4,336	(23.94%)	56,112	(18.53%)	0.133	4,333	(23.93%)	4,303	(23.76%)	0.004
Primary thrombophilia	460	(2.54%)	4,900	(1.62%)	0.065	459	(2.53%)	424	(2.34%)	0.013
Other thrombophilia	557	(3.08%)	4,319	(1.43%)	0.111	556	(3.07%)	531	(2.93%)	0.008
Polycythemia vera	541	(2.99%)	2,025	(0.67%)	0.174	539	(2.98%)	474	(2.62%)	0.022
Homocystinuria	38	(0.21%)	602	(0.20%)	0.002	38	(0.21%)	32	(0.18%)	0.008
Malignant immunoproliferative diseases and certain other B-cell lymphomas	801	(4.42%)	2,168	(0.72%)	0.236	797	(4.40%)	665	(3.67%)	0.037
Multiple myeloma and malignant plasma cell neoplasms	671	(3.70%)	11,028	(3.64%)	0.003	671	(3.71%)	643	(3.55%)	0.008
Systemic lupus erythematosus (SLE)	221	(1.22%)	4,759	(1.57%)	0.030	221	(1.22%)	202	(1.12%)	0.010
Glaucoma	3,353	(18.51%)	62,069	(20.50%)	0.050	3,353	(18.52%)	3,265	(18.03%)	0.013
Medications										
Antithrombotic agents	14,496	(80.04%)	210,414	(69.48%)	0.245	14,491	(80.03%)	14,381	(79.42%)	0.015
Antihypertensives	5,129	(28.32%)	80,107	(26.45%)	0.042	5,126	(28.31%)	5,075	(28.03%)	0.006
Estrogens	1,772	(9.78%)	36,702	(12.12%)	0.075	1,771	(9.78%)	1,704	(9.41%)	0.013
Immunosuppressants	4,698	(25.94%)	52,251	(17.25%)	0.212	4,695	(25.93%)	4,625	(25.54%)	0.009
Other antineoplastics										
Alkylating agents	3,017	(16.66%)	28,970	(9.57%)	0.211	3,012	(16.63%)	2,985	(16.49%)	0.004
Antimetabolites	5,107	(28.20%)	95,684	(31.60%)	0.074	5,104	(28.19%)	4,587	(25.33%)	0.065
Plant alkaloids and other natural products	2,321	(12.81%)	33,722	(11.14%)	0.052	2,321	(12.82%)	2,270	(12.54%)	0.008
Cytotoxic antibiotics and related substances	1,851	(10.22%)	22,242	(7.34%)	0.102	1,850	(10.22%)	1,816	(10.03%)	0.006
Monoclonal antibodies	4,942	(27.29%)	45,679	(15.08%)	0.302	4,938	(27.27%)	5,016	(27.70%)	0.010
Radiation Oncology Treatment	3,883	(21.44%)	33,492	(11.06%)	0.284	3,878	(21.42%)	3,983	(22.00%)	0.014
Cancer stages										
Stage 0	62	(0.34%)	2,159	(0.71%)	0.051	62	(0.34%)	67	(0.37%)	0.005
Stage 1	412	(2.27%)	7,820	(2.58%)	0.020	412	(2.28%)	392	(2.16%)	0.007
Stage 2	281	(1.55%)	4,881	(1.61%)	0.005	281	(1.55%)	266	(1.47%)	0.007
Stage 3	361	(1.99%)	3,590	(1.19%)	0.065	361	(1.99%)	403	(2.23%)	0.016
Stage 4	561	(3.10%)	3,160	(1.04%)	0.145	561	(3.10%)	585	(3.23%)	0.008

Abbreviations: SD, standard deviation; SMD, standardized mean difference; TKI, tyrosine kinase inhibitor

[a]*Data indexed at baseline was used*
